# Comparative effectiveness of antibiotic prophylaxis for preventing serious adverse events after primary total hip arthroplasty: a systematic review and network meta-analysis of randomized trials

**DOI:** 10.2340/17453674.2025.44482

**Published:** 2025-08-19

**Authors:** Armita A ABEDI, Jacob M SVENSSON, Alma B PEDERSEN, Claus VARNUM, Sabrina M NIELSEN, Jens H LAIGAARD, Robin CHRISTENSEN, Søren OVERGAARD

**Affiliations:** 1 Department of Orthopaedic Surgery and Traumatology, Copenhagen University Hospital, Bispebjerg; 2 Department of Clinical Medicine, Faculty of Health and Medical Sciences, University of Copenhagen, Copenhagen; 3 Section for Biostatistics and Evidence-Based Research, the Parker Institute, Bispebjerg and Frederiksberg Hospital, Copenhagen; 4 Department of Clinical Epidemiology, Aarhus University Hospital, Aarhus; 5 Department of Clinical Medicine, Aarhus University, Aarhus; 6 Department of Orthopaedic Surgery, Lillebaelt Hospital, Vejle; 7 Department of Regional Health Research, University of Southern Denmark, Odense; 8 Research Unit of Rheumatology, Department of Clinical Research, University of Southern Denmark, Odense University Hospital, Denmark

## Abstract

**Background and purpose:**

The optimal duration of antibiotic prophylaxis for reducing serious adverse events (SAEs) after total hip arthroplasty (THA) is unclear. We aimed to assess the comparative effectiveness of different strategies of antibiotic prophylaxis in preventing SAEs after THA.

**Methods:**

We searched Medline, Embase, CENTRAL, and the Clinical Trial Registration Database for randomized controlled trials evaluating antibiotic prophylaxis in patients undergoing primary THA. Two authors independently screened, extracted data, and assessed the risk of bias. We defined SAEs as prosthetic joint infections, other serious infections, major cardiovascular events, venous thromboembolisms, or mortality. The primary summary measures were odds ratios (ORs) with 95% confidence intervals (CI). The evidence was assessed using the confidence in network meta-analysis (CINeMA) framework.

**Results:**

Of 6,131 identified citations, 10 trials of 2-group comparisons were included, involving 9,106 patients. Duration of antibiotics was grouped as follows: placebo (3), a single dose (3), multiple doses ≤ 24 hours (6), multiple doses (> 1 day) (6), and bone cement with antibiotics (2). Compared with placebo, point estimates suggest lower odds of SAEs after THA for most antibiotic strategies, except multiple doses > 1 day. Multiple doses showed no clear evidence of superiority to single dose: OR (multiple doses ≤ 24 hours) = 0.87 (CI 0.20–3.73; very low) or over more days (> 1 day) OR = 0.40 (CI 0.07–2.42; very low) nor were multiple doses > 1 day superior to multiple doses ≤ 24 hours, OR = 0.46 (0.11–1.90; very low).

**Conclusion:**

Relative to placebo, point estimates suggested that most antibiotic prophylaxis regimens may reduce SAEs after THA, with no clear evidence of added benefit from multiple doses. These findings should be interpreted with caution due to the lack of precision and the corresponding very low certainty of evidence for some comparisons.

Total hip arthroplasty (THA) is one of the most common orthopedic procedures with over 600,000 performed annually in Europe and the United States [1,2]. Although successful, serious adverse events (SAEs) do occur. They include surgical site infections (SSIs), prosthetic joint infections (PJIs), and death, among other complications [3-6]. As SAEs are associated with increased morbidity, prolonged hospitalization, and increased healthcare costs [4,5,7], prevention is crucial [8]. Perioperative antibiotic prophylaxis is a well-established part of standard care to reduce the risk of SSI [9-11]. However, there remains a lack of consensus regarding its optimal perioperative duration. Guidelines from the US Centers for Disease Control and Prevention (CDC) and the World Health Organization (WHO) advocate for 1 single preoperative dose of prophylactic antibiotic [12,13]. Other guidelines recommend up to 24 hours of continued antibiotic prophylaxis [14-17].

The duration of antibiotic use impacts antimicrobial resistance, an increasing global health threat. In response, the WHO has endorsed a Global Action Plan, stressing optimized antimicrobial usage [18]. It is therefore crucial to establish the optimal duration of antibiotic prophylaxis, especially for common surgical procedures [19-22]. Postoperatively, healthcare-related infections, such as pneumonia and urinary tract infections, can escalate to serious infections including septic shock, prolonged hospitalization, and increased mortality rates [23,24]. These infections also heighten the risk of venous thromboembolism (VTE) and cardiovascular events [25-27]. Thus, it is important to consider all potential SAEs when determining the optimal duration of antibiotic prophylaxis, not just SSIs and PJIs.

Therefore, we aimed to compare the effectiveness of different antibiotic prophylaxis durations, specifically evaluating whether extended antibiotic prophylaxis provides additional benefit in preventing SAEs within 1 year following primary THA. Our outcomes included (i) a composite SAE measure comprising PJI, serious infections other than SSI/PJI, major adverse cardiovascular events (MACE), VTE, and mortality, (ii) SSI, and (iii) the individual components of SAE. Where possible, outcomes were assessed within 90 days.

## Methods

### Search strategy and selection criteria

Our review followed the preferred reporting items for systematic reviews and meta-analyses protocols (PRISMA-P) statement [28] and the PRISMA extension statement for reporting systematic reviews incorporating network meta-analyses of healthcare interventions [29]. The protocol was registered with PROSPERO (CRD42022385597); the prespecified protocol is available in Supplementary data. Randomized trials with a follow-up period of at least 90 days comparing antibiotic prophylaxis practices among adult patients (≥ 18 years of age) undergoing primary THA for any reason were considered eligible. Eligible interventions included any form of antibiotic therapy, placebo, or no prophylaxis, in any dosage, including frequency, duration, and route of administration. There was no language limitation, and publication year restrictions were not applied.

We searched Medline via PubMed from 1966, EMBASE via OVID from 1980, the Cochrane Central Register of Controlled Trials (CENTRAL), and the Clinical Trial Registration Database indexed from inception to December 22^th^, 2022 (Supplementary Table S1). Additional articles were identified by citation tracking of previously published systematic reviews, meta-analyses, and the included trials.

Two review authors (AAA and JMS) independently screened the titles and abstracts of all identified trials. Upon agreement between the two, full-text versions were obtained and screened for eligibility independently. Any disagreements were resolved by discussion or by a third author (SO or RC). We considered all publications related to each of the included trials.

### Data analysis

Data was extracted independently by two reviewers (AAA and either JMS or JHL). Any disagreements were resolved by discussion with a fourth reviewer (SO or RC). In line with our protocol, the anticipated intervention groups and corresponding nodes included in the network were: placebo, single dose, multiple doses ≤ 24 hours, multiple doses > 1 day, and antibiotic-enriched bone cement (antibiotic cement). Following current guidelines, we grouped trial arms that applied antibiotic prophylaxis durations exceeding 24 hours, regardless of the number of days. Information on the mean age (with standard deviation where reported) and sex distribution of participants in each trial was extracted to describe the study populations and aid in assessing generalizability, given that these factors may influence infection risk after THA.

The following outcomes were considered in prioritized order: the primary outcome was a composite SAE (including PJI, any other serious infection, MACE, VTE, and death) within 1 year after THA. The secondary outcomes were SSI and the subcomponents of SAE. The follow-up period for assessment of outcomes was at least 90 days and up to 1 year after primary THA.

1. SAEs were assessed according to the International Conference on Harmonization of Technical Requirements for Registration of Pharmaceuticals for Human Use, document E6R [30]. For this review, we defined SAE as a composite of PJI, other serious infections, MACE, VTE, and mortality. All SAEs reported in the eligible trials were considered, regardless of a causal relationship with treatment. Events were included if they met commonly accepted SAE criteria (e.g., prolonged hospitalization, life-threatening, or fatal) and were reported by the study authors as such, even if this was not explicitly linked to the intervention.
2. SSIs were defined according to the widely accepted Centers for Disease Control and Prevention (CDC) criteria as either superficial (restricted to the skin or subcutaneous tissue), deep (involving the muscle or fascia layers), or organ space (involving the internal anatomic region where the operation was performed) [31]. The CDC’s SSI definition, from 1992, replaced older criteria, possibly used more widely in the 1980s and 1990s. There is evidence that either system to define SSI may provide important information comparable to those attained by the CDC criteria [32]. We considered all available evidence in the evidence syntheses. If a study did not specify an SSI as a PJI, the outcome was categorized solely as an SSI.
3. Serious infections were defined as any infection other than SSI and PJI, associated with admission to, acquired in, or treated at a hospital, or death due to infection.
4. MACE was defined as a composite endpoint of myocardial infarction, stroke, or cardiovascular death [33-35].
5. VTE was defined as a composite endpoint of deep venous thrombosis (DVT) or pulmonary embolism.
6. Mortality was defined as any death occurring after primary THA.

While the original primary outcomes of the included trials were typically PJI and/or SSI, the composite SAE outcome was prespecified to allow for harmonized and clinically meaningful comparisons across studies with varying definitions. A detailed summary of the original primary outcomes is provided in Supplementary Table S7.

Two authors (AAA and either JMS or JHL) assessed each included study using the revised Cochrane “Risk of bias” tool for randomized trials (RoB 2.0) [36]. The overall risk of bias for each trial was categorized as low (low risk across all domains), high (high risk in 1 or more domains), or unclear (unclear risk in 1 or more domains and no high-risk domains). A risk of bias table was completed for all outcomes.

Results of the intention-to-treat (ITT) populations were applied where possible. For the standard contrast-based meta-analyses, the odds ratios (OR) and 95% confidence intervals (CIs) were calculated for each trial (assuming rare event rates below 1%) with the use of the Peto one-step ORs (with 95% CI) [37]. We used forest plots to display the individual trial estimates. Statistical heterogeneity was assessed by evaluating the extent of overlap of confidence intervals and based on the inconsistency index (I^2^) [38]. We performed arm-based network meta-analyses (summarized based on ORs) mixed-effects logistic regression to assess the comparative effectiveness [39,40]. The OR with the 95% CI was the primary summary measure. We considered 95% CIs that did not include 1 as statistically significant. In contrast, the range of equivalence was tentatively defined concerning OR values that translate into an OR greater than 1.05 or below 0.95 representing a clinically important effect. For ORs greater than 1, we compared the 95% CI with the opposite half of the range of equivalence (0.95–1.05). The standard contrast-based meta-analyses were performed using Review Manager Software (version 5.3, Nordic Cochrane Center, Cochrane Collaboration, Copenhagen, Denmark), and for the main network meta-analyses SAS Studio (Release: 3.8; SAS Institute Inc, Cary, NC, USA) was applied [40, 41].

We conducted network meta-analysis of serious adverse events at 365 days using a generalized linear mixed model (GLMM) framework. Specifically, a binomial model with a logit link was fitted using the PROC GLIMMIX procedure in SAS (SAS Institute Inc), estimating odds ratios (ORs) for each treatment node relative to a shared comparator. The fixed effect in the model was treatment (i.e., depicted by Nodes), and the model included random effects for trial (TrialID) and the treatment-by-trial interaction (TrialID*Node) to account for between-trial heterogeneity and within-trial correlation of outcomes. Estimates were obtained on the log-odds scale and exponentiated to yield ORs with corresponding 95% CIs. Pairwise comparisons between treatments were derived using least-squares means with appropriate contrasts.

The main network meta-analysis model estimated the summary treatment effect for each intervention (i.e., dosage duration practice), relative to others. This allowed for the clustering of patients and dose practice within trials. To answer the treatment hierarchy question of whether there exists a superior antibiotic prophylaxis duration, we ranked clinical efficacy with a visual representation of point estimates and confidence intervals comparing network meta-analysis estimates of each treatment duration against a constant comparator, along with the certainty of evidence [42,43]. To assess the impact of antibiotic-loaded bone cement on the overall findings, we conducted a sensitivity analysis excluding studies in which cement was the only form of antibiotic prophylaxis.

Credibility of the results was examined using the Confidence in Network Meta-Analysis (CINeMA), based on the following 6 domains assessed for each outcome: (i) within-study bias, (iii) reporting bias, (iii) indirectness, (v) imprecision, (v) heterogeneity, and (vi) incoherence [44,45]. In this study, when evaluating imprecision, the area of equivalence for the 95% CI was defined as a clinically important size of OR 1.2. Relative effect estimates below 0.833 and above 1.20 were considered clinically important for the judgment of imprecision. As previously stated, we used a narrow range (0.95–1.05) to interpret clinical equivalence in the results, while for CINeMA’s imprecision domain, a broader threshold (0.833–1.20) was applied to reflect what might be considered a minimum clinically important difference, as recommended by CINeMA [44].

### Ethics, registration, data sharing plan, funding, use of AI, and disclosures

All data in this systematic review and meta-analysis is accessible to the public. There was no requirement for ethics committee approval or patient consent for publication.

The study was registered at the PROSPERO database of systematic reviews on December 20^th^, 2022: CRD42022385597. The statistical code and dataset are available and can be provided upon request to the corresponding author. No individual participant data was used; raw data presented in the cited manuscripts was applied. The first author affirms that the manuscript provides an honest, accurate, and transparent account of the study being reported; that no important aspects of the study have been excluded; and that any deviations from the original study plan and registration have been explained. There is no financial or other support explicitly donated for this specific systematic review and network meta-analysis. The study was indirectly funded by the Novo Nordisk Foundation (NNF20OC0065693), Medicine Fund of the Danish Regions (Regionernes Medicin- og Behandlingspulje; EMN-2020-00030), and the Danish Rheumatism Association (Gigtforeningen; R201-A7259). The Section for Biostatistics and Evidence-Based Research, the Parker Institute, Bispebjerg, and Frederiksberg Hospital are supported by a core grant from the Oak Foundation (OCAY-18-774-OFIL). The funders of the source had no role in study design, data collection, data analysis, data interpretation, or writing of the report. Complete disclosure of interest forms according to ICMJE are available on the article page, doi: 10.2340/17453674.2025.44482

## Results

**Figure 1:**
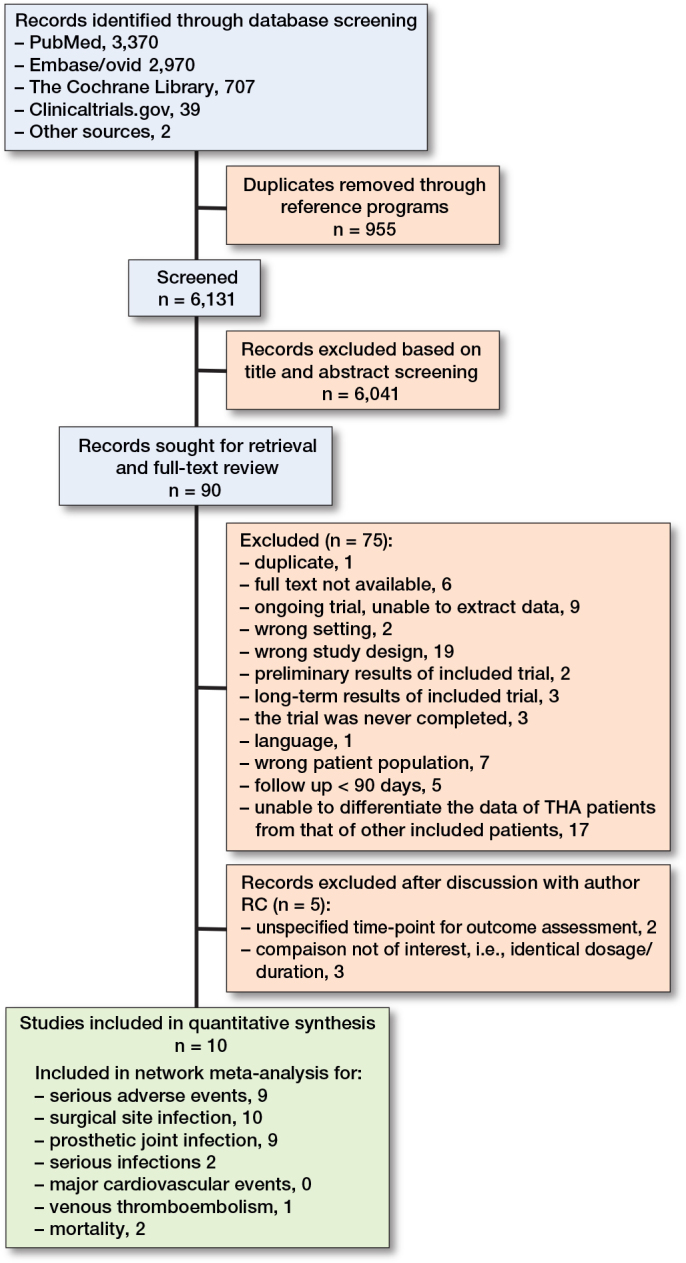
Flowchart of study selection.

The systematic search provided a total of 6,131 possible records. We screened all records for title and abstract and reviewed 90 full texts. 10 trials with 9,106 patients evaluating 5 different dosage practices were included in the quantitative evidence synthesis (Figure 1) [46-55]. The main reasons for exclusion were incompatible study designs for our inclusion criteria (n = 19) and studies where data extraction for THA patients from the pool of other included patients proved unattainable (n = 17). All individual reasons for exclusion can be found in Supplementary Table S2.

The included trials were published between 1973 and 1994; it was therefore not feasible to contact the original investigators in case of missing outcome data. The follow-up period across the trials varied from 6 to 24 months (Table 1). 9 studies reported on SAEs and 10 studies on SSI [46-55]. Due to inconsistent reporting or definitions of SAEs or SSIs in the included studies, a qualitative presentation of the events observed in each study is listed in Supplementary Table S3. One study reported VTE as an outcome [53] and 2 studies reported outcomes considered as serious infections, including septic complications and septicemia [46,54]. Two studies reported mortality as an outcome [53,54]. Moreover, in 6 studies, mortality data was provided without specifying the treatment arm the patients had been randomized to or the primary procedure undergone [46,48-50,52,55]. No study reported on components of MACE, and none of the prespecified variables for stratified analysis could be performed due to a lack of reporting.

**Table 1:** Characteristics of the included trials

Study	ITT (n)	Comparison (Number of participants assigned to each treatment arm)	Women	Mean age, years (SD)
Ericson et al. 1973 [48] Follow-up 6 months **^b^** Outcome: SSI Primary diagnosis: NA	118	• 14 days of cloxacillin: 1 g IM preoperatively + 1 g IM x 3 for 1 day + 0.5 g PO x 4 for 13 days+ probenecid until the 14th postoperative day (n = 60) vs • 14 days of placebo, given the same way as intervention + probenecid (n = 58)	60%	62·5 (SD not reported)
Pollard et al. 1979 [55] Follow-up: 12 months Outcome: SSI, PJI Primary diagnosis: OA 89%	310	• 1 day of cephaloridine: 1 g IV preoperatively + 1 g IM x 2 for 1 day (n = 146) vs • 14 days of flucloxacillin: 0-5 g IM preoperatively + 0.5 g IM x 4 for 1 day + 0.5 g PO x 4 for 13 days (n = 157)	59%	65·2 (8.3) **^a^**
Hill et al. 1981 [41] Follow-up: 24 months Outcomes: SSI, PJI, serious infections Primary diagnosis: NA	2,137	• 5 days of cefazolin: 1 g IV/IM x 4 per day (n = 1,070) vs • 5 days of placebo IV/IM x 4 per day (n = 1,067)	58%	64.5 (10)
Josefsson et al. 1981 [49] Follow-up: 24 months Outcome: SSI Primary diagnosis: OA 85%	1,685	• 7–14 days of cloxacillin/cephalexine/dicloxacillin/phenoxy-methylpenicillin IV/IM/PO: 0.5–1 g x 3–4 per day (n = 812) vs • Gentamicin in bone cement: 0.5 g (n = 821)	51%	69.0 (15.7) **^a^**
Gunst et al. 1984 [47] Follow-up: 12 months Outcomes: SSI, PJI Primary diagnosis: NA Cement without antibiotics	93	• 1 day of cefamandole: 1.5 g IV preoperatively + 1.5 g IV x 6 (n = 46) vs • Placebo (n = 47)	55%	Placebo: 65.1 (11.3)
Centulio et al. 1988 [51] Follow-up: 18 month Outcomes: SSI, PJI Primary diagnosis: OA 69% Cementless fixation	149	• 3 days of ceftriaxone: 2 g IV 1–2 hours preoperatively + 2 g IV every 24 hours (n = 81) vs • Single dose of ceftriaxone: 2 g IV 1–2 hours preoperatively (n = 68)	64%	3 days group: 62·8 (11.5) **^a^** Single dose: 63.6 (11.5) **^a^**
McQueen et al. 1990 [52] Follow-up: 24 months Outcomes: SSI and PJI Primary diagnosis: OA	380	• 1 day of cefuroxime: 1.5 g IV/IM preoperatively + 750 mg IV/IM x 2 per day (n = 190) vs • Cefuroxime in bone cement: 1.5 g x 1 (n = 190)	67% 79%	67 (14.5) **^a^** 69 (11)
Wymenga et al. 1992 [54] Follow-up: 13 months Outcomes: SSI, PJI, serious infections and mortality Primary diagnosis: OA 72% Cement without antibiotics	3,199	• Single-dose of cefuroxime: 1.5 g IV preoperatively (n = 1,600) vs • 1 day of cefuroxime: 1.5 g IV preoperatively + 750 mg IV x 2 per day (n = 1,599)		
Suter et al. 1994 [53] Follow-up: 12 months Outcomes: SSI, PJI, VTE, mortality Primary diagnosis: OA 87%	520	• Single-dose of teicoplanin: 400 mg IV preoperatively (n = 260) vs • 2 doses of cefamandole: 2 g IV preoperatively + 1 g IV postoperatively x 1 (n = 260)	72%	Single-dose: 66.5 (8.8) 2-dose group: 68.2 (8.1)
Mauerhan et al. 1994 [50] Follow-up: 12 months Outcomes: SSI, PJI Primary diagnosis: OA 75%	546	• 1 day of cefuroxime: 1.5 g IV preoperatively + 750 mg IV x 2 per day (n = 285) vs • 3 days of cefazolin: 1 g IV preoperatively + 1 g IV x 3 per day (n = 265)	61%	65 (19.5) **^a^**

IM = intramuscular; ITT = intention-to-treat population; IV = intravenous; NA = Not applicable. OA = osteoarthritis; PJI = prosthetic joint infection; PO = peroral; SSI = surgical site infection; VTE = venous thromboembolism.

a Estimated SDs using range ÷ 4 or grouped midpoint method.

b Data for primary diagnosis and comorbidities represents data for the entire population of the study incl. primary and revision hips and knees.

**Figure 2:**
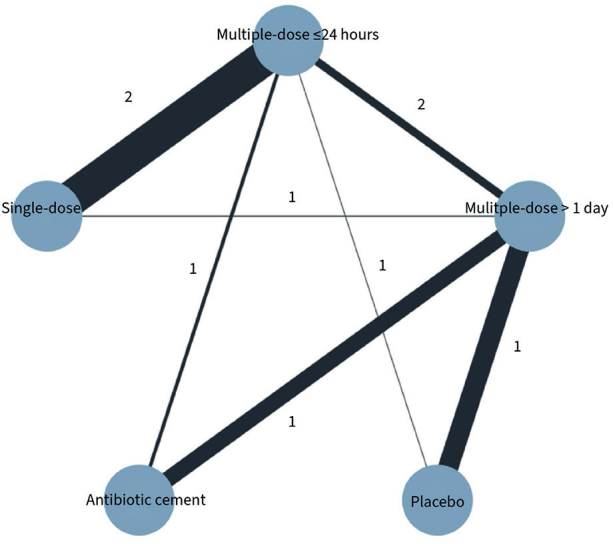
Network plot of studies included in network meta-analysis on serious adverse events. Each circle represents an intervention and is referred to as a node. Lines between nodes repre-sent direct comparisons, and their thickness is proportional to the number of participants con-tributing to each comparison.

**Figure 3:**
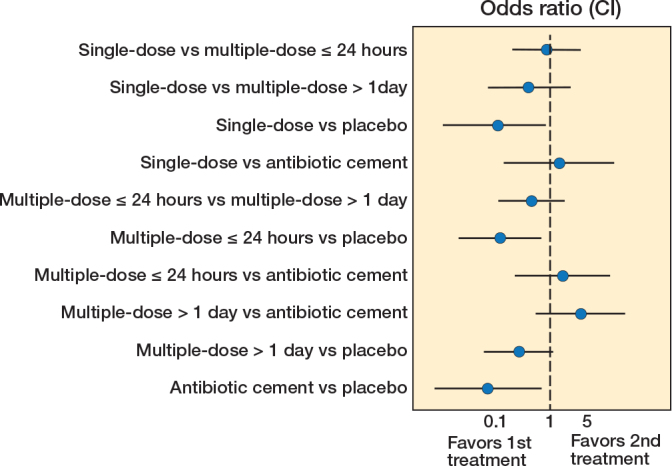
Network meta-analysis for serious adverse events within 365 days after THA.

We included 9 of 10 studies, totaling 8,988 patients, in our network meta-analysis of the primary outcome, SAEs within 1 year. One study focused solely on the secondary outcome of SSI, hence its exclusion. Most dosage duration practices were compared with multiple doses ≤ 24 hours (Figure 2). There was a protective effect of most antibiotic treatment durations compared with placebo, OR (single dose) = 0.11 (CI 0.01–0.84), OR (multiple doses ≤ 24 hours) = 0.12 (0.02–0.69), OR (antibiotic cement) = 0.07 (0.01–69) (Figure 3). While direct evidence suggested a protective effect of multiple doses > 1 day compared with placebo, this finding did not reach significance in the network meta-analysis (Table 2). We found no clear evidence suggesting that multiple doses ≤ 24 hours or treatments lasting multiple days offer a potential benefit in reducing the odds of SAEs compared with a single dose. Furthermore, no significant decrease was found between multiple doses > 1 day and a single dose. Compared with placebo, point estimates for antibiotic cement suggested the lowest odds for SAEs, followed by single dose, multiple doses ≤ 24 hours, and multiple doses > 1 day. This is presented in a visual treatment hierarchy comparing antibiotic practices against placebo as the common comparator (Supplementary Figure S5).

**Table 2:** Estimates of effects and quality ratings for comparison of antibiotic prophylaxis dosage regimens for prevention of SAEs 365 days after primary THA

Comparison of regimens	Direct evidence Odds ratio (CI)	Network meta-analysis Odds ratio (CI)	Certainty of evidence
Single-dose vs multiple-dose ≤ 24 hours	1.24 (0.86–1.81)	0.87 (0.20–3.73)	Very low **^a,b^**
Single-dose vs multiple-dose > 1 day	0.16 (0–8.41)	0.40 (0.07–2.42)	Very low **^a,b^**
Single-dose vs placebo	─	0.11 (0.01–0.84)	Low **^a^**
Single-dose vs antibiotic cement	─	1.50 (0.14–16.0)	Very low **^a,b^**
Multiple-dose ≤ 24 hours vs multiple-dose > 1 day	1.02 (0.20–5.09)	0.46 (0.11–1.90)	Very low **^a,b^**
Multiple-dose ≤ 24 hours vs placebo	0.19 (0.05–0.73)	0.12 (0.02–0.69)	Low **^a^**
Multiple-dose ≤ 24 hours vs antibiotic cement	0.51 (0.05–4.95)	1.73 (0.22–13.7)	Very low **^a,b^**
Multiple-dose > 1 day vs antibiotic cement	3.91 (1.26–12.2)	3.77 (0.54–26.1)	Low **^a,b,c^**
Multiple-dose > 1 day vs placebo	0.51 (0.39–0.67)	0.27 (0.06–1.16)	Low **^a^**
Antibiotic cement vs placebo	─	0.07 (0.01–0.69)	Low **^a,c^**

a Within-study bias.

b Imprecision.

c Heterogeneity.

CI: 95% confidence interval.

Regarding our secondary outcomes, the point estimates for all active antibiotic treatment durations suggested reduced odds of SSI and PJI compared with placebo (Supplementary Table S5). No significant results were found when comparing the effectiveness of the different active antibiotic prophylaxis durations, suggesting no differences in their protective effects for SSI and PJI prevention. Network meta-analysis could not be performed for serious infections, MACE, VTE, and mortality outcomes due to missing or unavailable data. The direct evidence concerning mortality outcome suggests that the difference in OR between a single dose and multiple doses ≤ 24 hours was non-significant, OR 1.18 (CI 0.77–1.80). For events within 90 days after primary THA, network meta-analysis was only feasible for the outcome SSI. This suggests that both ≤ 24-hour and > 1-day multidose prophylaxis may be superior to placebo, with no significant difference between the 2 durations. The sensitivity analysis, excluding studies with antibiotic-loaded bone cement, confirmed the main findings within 365 days: all systemic antibiotic prophylaxis durations were significantly more effective than placebo, but there was no significant difference between the various durations. These results are presented in Supplementary Table S8.

The overall confidence in network meta-analyses was rated low to very low for all individual outcomes, mainly due to a high risk of bias or imprecision (Table 2, Supplementary Tables S5 and S6). The high risk of bias assessments was primarily due to deviations from intended interventions, as the trialists did not assess the ITT population. Additionally, several studies exhibited incomplete outcome data and lacked clarity in reporting the randomization process. Reporting bias was evaluated with some concern for all outcome assessments due to data unavailability and thus an inability to retrieve trial protocols. The domain of indirectness, evaluating transitivity, raised some concerns across all included studies. This was attributed to the limited availability of studies for assessing effect modifier distributions across comparisons and only a moderate connection between interventions within the network. Furthermore, we found a degree of incoherence for multiple doses > 1 day, representing a difference between direct and indirect estimates, although both pointed in the same direction (Supplementary Table S5).

## Discussion

We aimed to assess the comparative effectiveness of different strategies of antibiotic prophylaxis in preventing SAEs after THA. The point estimates suggested that most prophylactic antibiotic dosage durations may reduce the risk of serious adverse events compared with placebo; however, the certainty of evidence for these findings was very low. We did not find evidence to support the anticipated superior effect of one duration strategy over another among the 4 predefined antibiotic regimens: a single dose, multiple doses ≤ 24 hours, multiple doses > 1 day, and antibiotic-loaded bone cement. This is, to our knowledge, the first comparative effectiveness evidence evaluating a broader set of SAEs in this context. While prior studies have focused exclusively on SSIs and PJIs [56-58], our approach aimed to capture complications with significant clinical implications for patient safety.

Prolonged postoperative antibiotic prophylaxis has consequences for the patient and society. Notably, the risk of adverse events associated with prolonged antibiotic use includes disruption of the normal microbiome, acute kidney injury, opportunistic infections such as *Clostridium difficile* infection, and multidrug-resistant bacteria [59-61]. Optimized antimicrobial stewardship to reduce unnecessary antibiotic use is therefore critical. Antibiotic stewardship programs have demonstrated to significantly reduce infections and colonization with antibiotic-resistant bacteria, including *Clostridium difficile,* among hospitalized patients [62]. These findings underscore the importance of stewardship initiatives in reducing the burden of antibiotic-resistant infections. Furthermore, given that prolonged antibiotic prophylaxis increases systemic toxicity and adverse drug events in up to 20% of hospitalized patients receiving antibiotics for ≥ 24 hours [63], establishing optimal antibiotic durations for THA is essential for patient safety and mitigating resistance.

Although other studies have not included SAEs, our findings aligned with 3 meta-analyses on antibiotic prophylaxis practices in THA, which found no superiority of postoperative antibiotic durations in preventing SSI [56-58]. The most recent meta-analysis focused on randomized controlled trials of antibiotics and antiseptics in THA and total knee arthroplasty also supports our study, demonstrating that preoperative systemic antibiotic prophylaxis significantly reduces infection risk compared with placebo [58].

Another recent large meta-analysis, which included 289,926 total knee arthroplasties from multiple registries, found that the incidence of PJI was similar when antibiotic-loaded bone cement was used, regardless of the doses of systemic antibiotic prophylaxis [64]. Although the studies in our meta-analysis did not combine antibiotics in cement with systemic antibiotics, this finding in a knee population further suggests that a single dose of systemic prophylactic antibiotic may be sufficient.

Our findings aligned with those of previous studies, predominantly reflecting outcomes related to PJI, a common focus in existing research. Although our primary aim was to assess the impact of antibiotic prophylaxis duration on the broader spectrum of SAEs, the majority of available studies reported outcomes specifically on PJI. This emphasis likely influenced our results, as the limited data on other SAE components restricted our ability to observe differential effects across the full intended range of SAEs. This pattern underscores the need for further research that encompasses a more comprehensive evaluation of SAE components in the context of antibiotic prophylaxis.

### Strengths

We opted for SAEs as our primary outcome measure instead of focusing solely on SSI or PJI, as we believe patient safety should address a spectrum of serious infectious complications, along with MACE, VTE, and mortality, in assessing the optimal antibiotic prophylaxis duration. Another notable strength is that we predefined our methods and registered the study protocol following standardized guidelines [29]. We applied network meta-analysis to compare all available evidence from randomized controlled trials on prophylactic antibiotic durations for primary THA, integrating direct and indirect evidence [65]. The predefined time points of 90 days and 1 year after surgery were established to ensure consistency and avoid combining studies with different follow-up durations in the same analysis of outcomes. We chose these 2 different intervals based on clinical relevance: most SAEs, particularly PJIs, SSIs, and VTEs, are reported to occur within 90 days postoperatively, a time frame that is, in theory, also more biologically plausible for a causal association with the duration of antibiotic prophylaxis. However, as a proportion of PJIs are diagnosed beyond 90 days, we also included analyses at 1 year to capture these later events. Outcomes were analyzed separately by follow-up duration.

### Limitations

As already mentioned, our study encountered a notable limitation regarding missing data on baseline risk factors and important outcomes of interest, including serious infections, MACE, VTE, and mortality. We cannot exclude the possibility of bias introduced by inadequate monitoring and reporting of data. Due to a lack of consistent reporting on SAEs, we could not draw more definite conclusions. Another key limitation of this review is the age of the included trials, all of which were published between 1973 and 1994. Since then, surgical techniques, perioperative care, and infection prevention strategies have advanced, which may limit the generalizability of our findings. Furthermore, the conduct and reporting of clinical trials have changed substantially over the past decades, with more rigorous standards for randomization, outcome reporting, and risk of bias assessment being applied in more recent studies.

While our protocol aimed to assess a broad spectrum of SAEs, our ability to analyze this comprehensively was limited by the inconsistency and sparsity of outcome reporting across included studies. As a result, our findings reflect only part of the intended scope and limit the extent to which conclusions can be drawn on all predefined aspects of serious postoperative complications. An additional limitation is the heterogeneity in outcome definitions for SSI and PJI across the included studies. Most studies used combinations of clinical, radiological, and microbiological criteria; however, the lack of a standardized definition may introduce variability in outcome assessment.

In the direct pairwise meta-analyses, we used the Peto fixed-effect model. Due to sparse data, heterogeneity was frequently not estimable and often 0 for the primary outcomes. Only 1 comparison showed notable heterogeneity but given the limitations of I², particularly in sparse meta-analyses, these results should be interpreted with caution. In the network meta-analysis, heterogeneity was modeled using a generalized linear mixed model with random effects for trial and trial-by-treatment interaction. Although τ² was not explicitly reported, between-study variability was incorporated into the model and considered in CINeMA assessments and interpretation of findings. Due to the overall sparsity of data and structure of the network, sensitivity and subgroup analyses were not feasible. We accounted for heterogeneity by avoiding strong conclusions in comparisons with limited data or high inconsistency.

Due to the overall sparsity of data and the structure of the network, sensitivity and subgroup analyses were not feasible. Several comparisons were based on very few events and were connected primarily through indirect evidence. This increases the statistical instability of effect estimates and limits our ability to verify assumptions of transitivity. We accounted for heterogeneity by avoiding strong conclusions in the comparisons and, as a result, point estimates should be interpreted with caution. Incoherence was formally assessed using CINeMA and was not identified as a concern, but the limited connectivity and sample size constrain the strength of inferences that can be drawn. For one comparison (multiple doses > 1 day), direct and indirect estimates differed in magnitude but pointed in the same direction, suggesting possible quantitative incoherence, but not of a magnitude that CINeMA flagged as concerning.

We relied on summarized (aggregate) data rather than individual participant data, which limits our ability to perform stratified analyses for distinguishing between revision and primary THA. This introduces a risk of aggregation bias, which may affect the precision and applicability of our findings. The unmeasured differences between studies may contribute to inconsistency between the direct and indirect evidence. Furthermore, although this network meta-analysis followed a prespecified protocol and analysis plan, several limitations warrant caution. Many comparisons were based on sparse event data, resulting in wide confidence intervals and unstable estimates, which increases the risk of misinterpreting chance findings as meaningful effects. As illustrated in the network diagram, several treatment contrasts relied solely on indirect evidence, rendering the results more vulnerable to inconsistency and dependent on the assumption of transitivity. To help illustrate the potential variability in future studies, we included 95% prediction intervals for the primary outcome (see Supplementary Table S5), which is a particularly relevant consideration when data is limited. In a network meta-analysis like ours, the 95% prediction interval represents the expected range within which the true treatment effect of a future study, comparing 2 of the interventions, would fall with 95% probability, assuming similar conditions and patient populations. While these limitations do not invalidate the analysis, they do constrain the strength of the inferences that can reasonably be drawn.

While most patients in the included trials met our primary THA inclusion criteria, some studies included a relatively low percentage of patients undergoing hemiarthroplasty or revision surgeries [46,54,55]. This lack of differentiation is a limitation, as results ideally should be reported separately due to the higher infection risk in revision surgery [66]. Additionally, a majority of the included trials were assessed as having either unclear or high risk of bias, significantly impacting the overall data quality and posing a limitation to our study.

### Conclusion

Our findings suggested that antibiotic prophylaxis may reduce the risk of SAEs within 1 year of primary THA. The findings do not suggest that multiple-dose regimens offered additional benefit over a single preoperative dose; however, this conclusion is based on limited available evidence and should be interpreted with caution. Limited data precluded robust analysis of serious infections, MACE, VTE, and mortality; however, no difference in mortality was observed between single and multiple doses. These findings question the need for prophylactic antibiotics beyond a single preoperative dose; however, this should be interpreted with caution, given the low certainty of evidence.

*In perspective,* these findings require further research, and high-quality studies are needed to evaluate the effect of a single dose of prophylactic antibiotic versus multiple doses in preventing SAEs after THA.

### Supplementary data

Supplementary Tables S1–S8, Supplementary Figures S1–S5, protocol and search strategy are available as supplementary data on the article page, doi: 10.2340/17453674.2025.44482

## Supplementary Material



## Notes

RC and SO contributed equally to this study. AAA, ABP, CV, SMN, RC, and SO were responsible for contributions to the conception and were actively involved in planning the methodology. AAA, JM, and JHL extracted the data. AAA and RC performed the statistical analyses. The original draft was prepared by AAA. All authors contributed to data analysis and interpretation, critically revised the manuscript, gave final approval of the version to be published, and agreed to be accountable for all aspects of the work. The corresponding author attests that all listed authors meet authorship criteria and that no others meeting the criteria have been omitted. All authors had full access to all the data and responsibility for the decision to submit for publication.

Handling co-editors: Bart A Swierstra nad Jonas Ranstam

*Acta* thanks Jon Goosen and Annefloor van Enst for help with peer review of this manuscript.
