# Supplementary material for: Comparative effectiveness of antibiotic prophylaxis for preventing serious adverse events after primary total hip arthroplasty: a systematic review and network meta-analysis of randomized trials
Source: Acta Orthop. 2025 Aug 19;96:640–9. doi: 10.2340/17453674.2025.44482 (PMC12362941; doi:10.2340/17453674.2025.44482)
Supplement: Supplementary file 1 [file ActaO-96-44482-s1.pdf]

Supplemental Data

Comparative effectiveness of antibiotic prophylaxis in prevention of serious adverse events following primary total hip arthroplasty: a systematic review and network meta-analysis of randomized controlled trials

S1 Table. Search strategy from 22.12.2022, Medline, Embase, and Cochrane Library 4

S2 Table. List of excluded trials 8

S3 Table. Characteristics of the included trials 15

S4 Table. Funding and conflict of interest statements of included studies 21

S1 Figure. Funnel Plots outcomes within 365 days after surgery

Funnel plot of comparison: 1st Treatment duration vs. 2nd Treatment duration, outcome: serious adverse events 22

Funnel plot of comparison: 1st Treatment duration vs. 2nd Treatment duration, outcome: surgical site Infections 23

Funnel plot of comparison: 1st Treatment duration vs. 2nd Treatment duration, outcome: prosthetic joint infection 24

Funnel plot of comparison: 1st Treatment duration vs. 2nd Treatment, outcome: serious infections 25

Funnel plot of comparison: 1st Treatment duration vs. 2nd Treatment duration, outcome: major cardiovascular events 26

Funnel plot of comparison: 1st Treatment duration vs. 2nd Treatment duration, outcome: venous thromboembolism 26

Funnel plot of comparison: 1st Treatment duration vs. 2nd Treatment duration, outcome: Mortality 27

S2 Figure. Funnel Plots outcomes within 90 days after surgery

Funnel plot of comparison: 1st Treatment duration vs. 2nd Treatment duration, outcome: serious adverse events 28

Funnel plot of comparison: 1st Treatment duration vs. 2nd Treatment duration, outcome: surgical site Infections 29

Funnel plot of comparison: 1st Treatment duration vs. 2nd Treatment duration, outcome: prosthetic joint infection 30

Funnel plot of comparison: 1st Treatment duration vs. 2nd treatment duration, outcome: serious infections 31

Funnel plot of comparison: 1st Treatment duration vs. 2nd Treatment duration, outcome: major cardiovascular events 31

Funnel plot of comparison: 1st Treatment duration vs. 2nd Treatment duration, outcome: venous thromboembolisms 31

Funnel plot of comparison: 1st Treatment duration vs. 2nd Treatment duration, outcome: Mortality 31

S5 Table. Estimates of effects and quality ratings for comparison of treatment durations for outcomes within 365 days and 90 days after primary THA

Within 365 days

Estimates of effects and quality ratings for comparison of treatment durations to prevent serious adverse events within 365 days 32

Estimates of effects and quality ratings for comparison of treatment durations to prevent surgical site infections within 365 days 33

Estimates of effects and quality ratings for comparison of treatment durations to prevent prosthetic joint infections within 365 days 34

Estimates of effects and quality ratings for comparison of treatment durations to prevent serious infections within 365 days 35

Estimates of effects and quality ratings for comparison of treatment durations to prevent major cardiovascular events within 365 days 36

Estimates of effects and quality ratings for comparison of treatment durations to prevent venous thromboembolisms within 365 days 36

Estimates of effects and quality ratings for comparison of treatment durations to prevent mortality within 365 days 36

Within 90 days

Estimates of effects and quality ratings for comparison of treatment durations to prevent serious adverse events within 90 days 37

Estimates of effects and quality ratings for comparison of treatment durations to prevent surgical site infections within 90 days 38

Estimates of effects and quality ratings for comparison of treatment durations to prevent prosthetic joint infections within 90 days 39

Estimates of effects and quality ratings for comparison of treatment durations to prevent serious infections within 90 days 40

Estimates of effects and quality ratings for comparison of treatment durations to prevent major cardiovascular events within 90 days 40

Estimates of effects and quality ratings for comparison of treatment durations to prevent venous thromboembolisms within 90 days 40

Estimates of effects and quality ratings for comparison of treatment durations to prevent mortality within 90 days 40

S6 Table. Cochrane Risk of Bias tool 40

S3 Figure. Forest Plots outcomes within 365 days

Forest plot of odds ratio on serious adverse events comparing treatment durations 43

Forest plot of odds ratio on surgical site infections comparing treatment durations 44

Forest plot of odds ratio on prosthetic joint infections comparing treatment durations 45

Forest plot of odds ratio on serious infections comparing treatment durations 46

Forest plot of odds ratio on major cardiovascular events comparing treatment durations 47

Forest plot of odds ratio on venous thromboembolisms comparing treatment durations 47

Forest plot of odds ratio mortality comparing treatment durations 48

S4 Figure. Forest Plots outcomes within 90 days

Forest plot of odds ratio on serious adverse events comparing treatment durations 49

Forest plot of odds ratio on surgical site infections comparing treatment durations 50

Forest plot of odds ratio on prosthetic joint infections comparing treatment durations 51

Forest plot of odds ratio on serious infections comparing treatment durations 52

Forest plot of odds ratio on major cardiovascular events comparing treatment durations 52

Forest plot of odds ratio on venous thromboembolisms comparing treatment durations 52

Forest plot of odds ratio mortality comparing treatment durations 52

S5 Figure. Point estimates and 95% confidence intervals 53

S7 Table Outcomes and outcome definitions reported in eligible trials 54

S8 Table Sensitivity Analysis, excluding the node: antibiotic-loaded bone cement 57

S1 Protocol 58

S1 – Search Strategy

CENTRAL was searched using the following exploded MeSH headings and keywords:

#1	MeSH descriptor: [Arthroplasty, Replacement, Hip] 3 tree(s) exploded
#2	(hip) (Word variations have been searched)
#3	(joint)
#4	#2 OR #3
#5	surger*
#6	replace*
#7	implant*
#8	arthroplast*
#9	prosthes*
#10	#5 OR #6 OR # #7 OR #8 OR #9
#11	#4 AND #10
#12	tha
#13	thr
#14	#1 OR #11 OR #12 OR #13
#15	MeSH descriptor: [Anti-Bacterial Agents] explode all trees
#16	MeSH descriptor: [Antibiotic Prophylaxis] explode all trees
#17	MeSH descriptor: [Aminoglycosides] explode all trees
#18	MeSH descriptor: [Vancomycin] explode all trees
#19	MeSH descriptor: [Cephalosporins] explode all trees
#20	MeSH descriptor: [Ciprofloxacin] explode all trees
#21	MeSH descriptor: [Ofloxacin] explode all trees
#22	MeSH descriptor: [Aztreonam] explode all trees
#23	MeSH descriptor: [Trimethoprim, Sulfamethoxazole Drug Combination] explode all trees
#24	MeSH descriptor: [Oxazolidinones] explode all trees
#25	#15 OR #16 OR #17 OR #18 OR #19 OR #20 OR #21 OR #22 OR #23 OR #24
#26	(4perations4ide*)
#27	cephalosporin*
#28	cefazolin
#29	cefepime
#30	cefuroxime
#31	ciprofloxacin
#32	vancomycin
#33	aztreonam*
#34	levaquin
#35	trimethoprim
#36	linezolid
#37	oxazolidinone*
#38	Ofloxacin*
#39	antibiotic*
#40	antibacterial*
#41	anti-bacterial*
#42	agent*
#43	#41 and #42
#44	#26 OR #27 OR #28 OR #29 OR #30 OR #31 OR #32 OR #33 OR #34 OR #35 OR #36 OR #37 OR #38 OR #39 OR #40
#45	anti
#46	bacterial
#47	agent*
#48	#45 AND #46 #47
#49	anti-bacterial*
#50	anti-bacterial
#51	agent*

#52 #50 AND #51
#53 #48 OR #52 OR #43
#54 #44 OR #53
#55 #54 OR #25
#56 #14 AND #55

Search String for Medline via PubMed
The search string includes terms relating to or describing the population and intervention and the database specific filters suggested by Cochrane for identifying randomized trials.

0. *Population*

THA[tw] OR THR[tw] OR Arthroplasty, Replacement, Hip[MeSH] **OR ((**Hip[tw] OR Joint[tw]) **AND** (Replace*[tw] OR prosthes*[tw] OR Implant*[tw] OR Arthroplast*[tw] OR Surger*[tw]))

2. Intervention

antibiotic*[tw] OR antibacterial*[tw] OR ("anti-bacterial"[tw] AND "agents"[tw]) OR "anti-bacterial agents"[tw] OR ("anti"[tw] AND "bacterial"[tw] AND "agents"[tw]) OR "anti bacterial agents"[tw]) OR 5perations5ide*[tw] OR cephalosporin*[tw] OR cefazolin[tw] OR cefepime[tw] OR cefuroxime[tw] OR ciprofloxacin[tw] OR vancomycin[tw] OR aztreonam*[tw] OR 5peratio[tw] OR trimethoprim[tw] OR linezolid[tw] OR oxazolidinone*[tw] OR ofloxacin*[tw] "Anti-Bacterial Agents"[MeSH Terms] OR "Anti-Bacterial Agents"[Pharmacological Action] OR "Antibiotic Prophylaxis"[MeSH Terms] OR "Aminoglycosides"[Mesh] OR "Vancomycin"[MeSH Terms] OR "Cephalosporins"[MeSH Terms] OR "Ciprofloxacin"[MeSH Terms] OR "Ofloxacin"[MeSH Terms] OR "Aztreonam"[MeSH Terms] OR "trimethoprim, sulfamethoxazole drug combination"[MeSH Terms] OR "Oxazolidinones"[MeSH Terms]

3. *RCT filter from Cochrane: **Sensitivity-maximizing version (2008 revision)***; **PubMed format (1)**

(randomized controlled trial[pt] OR controlled clinical trial[pt] OR randomized[tiab] OR placebo[tiab] OR drug therapy[sh] OR randomly[tiab] OR trial[tiab] OR groups[tiab] NOT (animals [mh] NOT humans [mh]))

4. #1 AND #2 AND # 3

Search String for Embase via Ovid

0. *Population*

tha.mp or thr.mp or exp total hip replacement/ or ((hip.mp or joint.mp) and (replace*.mp or prosthes*.mp or implant*.mp or 5perations5i*.mp or surger*.mp))

2. Intervention

antibiotic*.mp or antibiotic prophylaxis.mp or antibacterial*.mp oraminoglycosid*.mp or cephalosporin*.mp orcefazolin.mp or cefepime.mp or cefuroxime.mp or ciprofloxacin.mp or vancomycin.mp or aztreonam*.mp or 5peratio.mp or trimethoprim.mp or linezolid.mp or oxazolidinone*.mp or trimethoprim.mp or ofloxacin.mp or exp antibiotic prophylaxis/ or exp antibiotic agent/ or exp oxazolidinone derivative/ or exp trimethoprim sulfate/ or sulfamerazine plus trimethoprim/ or exp trimethoprim/ or exp sulfadoxine plus trimethoprim/ or exp rifampicin plus trimethoprim/ or exp trimethoprim derivative/ or exp vancomycin/ or exp gentamicin/ or exp clindamycin/ or exp aztreonam lysine/ or exp aztreonam/ or exp clavulanic acid/ or exp cefepime/ or exp ofloxacin/ or exp ciprofloxacin/ or exp cephalosporin derivative/ or exp cephalosporin/ exp aminoglycoside antibiotic agent/ or exp aminoglycoside/ exp linezolid/ or exp levofloxacin/ or exp cefepime/ or exp cefazolin/

3. *RCT filter, the sensitivity maximizing version, Ovid format (2)*

(Randomized controlled trial/ or Controlled clinical study/ or random$.ti,ab. Or randomization/ or intermethod comparison/ or placebo.ti,ab. Or (compare or compared or comparison).ti. or ((evaluated or evaluate or evaluating or assessed or assess) and (compare or compared or comparing or comparison)).ab. or (open adj label).ti,ab. Or ((double or single or doubly or singly) adj (blind or blinded or blindly)).ti,ab. Or double blind procedure/ or parallel group$1.ti,ab. Or (crossover or cross over).ti,ab. Or ((assign$ or match or matched or allocation) adj5 (alternate or group$1 or intervention$1 or patient$1 or subject$1 or participant$1)).ti,ab. Or (assigned or allocated).ti,ab. Or (controlled adj7 (study or design or trial)).ti,ab. Or (volunteer or volunteers).ti,ab. Or human experiment/ or trial.ti.) not (((random$ adj sampl$ adj7 ("cross section$" or questionnaire$1 or survey$ or database$1)).ti,ab. Not (comparative study/ or controlled study/ or randomi?ed controlled.ti,ab. Or randomly assigned.ti,ab.)) or (Cross-sectional study/ not (randomized controlled trial/ or controlled clinical study/ or controlled study/ or randomi?ed controlled.ti,ab. Or control group$1.ti,ab.)) or (((case adj control$) and random$) not randomi?ed controlled).ti,ab. Or (Systematic review not (trial or study)).ti. or (nonrandom$ not random$).ti,ab. Or "Random field$".ti,ab. Or (random cluster adj3 sampl$).ti,ab. Or ((review.ab. and review.pt.) not trial.ti.) or ("we searched".ab. and (review.ti. or review.pt.)) or "update review".ab. or (databases adj4 searched).ab. or ((rat or rats or mouse or mice or swine or porcine or murine or sheep or lambs or pigs or piglets or rabbit or rabbits or cat or cats or dog or dogs or cattle or bovine or monkey or monkeys or trout or marmoset$1).ti. and animal experiment/) or (Animal experiment/ not (human experiment/ or human/)))

4. #1 AND #2 AND # 3

The above MeSH terms and keywords were also used for, CINAHL and Web of Science. There were no restrictions on the basis of date or language of publication.

1. Lefebvre C ME, Glanville J. Box 6.4.a: Cochrane Highly Sensitive Search Strategy for identifying randomized trials in MEDLINE: sensitivity-maximizing version (2008 revision); PubMed format. In: Higgins J GS, editor. Cochrane Handbook for Systematic Reviews of Interventions Version 510 (updated March 2011) The Cochrane Collaboration, 2011.
2. Glanville J, Foxlee R, Wisniewski S, Noel-Storr A, Edwards M, Dooley G. Translating the Cochrane EMBASE RCT filter from the Ovid interface to Embase.com: a case study. (1471-1842 (Electronic)).
3. Carlsson AK, Lidgren L, Lindberg L. Prophylactic antibiotics against early and late deep infections after total hip replacements. Acta Orthop Scand. 1977;48(4):405-10.

Database: https://www.cochranelibrary.com/advanced-search (December 25, 2022) Search Strategy

ID	Search
#1	MeSH descriptor: [Arthroplasty, Replacement, Hip] 3 tree(s) exploded
#2	(hip) (Word variations have been searched)
#3	(joint)
#4	#2 OR #3
#5	surger*
#6	replace*
#7	implant*
#8	arthroplast*
#9	prosthes*
#10	#5 OR #6 OR # #7 OR #8 OR #9

#11	#4 AND #10
#12	tha
#13	thr
#14	#1 OR #11 OR #12 OR #13
#15	MeSH descriptor: [Anti-Bacterial Agents] explode all trees
#16	MeSH descriptor: [Antibiotic Prophylaxis] explode all trees
#17	MeSH descriptor: [Aminoglycosides] explode all trees
#18	MeSH descriptor: [Vancomycin] explode all trees
#19	MeSH descriptor: [Cephalosporins] explode all trees
#20	MeSH descriptor: [Ciprofloxacin] explode all trees
#21	MeSH descriptor: [Ofloxacin] explode all trees
#22	MeSH descriptor: [Aztreonam] explode all trees
#23	MeSH descriptor: [Trimethoprim, Sulfamethoxazole Drug Combination] explode all trees
#24	MeSH descriptor: [Oxazolidinones] explode all trees
#25	#15 OR #16 OR #17 OR #18 OR #19 OR #20 OR #21 OR #22 OR #23 OR #24
#26	(7perations7ide*)
#27	cephalosporin*
#28	cefazolin
#29	cefepime
#30	cefuroxime
#31	ciprofloxacin
#32	vancomycin
#33	aztreonam*
#34	levaquin
#35	trimethoprim
#36	linezolid
#37	oxazolidinone*
#38	Ofloxacin*
#39	antibiotic*
#40	antibacterial*
#41	anti-bacterial*
#42	agent*
#43	#41 nd #42

#44 #26 OR #27 OR #28 OR #29 OR #30 OR #31 OR #32 OR #33 OR #34 OR #35 OR #36 OR #37 OR #38 OR #39 OR #40

#45 anti

#46 bacterial

#47 agent*

#48 #45 AND #46 #47

#49 anti-bacterial*

#50 anti-bacterial

#51 agent*

#52 #50 AND #51

#53 #48 OR #52 OR #43

#54 #44 OR #53

#55 #54 OR #25

#56 #14 AND #55

S2 List of excluded trials

No.	Author	Year	Reference (extracted from covidence)	Reasons for exclusion
1	Chiu	1993	A prospective, randomized study of four regimes of antibiotic prophylaxis for hip fracture operations<br>Chiu, K. Y.; Ng, K. H.; Fung, B.; Lau, S. K.; Chow, S. P.<br>Journal of bone and joint surgery – 8perati volume 1993;75 Suppl 3():230<br>1993	Wrong patient population
2	Nct	2020	Intra-articular Vancomycin Powder in Knee and Hip Arthroplasty<br>Nct,<br>https://clinicaltrials.gov/show/NCT04399642 2020;():<br>2020	Ongoing trial
3	Wymenga	1991	Antibiotic use after cefuroxime prophylaxis in hip and knee joint replacement<br>Wymenga, A. B.; Hekster, Y. A.; Theeuwes, A.; Muytjens, H. L.; van Horn, J. R.; Slooff, T. J.<br>Clin Pharmacol Ther Aug 1991;50(2):215-20<br>1991 Aug	Preliminary results of included trial
4	Ritter	1989	Comparison of intraoperative versus 24 hour antibiotic prophylaxis in total joint replacement. A controlled prospective study<br>Ritter, M. A.; Campbell, E.; Keating, E. M.; Faris, P. M.<br>Orthop Rev Jun 1989;18(6):694-6<br>1989 Jun	Study population not analyzed according to type of implant received
5	Thierse	1978	[Experiences with Refobacin-Palacos with regard to deep late infections following hip-joint endoprosthesis surgery. A 4-years' study (author's transl)]<br>Thierse, L.<br>Z Orthop Ihre Grenzgeb 1978;116(6):847-52<br>1978	Wrong study design
6	Nadeem	2015	Antibiotic prophylaxis in hip surgery: A 8perations of two vs. three doses of cefuroxime<br>Nadeem, R. D.; Akhtar, M.; Cheema, O. I.; Hashmi, A. R.; Nadeem, M. J.; Nadeem, A.	Follow-up ≤ 90 days

			J Pak Med Assoc Nov 2015;65(11 Suppl 3):S136-41<br>2015 Nov	
7	Boyd	1973	A double-blind clinical trial of prophylactic antibiotics in hip fractures<br>Boyd, R. J.; Burke, J. F.; Colton, T.<br>J Bone Joint Surg Am Sep 1973;55(6):1251-8<br>1973 Sep	Study population not analyzed according to type of implant received
8	Unspecified	1985	Prevention of infection after joint surgery<br>Lancet Mar 23 1985;1(8430):694<br>1985 Mar 23	Wrong study design
9	Nct	2022	Effect of Single vs Multiple Prophylactic Antibiotic Doses on PJI Following Primary THA in Patients With a Fracture<br>Nct,<br>https://clinicaltrials.gov/show/NCT05530174 2022;():<br>2022	Ongoing Trial
10	Rasch	1993	Preoperative antibiotic prophylaxis with 9perations in total prothetic replacement on hip and knee. [German]<br>Rasch, F.; Schaferhoff, P.; Frank, D.; Biehl, G.<br>Medizinische Welt 1993;44(12)():754-756<br>1993	No full-text available for retrieval
11	Carlsson	1977	Prophylactic antibiotics against early and late infections after total hip replacement<br>Carlsson, A. S.<br>Acta Orthopaedica Scandinavica 1977;48():405-410<br>1977	Long-term results of included trial
12	Peel	2019	Multicentre 9perations double-blind placebo controlled trial of combination vancomycin and cefazolin surgical antibiotic prophylaxis: the Australian surgical antibiotic prophylaxis (ASAP) trial<br>Peel, T.; Astbury, S.; Cheng, A. C.; Paterson, D.; Buising, K.; Spelman, T.; Tran-Duy, A.; de Steiger, R. S.<br>BMJ Open Nov 3 2019;9(11):e033718<br>2019 Nov 3	Ongoing trial<br><br>Study published October in 2023 and mention in discussion.
13	Romaò	2016	Does an Antibiotic-Loaded Hydrogel Coating Reduce Early Post-Surgical Infection After Joint Arthroplasty?<br>Romanò, C. L.; Malizos, K.; Capuano, N.; Mezzoprete, R.; D'Arienzo, M.; Van Der Straeten, C.; Scarponi, S.; Drago, L.<br>J Bone Jt Infect 2016;1():34-41<br>2016	Wrong setting
14	Nct	2014	The Use of Ceftaroline as Surgical Prophylaxis in Surgery With Risk of MRSA Infection<br>Nct,<br>https://clinicaltrials.gov/show/NCT02307006 2014;():<br>2014	Trial never completed
15	Wall	1988	A comparison of teicoplanin and cefuroxime as prophylaxis for orthopaedic implant surgery: a preliminary report<br>Wall, R.; Klenerman, L.; McCullough, C.; Fyfe, I.<br>J Antimicrob Chemother Jan 1988;21 Suppl A():141-6<br>1988 Jan	Follow-up ≤ 90 days
16	Frajman	1991	[Prevention of postoperative infections with cefotiam (Pansporine) in orthopedic surgery]<br>Frajman, J. M.; Joubert-Collin, M.; Durgeat, S.; Duparc, J.<br>Agressologie 1991;32(10 Spec No):467-70<br>1991	Study population not analyzed according to type of implant received
17	Scales	1972	The influence of antibiotic therapy on wound inflammation and sepsis associated with orthopaedic implants. A long-term clinical survey<br>Scales, J. T.; Towers, A. G.; Roantree, B. M.<br>Acta Orthop Scand 1972;43(2):85-100<br>1972	Wrong study design
18	Kanellakopoulou	2009	Efficacy of teicoplanin for the prevention of surgical site infections after total hip or knee arthroplasty: a prospective, open-label study	Wrong study design

			Kanellakopoulou, K.; Papadopoulos, A.; Varvaroussis, D.; Varvaroussis, A.; Giamarellos-Bourboulis, E. J.; Pagonas, A.; Stergiou, A.; Papadelis, P.; Nikolaidis, V.; Giamarellou, H.<br>Int J Antimicrob Agents May 2009;33(5):437-40<br>2009 May	
19	Periti	1999	Comparative multicenter trial of teicoplanin versus cefazolin for antimicrobial prophylaxis in prosthetic joint implant surgery. Italian Study Group for Antimicrobial Prophylaxis in Orthopedic Surgery<br>Periti, P.; Stringa, G.; Mini, E.<br>Eur J Clin Microbiol Infect Dis Feb 1999;18(2):113-9<br>1999 Feb	Study population not analyzed according to type of implant received
20	Freick	1984	Antibiotic prophylaxis with mezlocillin in total hip replacement surgery<br>Freick, H.; Opferkuch, W.; Müller-Thurmann, M.; Piontek, R.<br>Die Medizinische Welt 1984;35():938-942<br>1984	No full-text available for retrieval
21	Vainiompaa	1988	Cefamandole and isoxazolyl penicillins in antibiotic prophylaxis of patients undergoing total hip or knee-joint arthroplasty<br>Vainionpaa, S.; Wilppula, E.; Lalla, M.; Renkonen, O. V.; Rokkanen, P.<br>Archives of Orthopaedic and Traumatic Surgery 1988;107(4)():228-230<br>1988	Wrong study design
22	Wilson	1973	The problem of infection in endoprosthetic surgery of the hip joint<br>Wilson, P. D., Jr.; Salvati, E. A.; Aglietti, P.; Kutner, L. J.<br>Clin Orthop Relat Res Oct 1973;(96):213-21<br>1973 Oct	Wrong study design
23	Davis	1987	Antimicrobial prophylaxis for arthroplasty: a comparative study of cefonicid and cefazolin<br>Davis, W. A.; Kane, J. G.<br>Orthopedics Oct 1987;10(10):1405-9<br>1987 Oct	Study population not analyzed according to type of implant received
24				
25	De Lachica	2022	Decrease in acute periprosthetic joint infections incidence with vancomycin-loaded calcium sulfate beads in patients with non-modifiable risk factors. A randomized clinical trial<br>de Lachica, J. C. V.; Reyes, S. S. S.; Ureña, J. A. P.; Fragoso, M. A. R.<br>J isakos Dec 2022;7(6):201-205<br>2022 Dec	Wrong study design
26	Nct	2019	Prophylaxis of Periprosthetic Joint Infections With Calcium Sulfate Beads in Patients With Non-modifiable Risk Factors<br>Nct,<br>https://clinicaltrials.gov/show/NCT03976466 2019;():<br>2019	Wrong patient population
27	Mwaura	2019	Implant stability and migration in an antibiotic coated cementless primary total hip arthroplasty: a randomized controlled trial<br>Mwaura, B.; Karlakki, S.; Whittaker, J. P.; Graham, N.; Gregson, P.; Dhawan, R.; Wilkinson, M.; Jones, R.; Hunt, A.<br>American academy of orthopaedic surgeons 2019;():<br>2019	No full-text available for retrieval
28	Feil	1990	[Bioresorbable collagen-gentamicin compound as local antibiotic therapy]<br>Feil, J.; Bohnet, S.; Neugebauer, R.; Rübenacker, S.<br>Aktuelle Probl Chir Orthop 1990;34():94-103<br>1990	Wrong study design
29	Buckley	1990	Perioperative cefazolin prophylaxis in hip fracture surgery<br>Buckley, R.; Hughes, G. N. F.; Snodgrass, T.; Huchcroft, S. A.<br>Canadian Journal of Surgery 1990;33(2)():122-125<br>1990	Wrong patient population
30	Riska	1980	Are antibiotics necessary in the prevention of infection in total hip replacement?<br>Riska, E. B.<br>Ann Chir Gynaecol 1980;69(3):122-4<br>1980	Wrong study design

31	McQueen	1987	A comparison of systemic cefuroxime and cefuroxime loaded bone cement in the prevention of early infection after total joint replacement<br>McQueen, M.; Littlejohn, A.; Hughes, S. P.<br>Int Orthop 1987;11(3):241-3<br>1987	Study population not analyzed according to type of implant received
32	Dinges	1991	Antibiotic prophylaxis in total hip endoprosthesis. [German]<br>Dinges, H.; Thabe, H.; Schassan, H. H.<br>Aktuelle Rheumatologie 1991;16(3)():108-111<br>1991	Wrong study design
33	Hughes	1993	Pevention of infection in orthopaedic surgery<br>Hughes, S.<br>Prescribers' Journal 1993;33(5)():191-195<br>1993	No full-text available for retrieval
34	Tyllianakis	2004	Prospective comparative study of Fusidic acid, Vancomycin and Cefuroxime, in prophylactic use in T.H.R and T.K.R. early results<br>Tyllianakis, M.; Karageorgos, A.; Marangos, M.; Lambiris, E.<br>The journal of bone and joint surgery (proceedings) 2004;86-B(SUPP_III):305-30d<br>2004	Preliminary results of trial, later excluded due to dosage regimens compared in trial assessed identical
35	Josefsson	1993	Prophylaxis with systematic antibiotics versus gentamicin bone cement in total hip arthroplasty. A ten-year survey of 1,688 hips<br>Josefsson, G.; Kolmert, L.<br>Clin Orthop Relat Res Jul 1993;(292):210-4<br>1993 Jul	Long-term results of included trial
36	Pavel	1977	Prophylactic antibiotics in elective orthopedic surgery: a prospective study of 1,591 cases<br>Pavel, A.; Smith, R. L.; Ballard, A.; Larson, I. J.<br>South Med J Oct 1977;70 Suppl 1():50-5<br>1977 Oct	Study population not analyzed according to type of implant received
37	Kaukonen	1995	One dose cefuroxime prophylaxis in hip fracture surgery<br>Kaukonen, J. P.; Kemppainen, E.; Mäkijärvi, J.; Tuominen, T.<br>Ann Chir Gynaecol 1995;84(4):417-9<br>1995	Wrong patient population
38	Nct	2022	Linezolid or Vancomycin Surgical Site Infection Prophylaxis<br>Nct,<br>https://clinicaltrials.gov/show/NCT05571722 2022;():<br>2022	Ongoing trial
39	Pressato	2017	DAC® gel a hyaluronan based hydrogel antibiotic-loaded against biofilm formation: new clinical perspective in the prevention of periprosthetic joint infection<br>Pressato, D.; Bellini, D.; Sacchetta, A.; Meraner, J.; Meani, E.; Romano, C. L.<br>Journal of Applied Biomaterials and Functional Materials 2017;15(3):e283-<br>2017	No full-text available for retrieval
40	Nct	2021	Topical Vancomycin for Infection Prophylaxis in TJA<br>Nct,<br>https://clinicaltrials.gov/show/NCT04993027 2021;():<br>2021	Ongoing trial
41	Elsaqa	2022	One day versus three days' antibiotic prophylaxis in joint arthroplasty. A prospective randomized controlled trial<br>Elsaqa,Mahmoud; Karim,Mahmoud A.; Ebeid,Walid; Youness,Mohamed<br>Journal of Musculoskeletal Surgery and Research ;6():	Study population not analyzed according to type of implant received
42	Boittiaux	1986	[Prevention of infections during total hip arthroplasties performed under laminar air flow. Value of prophylactic antibiotic therapy using cephalosporins]<br>Boittiaux, P.; Krivosic-Horber, R.; Duquennoy, A.; Verlaine, A.; Liebaert, F.<br>Cah Anesthesiol Oct 1986;34(6):491-4<br>1986 Oct	Study population not analyzed according to type of implant received
43	Li	2014	Rifampicin combined with levofloxacin for preventing infection after total hip arthroplasty<br>Li, C.; Shang, X. F.; Cao, X. F.; Gan, Z. Y.; Dou, Z. Y.	Not a language we speak

			Chinese Journal of Tissue Engineering Research 2014;18(48):7714-7718<br>2014	
44	Nct	2019	Vancomycin Powder and Dilute Povidone Iodine Lavage for Infection Prophylaxis in High Risk Total Joint Arthroplasty<br>Nct,<br>https://clinicaltrials.gov/show/NCT04075526 2019;():<br>2019	Ongoing trial
45	Pavel	1974	Prophylactic antibiotics in clean orthopaedic surgery<br>Pavel, A.; Smith, R. L.; Ballard, A.; Larsen, I. J.<br>Journal of bone and joint surgery. American volume 1974;56(4):777-782<br>1974	Study population not analyzed according to type of implant received
46	Demartines	1989	[Total hip prosthesis with or without preventive use of antibiotics]<br>Demartines, N.; Steiner, W.; Noesberger, B.<br>Helv Chir Acta Jun 1989;56(1-2):85-9<br>1989 Jun	Wrong study design
47	Bahebeck	2009	Implant orthopaedic surgery in HIV asymptomatic carriers: management and early outcome<br>Bahebeck, J.; Eone, D. H.; Nonga, B. N.; Kingue, T. N.; Sosso, M.<br>Injury Nov 2009;40(11):1147-50<br>2009 Nov	Study population not analyzed according to type of implant received
48	Soave	1986	Comparison of ceforanide and cephalothin prophylaxis in patients undergoing total joint arthroplasty<br>Soave, R.; Hirsch, J. C.; Salvati, E. A.; Brause, B. D.; Roberts, R. B.<br>Orthopedics Dec 1986;9(12):1657-60<br>1986 Dec	Study population not analyzed according to type of implant received
49	Wollinsky	1996	Effect of antibiotic prophylaxis with cefuroxime on bacteriologic quality of intra- and postoperatively processed wound blood in hip joint arthroplasty<br>Wollinsky, K. H.; Buchele, M.; Oethinger, M.; Kluger, P.; Mehrkens, H. H.; Marre, R.; Puhl, W.<br>Infusionstherapie und Transfusionsmedizin September 1996;23(4-5)():180-186<br>1996 September	Wrong setting
50	Mollan	1992	Teicoplanin vs cephamandole for antimicrobial prophylaxis in prosthetic joint implant surgery: (preliminary results)<br>Mollan, R. A.; Haddock, M.; Webb, C. H.<br>Eur J Surg Suppl 1992;(567):19-21<br>1992	Study population not analyzed according to type of implant received
51	Periti	1989	Ceftriaxone as short-term antimicrobial chemoprophylaxis in orthopedic surgery: A 1-year multicenter follow-up. Preliminary results of a controlled multicentre study<br>Periti, P.; Jacchia, E.<br>European Surgical Research 1989;21(SUPPL. 1)():25-32<br>1989	Study population not analyzed according to type of implant received
52	Bryant	1982	Chemoprophylaxis in cardiac and orthopedic surgery: Comparison of cephalothin and cephapirin<br>Bryant, R. E.; Hartstein, A. I.; Starr, A.; Beals, R. K.<br>Southern Medical Journal 1982;75(9)():1057-1062<br>1982	Wrong study design
53	Visuri	1976	A comparison of dicloxacillin and ampicillin in the antibiotic prophylaxis of total hip replacement<br>Visuri, T.; Antila, P.; Laurent, L. E.<br>Ann Chir Gynaecol Suppl 1976;65(1):58-61<br>1976	Wrong study design
54	Wollinsky	1997	Autotransfusion – bacterial contamination during hip arthroplasty and efficacy of cefuroxime prophylaxis. A randomized controlled study of 40 patients<br>Wollinsky, K. H.; Oethinger, M.; Buchele, M.; Kluger, P.; Puhl, W.; Mehrkens, H. H.<br>Acta Orthopaedica Scandinavica 1997;68(3)():225-230<br>1997	Follow-up ≤ 90 days

55	Jones	1987	Single-dose cephalosporin prophylaxis of 929 surgical procedures in a prepaid group practice: a prospective, randomized comparison of cefoperazone and cefotaxime<br>Jones, R. N.; Wojeski, W. V.<br>Diagn Microbiol Infect Dis Apr 1987;6(4):323-34<br>1987 Apr	Follow-up ≤ 90 days
56	Isrctn	2001	Randomised trial of extended antibiotic prophylaxis for fracture fixation and joint replacement: clinical benefits versus ecological risks<br>Isrctn,<br>https://trialsearch.who.int/Trial2.aspx?TrialID=ISRCTN75423827 2001;():<br>2001	Trial never completed
57	Nct	2010	Daptomycin Use for Antimicrobial Prophylaxis in Methicillin Resistant Staphylococcus Aureus (MRSA) Colonized Adult Patients Undergoing Primary Elective Hip, Knee, or Shoulder Arthroplasty<br>Nct,<br>https://clinicaltrials.gov/show/NCT01196169 2010;():<br>2010	Trial never completed
58	Carlsson	1977	Prophylactic antibiotics against early and late deep infections after total hip replacements<br>Carlsson, A. S.; Lidgren, L.; Lindberg, L.<br>Acta Orthopaedica Scandinavica 1977;48(4)():405-410<br>1977	Duplicate
59	Rosenfeld	1981	Chemoprophylaxis with cefoxitin and cephalothin in orthopedic surgery: a comparison<br>Rosenfeld, M. B.; Campos, J.; Ratzan, K. R.; Uredo, I.<br>Antimicrob Agents Chemother May 1981;19(5):826-30<br>1981 May	Follow-up ≤ 90 days
60	Nct	2022	Effect of Single vs Multiple Prophylactic Antibiotic Doses on PJI Following Primary THA in Patients With OA<br>Nct,<br>https://clinicaltrials.gov/show/NCT05530551 2022;():<br>2022	Ongoing trial
61	Miller	1986	Antibiotics in open fractures: a prospective 13perations, double- blind study of wound infection<br>Miller, S. D.; Bray, R. C.; Hughes, G. N. F.<br>Journal of bone and joint surgery – 13perati volume 1986;68(5):850<br>1986	Wrong study design
62	Nct	2011	Efficacy of Local Powder Prophylactics<br>Nct,<br>https://clinicaltrials.gov/show/NCT01372371 2011;():<br>2011	Wrong patient population
63	Burnett	1980	Prophylactic antibiotics in hip fractures. A double-blind, prospective study<br>Burnett, J. W.; Gustilo, R. B.; Williams, D. N.; Kind, A. C.<br>J Bone Joint Surg Am Apr 1980;62(3):457-62<br>1980 Apr	Wrong patient population
64	Doyon	1987	Long-term results of prophylactic cefazolin versus placebo in total hip replacement<br>Doyon, F.; Evrard, J.; Mazas, F.; Hill, C.<br>Lancet Apr 11 1987;1(8537):860<br>1987 Apr 11	Wrong study design
65	Aebi	1989	[Prevention of infection in elective orthopedic interventions with special reference to alloplastic joint replacement]<br>Aebi, B.; Gerber, C.; Ganz, R.<br>Helv Chir Acta Aug 1989;56(3):387-97<br>1989 Aug	Wrong study design
66	Naessens	1983	Prophylactic antibiotics in surgery<br>Naessens, A.; Lauwers, S.<br>Acta Anaesthesiol Belg Sep 1983;34(3):163-71<br>1983 Sep	Wrong study design

67	Karachalios	1987	Single-dose prophylaxis of ceftriaxone versus standard dosage of cefotaxime in the prophylaxis of bacterial complications in orthopedic surgery<br>Karachalios, T.; Lyritis, G.; Hatzopoulos, E.; Sapkas, G.<br>Chemioterapia Jun 1987;6(2 Suppl):573-5<br>1987 Jun	Wrong patient population
68	Periti	1992	Teicoplanin–its role as systemic therapy of burn infections and as prophylaxis for orthopaedic surgery. Italian Study Groups for Antimicrobial Prophylaxis in Orthopaedic Surgery and Burns<br>Periti, P.; Stringa, G.; Donati, L.; Mazzei, T.; Mini, E.; Novelli, A.<br>Eur J Surg Suppl 1992;(567):3-8<br>1992	Study population not analyzed according to type of implant received
69	Berglund	1981	A comparison between flucloxacillin and dicloxacillin in the antibiotic prophylaxis of total hip replacement<br>Berglund, B.; Laurent, L. E.; Ojajarvi, J.; Soini, J.<br>Acta Orthopaedica Scandinavica 1981;52(4)():465<br>1981	No full-text available for retrieval
70	Buchholz	1972	[Infection prevention and surgical management of deep insidious infection in total endoprosthesis]<br>Buchholz, H. W.; Gartmann, H. D.<br>Chirurg Oct 1972;43(10):446-53<br>1972 Oct	Wrong study design
71	Winter	1987	Flucloxacillin and ceftriaxone in the perioperative prophylaxis of patients undergoing prosthetic hip and knee surgery by a prospective randomized trial<br>Winter, M.; Ungemach, J.; Glicksman, H.<br>Chemioterapia Jun 1987;6(2 Suppl):577<br>1987 Jun	Study population not analyzed according to type of implant received
72	Nct	2021	Evaluation of Emerging New Treatments for Infection Prevention in Total Joint Replacement<br>Nct,<br>https://clinicaltrials.gov/show/NCT05084378 2021;():<br>2021	Ongoing trial
73	Nct	2020	Antibiotic Prophylaxis in High-Risk Arthroplasty Patients<br>Nct,<br>https://clinicaltrials.gov/show/NCT04297592 2020;():<br>2020	Ongoing trial
74	Josefsson	1990	Prophylaxis with systemic antibiotics versus gentamicin bone cement in total hip arthroplasty. A five-year survey of 1688 hips<br>Josefsson, G.; Gudmundsson, G.; Kolmert, L.; Wijkstrom, S.<br>Clinical Orthopaedics and Related Research 1990;253():173-178<br>1990	Long-term results of included trial
75	Bryan	1988	Cefazolin versus cefamandole for prophylaxis during total joint arthroplasty<br>Bryan, C. S.; Morgan, S. L.; Caton, R. J.; Lunceford, E. M., Jr.<br>Clin Orthop Relat Res Mar 1988;(228):117-22<br>1988 Mar	Study population not analyzed according to type of implant received
76	Nelson	1983	One day versus seven days of preventive antibiotic therapy in orthopedic surgery<br>Nelson, C. L.; Green, T. G.; Porter, R. A.; Warren, R. D.<br>Clin Orthop Relat Res Jun 1983;(176):258-63<br>1983 Jun	Wrong study design (Quasi-RCT)
77	Evrard	1988	Two-day cefamandole versus five-day cephazolin prophylaxis in 965 total hip replacements. Report of a multicentre double blind 14perations trial<br>Evrard, J.; Doyon, F.; Acar, J. F.; Salord, J. C.; Mazas, F.; Flamant, R.<br>Int Orthop 1988;12(1):69-73<br>1988	Dosage regimens compared in the studies were considered identical
78	Tyllianakis	2010	Antibiotic prophylaxis in primary hip and knee arthroplasty. Comparison between cefuroxime and two specific antistaphylococcal agents<br>Tyllianakis, M. E.; Karageorgos, A. C.; Marangos, M. N.; Saridis, A. G.; Lambiris, E. E.<br>Journal of Arthroplasty October 2010;25(7)():1078-1082<br>2010 October	Dosage regimens compared in the studies were considered identical

79	DeBenedictis	1984	A double-blind study comparing cefonicid with cefazolin as prophylaxis in patients undergoing total hip or knee replacement<br>DeBenedictis, K. J.; Rowan, N. M.; Boyer, B. L.<br>Rev Infect Dis Nov-Dec 1984;6 Suppl 4():S901-4<br>1984 Nov-Dec	Dosage regimens compared in the studies were considered identical
80	Schulitz	1980	The prophylactic use of antibiotics in alloarthroplasty of the hip joint for coxarthrosis<br>Schulitz, K. P.; Winkelmann, W.; Schoening, B.<br>Arch Orthop Trauma Surg (1978) 1980;96(2):79-82<br>1980	Unspecified time-point for outcome assessment within the 2-year follow-up
81	Ritter	1983	Cephalosporin prophylaxis for total hip replacement<br>Ritter, M. A.; Conway, M. F.; Stringer, E. A.; Williams, J. G.<br>Orthopedics Jul 1 1983;6(7):850-5<br>1983 Jul 1	Unspecified time-point for outcome assessment within the 2-year follow-up

S3 Table. Characteristics of the included trials

Note: [Ordered by year of publication] The information hereby presented has been copied from the original article or adapted.

Ericson 1973

Methods RCT, parallel group, 2 centers in Sweden, the orthopaedic clinic in Lund or Malmö. 6 month follow-up, however Carlsson et al 1977 follow-up of the study for 1 year to 2.5 years (3)

Participants
Inclusion Criteria: Three types of 15perations were included (1) total arthroplasty of the hip with the Charnley prosthesis (2) arthroplasty with the Moore endoposthesis and (3) pertrochanteric or subtrochanteris femoral fractures fixed with a Thornton nail and plate.

171 (102 women, 69 men) were randomly assigned.
59 patients lost to investigation (32 in placebo group, 27 in cloxacillin group)

Comobidities Not stated

Interventions
Intervention 1 [Multiple-dose > 1 day]: 14 days of Cloxacillin 1g IM administered one hour prior to surgery + x 3 times for 1 day, followed by peroral tablets 0.5g x 4 until 14th day after operation + probenecid (n= 60)

Intervention 2 [placebo]: placebo, given the same way as intervention 1 + probenecid (n=58)

Outcomes Surgical site infections at 180 days after surgery

Outcome definitions Clinical signs and positive culture.
Surgical site infection: If the patient showed any clinical signs and if a culture on one test gave growth of either potentially pathogenic bacteria or doubtful cases on more than one culture. Moreover, infections was diagnosed when it was so strongly suspected that treatment was started with a known antibiotic

Results Intervention 1 [Multiple-dose > 1 day]: SSI = 0 , Intervention 2 [Placebo]: SSI = 10.

Pollard 1979

Methods RCT, parallel group, 3 operating theaters in England, Middlesex Hospital, King Edwards VII's Hospital for Officers and the Orthopaedic theatre of the Central Middlesex Hospital. 12-month follow-up.

Participants

Inclusion criteria: all patients receiving total hip replacement

Exclusion criteria: history of hypersensitivity to cephalosporins or penicillins, or with an appreciable degree of renal impairment
Indications for surgery: Osteoarthritis 89%, rheumatoid arthritis 8%, Fracture sequelae 3%, avascular necrosis <1%

310 total hip replacements were performed on 297 patients in the trial, as there were 13 bilateral cases. Seven patients were excluded (5 died within first 12 months, 2 lost to follow-up). 290 patients who underwent 303 total hip replacements. (178 performed on women, 125 on men).
Fifty-three of the patients were aged between 50 and 59 years, 133 between 60 and 69 years, 103 between 70-79 years and the remaining 14 under 50 years.
Mean age 64 years
Females constituted 59%

Comorbidities secondary or subsequent procedures 9%

Interventions

Intervention 1[Multiple-dose ≤ 24 hours]: 1 day of cephalonidine: 1g iv when anaesthesia was induced + 1g IM, 6 and 12 hours later (n=146)

Intervention 2 [Multiple-dose > 1 day]: 14 days of flucloxacillin: 500mg im with the premedication 1 hour before surgery + 500mg x 4 for 14 days, the first 24 hours im but subsequently it was taken by mouth (n=157)

Outcomes surgical site infections and prosthetic joint infections within 12 months after surgery

Outcome definitions

Sugical site infections

Superficial infection: Purulent discharge, with or without pyrexia.

Deep infection: pain, fever, redness with discharge containing pathogenic organism, elevated ESR, progressive radiographic resorption of bone stock.

Superficial infections were considered to be minor when there was a purulent discharge without pyrexia, moderate when there was a discharge accompanied by pyrexia, and severe when there was a major wound dehiscence.

Deep infection was considered to be either early or late-early when it occurred before six months and late thereafter.
Early infection was diagnosed by the presence of pain, fever, redness of the wound, and a discharge containing pathogenic organisms or many polymorphonuclear leucocytes.

Late infection was diagnosed by the presence of two or more of the following criteria: pain in the hip; a discharging sinus; isolation of pathogenic organisms from a sinus or by direct aspiration; isolation of material from which no organisms could be cultured and which contained many polymorphonuclear leucocytes; an erythrocyte sedimentation rate (ESR) that was raised above the preoperative level by 30 mm or more in the first hour; or radiological evidence of infection such as periosteal reaction, bone reabsorption, or irregular reabsorption of the calcar.

Results Intervention 1 [Multiple-dose ≤ 24 hours]: SAE = 2, SSI = 4, PJI = 2, Intervention 2 [Multiple-dose > 1 day]: SAE = 2, SSI = 5, PJI = 2.

Hill 1981

Methods Randomised double-blind placebo-controlled trial involving 9 centers in France, 2-year follow-up

Participants

Inclusion criteria: all patients undergoing hip prosthetic surgery
96% of patients were followed up for at least 6 months, 91% for one year and 80% for two years. 1 patient died during the operation, and 3 patients died of septic complications one month, two months, and three years after surgery.

Exclusion criteria: malignant hip tumors, or antibiotic treatment indicated for other reasons, or allergy to cephalosporins

2137 total hip replacements performed on 2097 patients (40 bilateral cases), 42% males and average age 64.5 years. For 169 patients (99 in the placebo group and 70 in the cefazolin group) the treatment code was broken before the end of the five days of treatment.

Primary diagnosis: osteoarthritis 85%, fracture 7%, rheumatoid arthritis 4%, failed osteotomi/hemiarthriplasty 4%, congenital dislocation 1%, ankylosing spondylitis 1%

Comobidities Previous hip surgery 11.2%, Intra-articular infiltration 0.9%, Diabetes Mellitus 2.6%, Obesity 18%, Alcoholism and cirrhosis 4.4%, Remote infection (urinary, dental etc.) 5.5%, Septic bone and joint 0.2%, Unexplained raised erythrocty sedimentations rate 1.4%, Corticosteroid therapy 2.7%, Antibiotic therapy 0.5%, Other treatments 22.3%, Other high risk for sepsis 2.2%, In hospital >10 days before surgery 5.6%

Interventions

Cefazolin (or placebo) was given at 1g every 6 hours for 5 days. The first injection was given at the time of induction of anesthesia.

Intervention 1 [Multiple-dose > 1 day]: 5 days of Cefazolin iv/im: 1g x 4 (1070)

Intervention 2 [Placebo]: 5 days of Placebo iv/im: x 4 (1067)

Outcomes SSI, PJI, and Serious Infections 90 days and 365 days after surgery

Outcome definitions PJI: Patients were recalled six months, twelve months and two years after operation. The condition of the hip was evaluated clinically, radiologically, and biologically (erythrocyte sedimentation rate). Hip infection was defined asa clinical infection in the hip (abscess, septicaemia, or lethal infection).

Serious infections: defined as septic complications including urinary, pulmonary, and digestive infections.

Results within 365 days, Intervention 1 [Multiple-dose > 1 day]: SAE = 84, SSI = 5, PJI = 5, serious infections = 79, Intervention 2 [Placebo]: SAE = 154, SSI = 27, PJI = 27, serious infections = 127 and within 90 days, Intervention 1 [Multiple-dose > 1 day]: SAE = 5, SSI = 5, PJI = 5, Intervention 2 [Placebo]: SAE = 16, SSI = 16, PJI = 16.

Josefson 1981

Methods RCT, parallel group, 9 orthopaedic centers in Sweden. 2-year follow-up.

Participants

Inclusion criteria: total hip arthroplasties

1,685 total hip arthroplasties were performed on 1,596 patients. During the investigation 50 patients died from diseases unrelated to the hip condition and 2 patients did not participate in follow-up.

Mean age 69

Female patients constituted 51%

Diagnosis: osteoarthritis 85%, fracture 7%, rheumatoid arthritis 4%, failed osteotomi/hemiarthroplasty 2%, congenital dislocation 1%, ankylosing spondylitis 1%

Comobidities Not stated.

Interventions

Intervention 1 [Multiple-dose > 1 day]: 7-14 days of Cloxacillin/cephalexine/ dicloxacillin/Phenoxymethylpenicillin, the first dose was given intramuscularly or intravenously one to 24 hours before the start of the operation hereafter iv/im/po: 0.5-1g x3-4 (n=812)

Intervention 2 [Antibiotic cement]: Gentamicin enriched bone cement (Palacos cum gentamicin©): 0.5g (n=821). To each 40-g packet of cement powder, gentamicin sulphate corresponding to 0.5 g of gentamicin base was added.

Outcomes SSI within 90 days, PJI within 365 days

Outcome definitions

Superficial: abnormal redness of wound, presence of secretion and firm diagnosis. Deep: pain, elevated erythrocyte sedimentation rate, a progressive radiographic resorption of bone stock

Superficial surgical site infection: abnormal redness of the wound, presence of secretion, and the fact that the diagnosis had been so firm that antibiotic treatment had been instituted.

Deep infection: A diagnosis of deep infection was based on the following three criteria: pain, elevated erythrocyte sedimentation rate (more than 35 mm per hour) and progressive radiographic resorption of bone stock. Four of the participating clinics had no facilities for the advanced bacteriologic investigations (e.g., anaerobic culture) necessary for a reliable bacteriologic diagnosis in loosened THA. A positive bacterial finding in the revised cases, therefore, could not be set as an absolute criterion for deep infection.

Results within 365 days, Intervention 1 [Multiple-dose > 1 day]: SAE = 10, SSI =59, PJI = 10, Intervention 2 [Antibiotic cement]: SAE = 2, SSI = 73, PJI = 2 and within 90 days Intervention 1 [Multiple-dose > 1 day]: SSI = 49, Intervention 2 [Antibiotic cement]: SSI = 71

Gunst 1984

Methods RCT, parallel group, single center Nantes, France. 12-month follow up.

Participants

Inclusion criteria: all patients undergoing hip prosthetic surgery

Exclusion criteria: previous surgery at hip level

A randomized study was performed in 93 total hip arthroplasties in 84 patients.

Mean age 64.5

Female patiens constituted 55%

Comobidities diabetes 3%, corticosteroid use 5%, immunosuppressed 1%, Other (alcoholism, obesity, kidney insufficiency, neoplasia) 17%

Interventions

Intervention 1 [Multiple-dose ≤ 24 hours]: Cefamandole iv: 1.5g with anaesthetic induction + 1.5g every 4 hours for 24h postoperatively (n=46)

Intervention 2[Placebo]: placebo/no antibiotic (n=47)

Cement without antibiotic

Outcomes Prosthetic joint infection within 90 days and 365 days after surgery

Outcome definitions

Clinical signs and positive culture

Patients were evaluated clinically and radiologically systematically at 6 weeks, 3 months, and 12 months postoperatively.

Serious complications defined as infections at the level of the prosthesis as a new surgical approach to the joint.

Early infections defined by the rapid appearance of infectious phenomena at the level of the prosthesis and reoperation within 1 month or delayed/late onset (longer than 1 month) of signs of deep infection, pain corresponding to the prosthesis and radiological signs of loosening.

Results Intervention 1 [Multiple-dose ≤ 24 hours]: SAE = 1, SSI = 1, PJI = 1, Intervention 2 [Placebo]: SAE = 8, SSI = 8, PJI = 8 and within 90 days Intervention 1 [Multiple-dose ≤ 24 hours]: SAE = 1, SSI = 1, PJI = 1, Intervention 2 [Placebo]: SAE = 5, SSI = 5, PJI = 5.

Centulio 1988

Methods RCT, parallel group, single-center, Italy. 18-month follow-up

Participants

Inclusion criteria: all total hip arthroplasties

Exclusion criteria: severe renal insufficiency (creatinine clearance < 10 ml/min), or an infectious disease before surgery, or administration of antibiotic 72 hours before surgery, or known history of hypersensitivity to beta-lactam antibiotics.

149 cementless total hip replacement implants were randomized

Mean age 63.2

64% were female patients.

Primary diagnosis: osteoarthritis 69%, fracture 9%, necrosis 4%, pseudoarthrosis collum femoris 2%, loose or infected prosthesis (reoperation) 16%

Comorbidities Revisions 16%

Interventions

Intervention 1[Multiple-dose > 1 day]: 3 days of ceftriaxone iv: 2 g 1-2 hours before surgery hereafter 2 g i.v. every 24 hours (n=81, primary THA n=69)

Intervention 2 [Single-dose]:: Single dose ceftriaxone 2 g iv 1-2 hours before surgery (n=68, primary THA n=56)

An anticoagulant therapy with calciheparin (0.5 cc x 2) was routinely treated in all patients for at least not 20 days.

Outcomes surgical site infections, prosthetic joint infections within 1 year after THA

Outcome definitions Infections described as either superficial or deep, no further elaboration apart from description of pathogen in case of culture. All patients included in the study were controlled with new hospitalizations or with periodic outpatient visits every 3 months in order to be able to exclude late infections.

Results Intervention 1 [Multiple-dose> 1 day]: SAE = 1, SSI = 1, PJI = 1, Intervention 2 [Single-dose]: SAE = 0, SSI = 1, PJI = 0.

McQueen 1990

Methods A controlled prospective, single blind randomized trial was performed in two centers, Scotland. 2-year follow-up.

Participants

Inclusion criteria: all patients receiving total hip or knee replacement
Exclusion criteria: not specified

378 patients undergoing 405 operations were entered into the trial.
There were 190 patients in each group receiving total hip arthroplasties, with 11 total knees in the intravenous group and 14 in the bone cement group.

The mean age of patients was 67 years.
Female to male ratio 2:1.
The principal diagnosis in both groups was osteoarthritis.

Comorbidities Not stated.

Interventions

Intervention 1 [Multiple-dose ≤ 24 hours]: 1.5 g of cefuroxime was administered intravenously at induction of anaesthesia, followed by two doses of 750 mg intramuscularly at 6 and 12 hours after operation (n=190)

Intervention 2 [Antibiotic cement]: 1.5 g of cefuroxime powder was mixed by the surgeon in the operating room with each pack of CMW type 1 cement powder. Barium sulphate was added simultaneously as a marker. The liquid polymer was added, and the operation continued in the usual way (n=190)

Outcomes surgical site infections, prosthetic joint infections within 90 and 365 days from surgery

Outcome definitions Superficial infection: Infection superficial to the deep fascia with positive or negative bacteriological cultures and no delay in wound healing

Deep infection: extending deep to the deep fascia, with persistent wound discharge or joint pain, positive or negative cultures from deep tissues and delay in wound healing.

Diagnosed by the presence of two or more of the following: Pain in or around joint, at rest or on movement erythrocyte sedimentation rate> 30 mm/hour above preoperative level
Pathogenic organisms from joint aspirates
Radiological evidence of infection, such as periosteal reaction or bone resorption
A persistent sinus in communication with the joint

Furthermore, classified as early (within 3 months) or late (3 months to 2 years).

Results Intervention 1 [Multiple-dose ≤ 24 hours]: SAE = 1, SSI = 9, PJI = 1, Intervention 2 [Antibiotic cement]: SAE = 2, SSI = 16, PJI = 2 and within 90 days Intervention 1 [Multiple-dose ≤ 24 hours]: SAE = 1, SSI = 9, PJI = 1, Intervention 2 [Antibiotic cement]: SAE = 2, SSI = 16, PJI = 2.

Wymenga 1992

Methods A prospective, randomized controlled non-blinded trial, at 27 hospitals in the Netherlands, mean follow-up duration of 13-months

Participants

Inclusion criteria: Patients undergoing a total hip replacement, hemiarthroplasty or a total knee arthroplasty.
Exclusion criteria: Allergy to study drug, or use of antibiotics < 48 hours before surgery, or administration of non-study antibiotics perioperatively, or malignancy, or previous or current infection in the joint, or use of gentamicin impregnated cement.

2,796 hip replacements were entered in the study, 145 were excluded because of protocol violations.

Mean age 69
Male to female ratio 1:4

Diagnosis: osteoarthritis 72%, rheumatoid arthritis 6%, fracture (recent) 10%, failed prosthesis 2%, osteotomy 4%, fracture osteosynthesis 2%, other earlier operations 1%, other reasons 3%.

Comorbidities Steroid use 2%, Diabetes 4%, Cardiac disease 14%, pulmonary disease 7%, preoperative infection 5%, Physical condition moderate 15%, Physical condition poor 1%

Interventions

Cefuroxime at a dose of 1,500mg was given intravenously to both groups upon inducing anaesthesia 30 minutes before surgery.

Intervention 1[Single-dose]: Single-dose cefuroxime 1,500 mg iv (n=1600)
Intervention 2 [Multiple-dose ≤ 24 hours]: Three doses of cefuroxime iv, one preoperative dose of 1,500mg followed by a second and third injection of 750mg given after 8 and 16 hours (n=1599)

No antibiotic in cement.

Outcomes surgical site infections, prosthetic joint infections, serious infections, and mortality within 365 days after surgery

Outcome definitions

The clinical end-point of the study was joint sepsis, reoperation or death.

PJI: Positive culture, evidence of sepsis, erythema.

Confirmed joint sepsis was defined as a positive bacteriologic culture at reoperation or a draining sinus. Strong evidence of sepsis was defined as four or more possible signs of infection. These two groups of conditions were analyzed together (Category I). In patients who only showed two or three possible signs of sepsis (Category II), a definite diagnosis could not be made. Patients with one or no signs of infections (Category III) were not suspected of having joint sepsis. The conditions that were defined as being possible infections at the follow-up examination were pain during weight bearing and/or at rest, tenderness of the wound, fever, an abnormal radiograph, erythrocyte sedimentation rate more than 35mm, positive culture from joint fluid aspirate, positive arthrogram, bone scan showing typical signs of infection, or increased C-reactive protein.
Wound infection in the postoperative period was defined as erythema more than 1 cm from the incision.

Superficial surgical site infection: Minor postoperative would-healing problems were defined as erythema more than 1 cm from the incision, pus suture, small wound dehiscence, necrosis of the would edge, and blisters.

Distant infections included pulmonary, urinary, skin and septicaemia.

Serious infections: of the reported distant infections; only septicemia was considered for this outcome for this Network meta-analysis.

Results Intervention 1 [Single-dose]: SAE = 63, SSI = 46, PJI = 11, serious infections = 5, mortality = 47 Intervention 2 [Multiple-dose ≤ 24 hours]: SAE = 48, SSI = 47, PJI = 6, serious infections = 4, mortality = 38.

Suter 1994

Methods A prospective, randomized controlled single-blinded clinical trial, single-center in Italy. A follow-up period of at least two years for each patient was planned. Patients were monitored for at least 12 months, but most were controlled for more than a year.

Participants

Inclusion criteria: all hospitalized patients ≥ 18 years of age undergoing elective surgery for total hip replacement

Exclusion criteria: history of allergic reactions to cephalosporins or glycopeptides, pregnancy, or lactation, or renal insufficiency, or local or systemic infections, or treatment with antibiotics within the previous two weeks.

260 patients were included in each treatment arm.

All patients were analysed for safety, but ten patients in the teicoplanin group and 14 in the cefamandole group did not have a sufficient follow-up period, and thus they were not included in the efficacy analysis.

Mean age 67

Female patients 72%

Diagnosis: osteoarthritis 87%, osteonecrosis <1%, femoral neck fracture 10%, rheumatoid arthritis 3%

Comorbidities Diabetes, venous insufficiency, renal failure (dialysis), hepatic cirrhosis and neoplasm 18%

Interventions

Patients were randomized to receive either teicoplanin 400mg as a single intravenous bolus 60-90 minutes before surgery, or cefamandole, administered with the same schedule, at an intravenous dose of 2g. A further intravenous dose (1g) of cefamandole was injected at the end of surgery. Administration of other antimicrobial agents was not allowed.

Intervention 1[Single-dose]: single dose of teicoplanin iv 400mg (n=260)

Intervention 2[Multiple-dose ≤ 24 hours]: 2-doses of Cefamandole iv 2g + 1g x1 (n=260)

Outcomes surgical site infections, prosthetic joint infections, venous thromboembolisms, and mortality within 365 days after surgery.

Outcome definitions The primary parameter of efficacy was the occurrence of deep infection or infection of the prosthetic device, characterized by pain, local tenderness, abnormal erythrocyte sedimentation rate, radiographic signs of infection or positive bacterial cultures of the periprosthetic space.

Secondary parameters of efficacy were wound complications, defined as erythema, serous exudate with negative culture, superficial haematoma with negative cultures, purulent or culture-positive serous exudate and superficial haematoma with positive cultures. The last two lesions were considered infective complications of the wound.

Venous thromboembolism: two cases of massive pulmonary embolism

Infections of other body sites were also recorded: respiratory tract infections (clinical signs of infection or production of mucous or purulent sputum and radiological signs of infection) and urinary tract infections (clinical signs and symptoms confirmed by at least one positive (> 105 cfu/ml) culture of clean-catch midstream urine). Finally, febrile morbidity, expressed as axillary body temperature of > 37.5 °C for two or more days, excluding the day of surgery, was monitored.

Results Intervention 1 [Single-dose]: SAE =0, SSI = 0, PJI = 0, venous thromboembolism = 0, mortality = 0, Intervention 2 [Multiple-dose ≤ 24 hours]: SAE = 3, SSI = 4, PJI = 0, venous thromboembolism = 2, mortality = 2 (one patient with VTE caused death, only counted once as SAE).

Mauerhan et al 1994

Methods RCT, prospective double-blinded multi-center study at 15 centers in the United States.

Participants

Inclusion criteria: adults who were to have an elective primary or revision total hip or knee arthroplasty

Exclusion criteria: Allergy to cephalosporins, or renal impairment, or neutropenia, or systemic or topical antibiotic use ≤ 7 days before operation, or evidence of infection at the time of the operation, or malignant tumor in the joint

Median age 65 years

Female patients 61%

Comorbidities Data for primary diagnosis and comorbidities represents data for the entire population of the study incl. primary and revision hips and knees. Diabetes mellitus 8%, Obesity (>30 % ideal weight) 26%, recent weight loss (> 20 % of body weight) 1%, concurrent corticosteroid therapy 13%, preoperative hospitalization (> 5 days) 1%, serum albumin level (< 35 g/L) 9%, previous operation on involved joint 25%

Interventions

1.5 grams of cefuroxime followed by 750 milligrams eight and sixteen hours later and then normal saline solution every eight hours for six additional doses (for one day of active-drug treatment and two days of placebo treatment) or one gram of cefazolin followed by one gram every eight hours for eight additional doses (for three days of active-drug treatment).

Intervention 1[Multiple-dose ≤ 24 hours]: 1 day of Cefuroxime iv: 1.5g + 750mg iv x2 and then normal saline solution every eight hours for six additional doses (n=285)

Intervention 2 [Multiple-dose > 1 day]: 3 days of Cefazolin:1g + 1g every 8 hours (n=265)

The first dose was administered fifteen to sixty minutes before the initial operative incision, for the patients who were to have a primary joint arthroplasty.

Outcomes
Surgical site infections and prosthetic joint infections within 90 and 365 days after surgery.

Outcome definitions
Clinical assessment was repeated at two to three months and at one year after the operation. Information on infections other than wound infections was collected through adverse-event reporting.

Wound infections were classified as superficial or deep, depending on whether they had developed above or below the fascia.

PJI: positive culture of purulent drainage from inflamed wound.

Results Intervention 1 [Multiple-dose ≤ 24 hours]: SAE =1, SSI = 4, PJI = 1, Intervention 2 [[Multiple-dose > 1 day]: SAE = 2, SSI = 4, PJI = 2 and within 90 days Intervention 1 [Multiple-dose ≤ 24 hours]: SAE =1, SSI = 4, PJI = 1, Intervention 2 [[Multiple-dose > 1 day]: SAE = 0, SSI = 2, PJI = 0.

S4 Table. Funding and conflict of interest statements of included studies

Reference<br>Author Year	Funding sources / Sources of support (as stated in the manuscript)	Conflict of interest (as stated in the manuscript)
Ericson 1973	Not stated.	Not stated.
Pollard 1979	We are grateful to Dr Clive Dash, Glaxo Limited, for supplying us with the antibiotics and for his help and advice with this trial.	Not stated.
Hill 1981	The Trial was supported by the Intitut National de la Sante et de la Recherche Medicale (INSERM).	Clinicians taking part were members of Groupe d'Etude du Traitement Préventif de l'infections dans les Arthroplasties.
Josefson 1981	Supported by the Swedish Medical Research Council (Project No. K79- I 6P-4636-058-5049044636). Palacos cum gentamicin supported by Essex Läkemedel AB, Sweden, subsidiary of the Schering Coprtporation, U.S.A.	Not stated.
Gunst 1984	Not stated.	Not stated.
Centulio 1988	Not stated.	Not stated.
McQueen 1990	The authors thank Glaxo Group Research Ltd. For supplying the antibiotic.	
Wymenga 1992	The authors wish to thank Glaxo B.V., The Netherlands, who supplied Cefuroxime and financially supported the study.	Not stated.
Suter 1994	Not stated.	Not stated.
Mauerhan 1994	Funds were received in total or partial support of the research or clinical study presented in this article. The funding source was Glaxo, Incorporated.	Although none of the authors have received or will receive benefits for personal or professional use from a commercial party related directly or indirectly to the subject of this article, benefits have been or will be received hut are directed solely to a research fund. Foundation, educational institution, or other non-profit organization with which one or more of the authors are associated.

S5 Fig Funnel Plots outcomes 365 days

Funnel plot of comparison: 1st Treatment duration vs 2nd Treatment duration, outcome: serious adverse events within 365 days after surgery

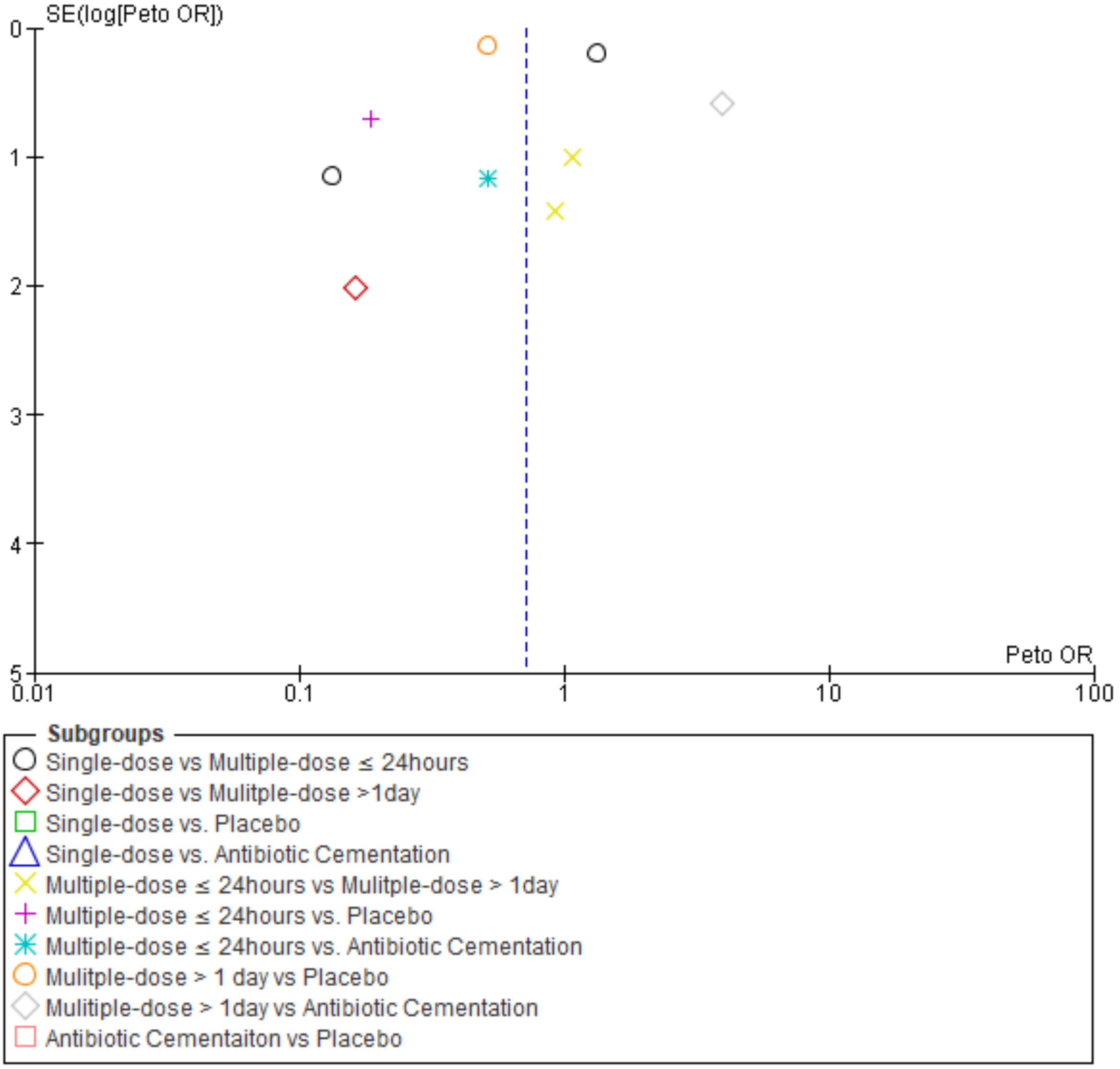

Funnel plot of comparison: 1st Treatment duration vs 2nd Treatment duration, outcome: surgical site infections within 365 days after surgery

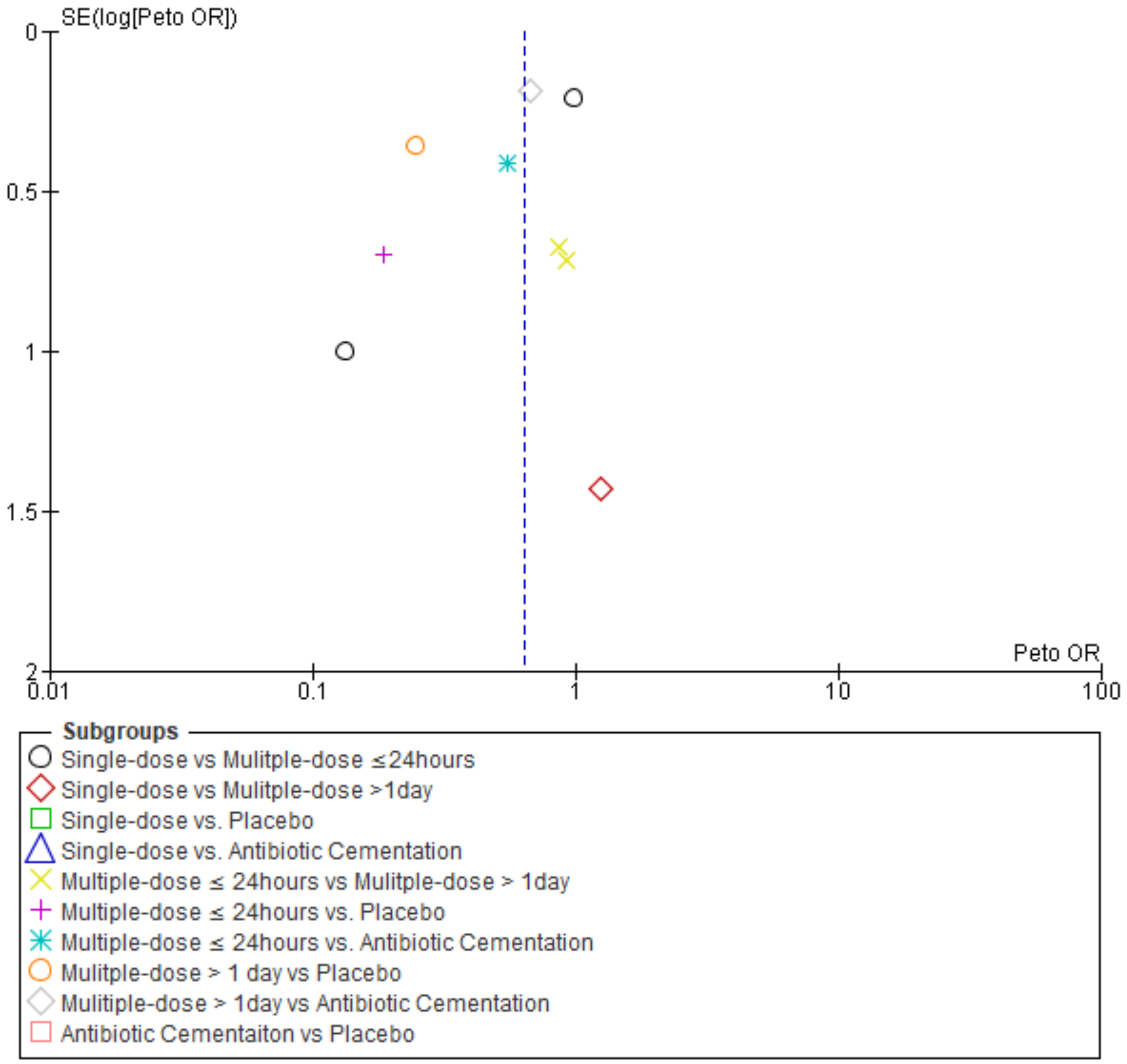

Funnel plot of comparison: 1st Treatment duration vs 2nd Treatment duration, outcome: prosthetic joint infections within 365 days after surgery

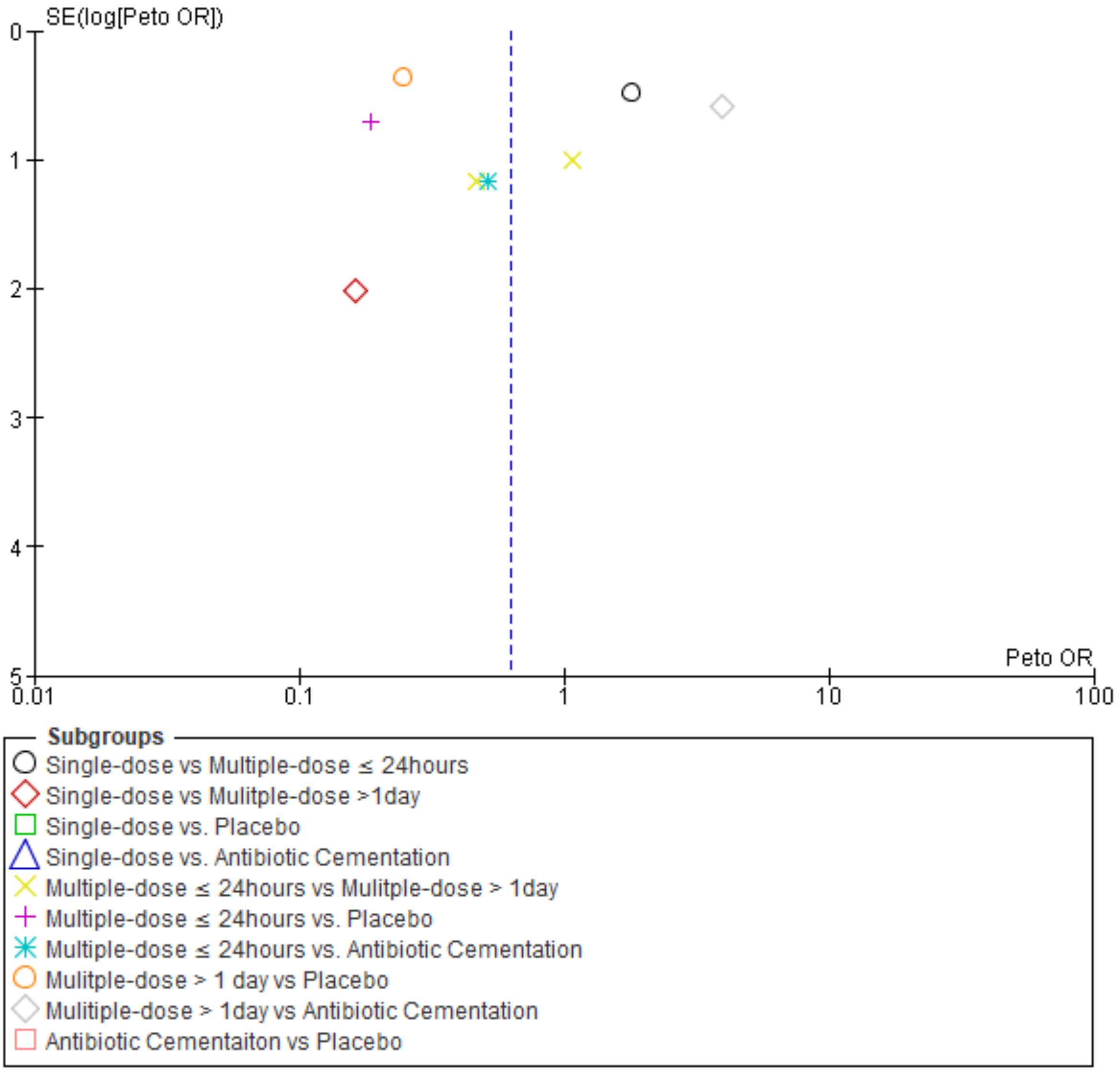

Funnel plot of comparison: 1st Treatment duration vs 2nd Treatment duration, outcome: serious infections within 365 days after surgery

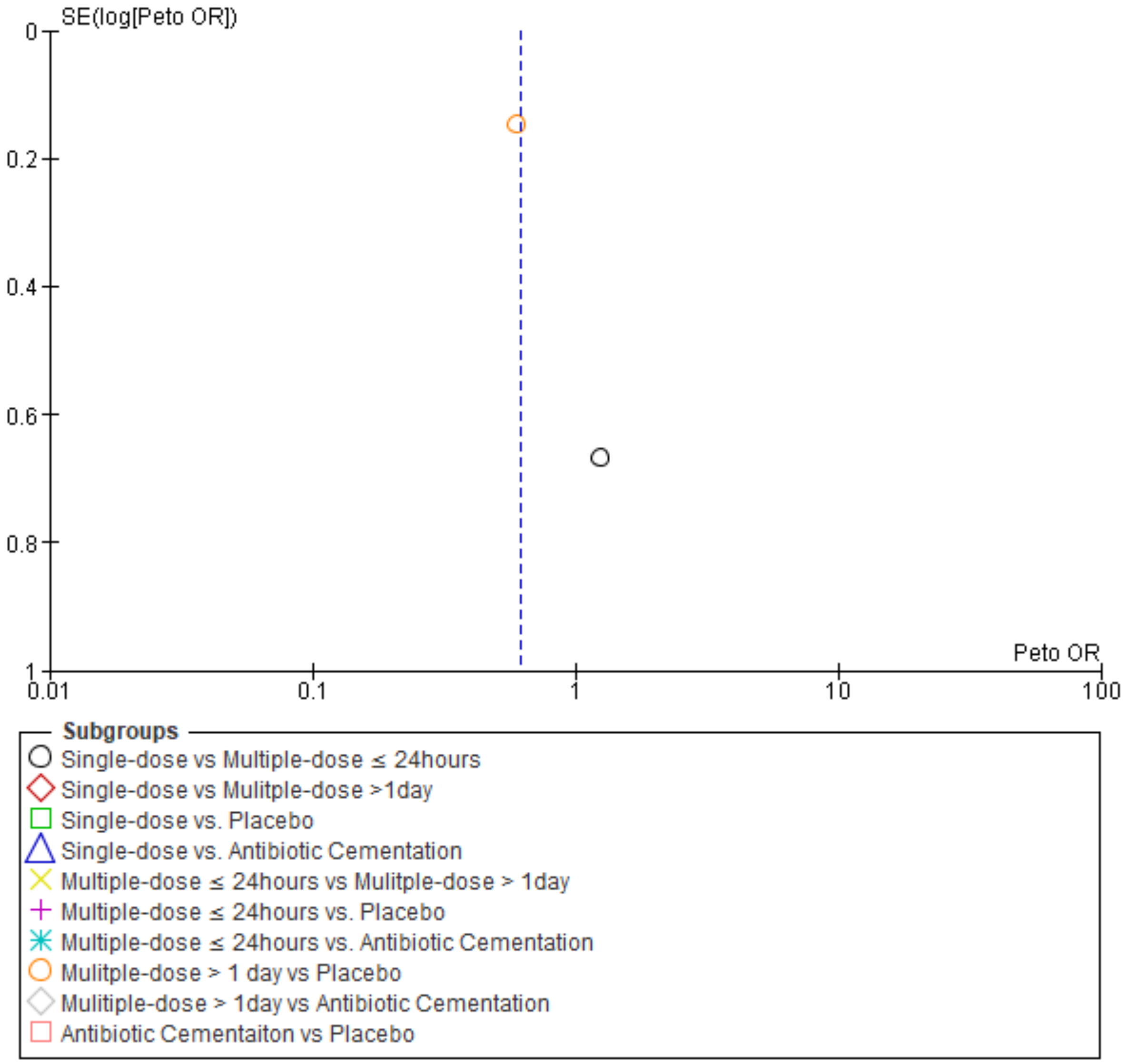

Funnel plot of comparison: 1st Treatment duration vs 2nd Treatment duration, outcome: major cardiovascular events within 365 days after surgery

Due to insufficient data, it is not possible to generate a funnel plot for the comparison between the first treatment duration and the second treatment duration regarding the outcome of major cardiovascular events within 365 days post-surgery.

Funnel plot of comparison: 1st Treatment duration vs 2nd Treatment duration, outcome: venous thromboembolisms within 365 days after surgery

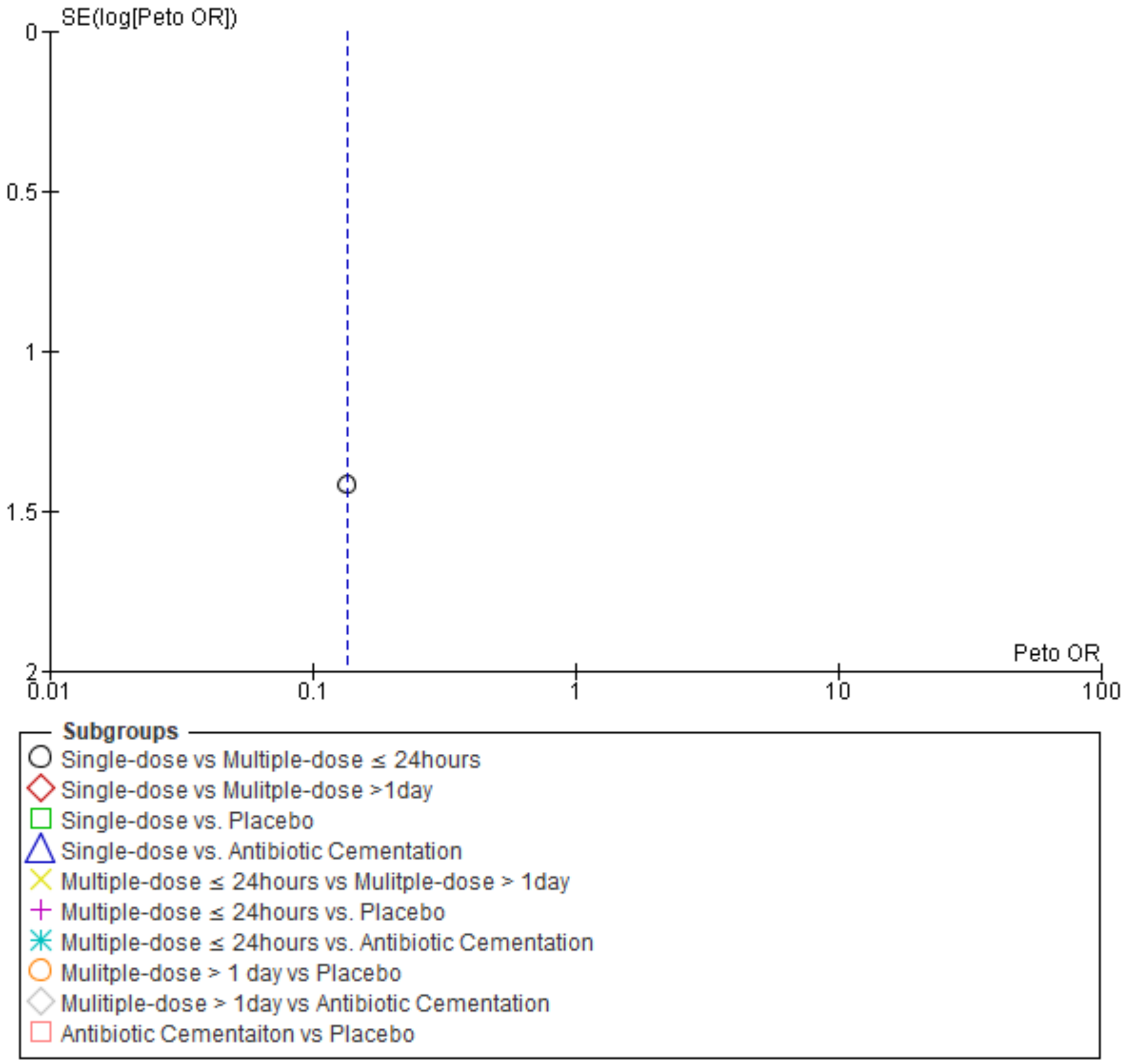

Funnel plot of comparison: 1st Treatment duration vs 2nd Treatment duration, outcome: mortality within 365 days after surgery

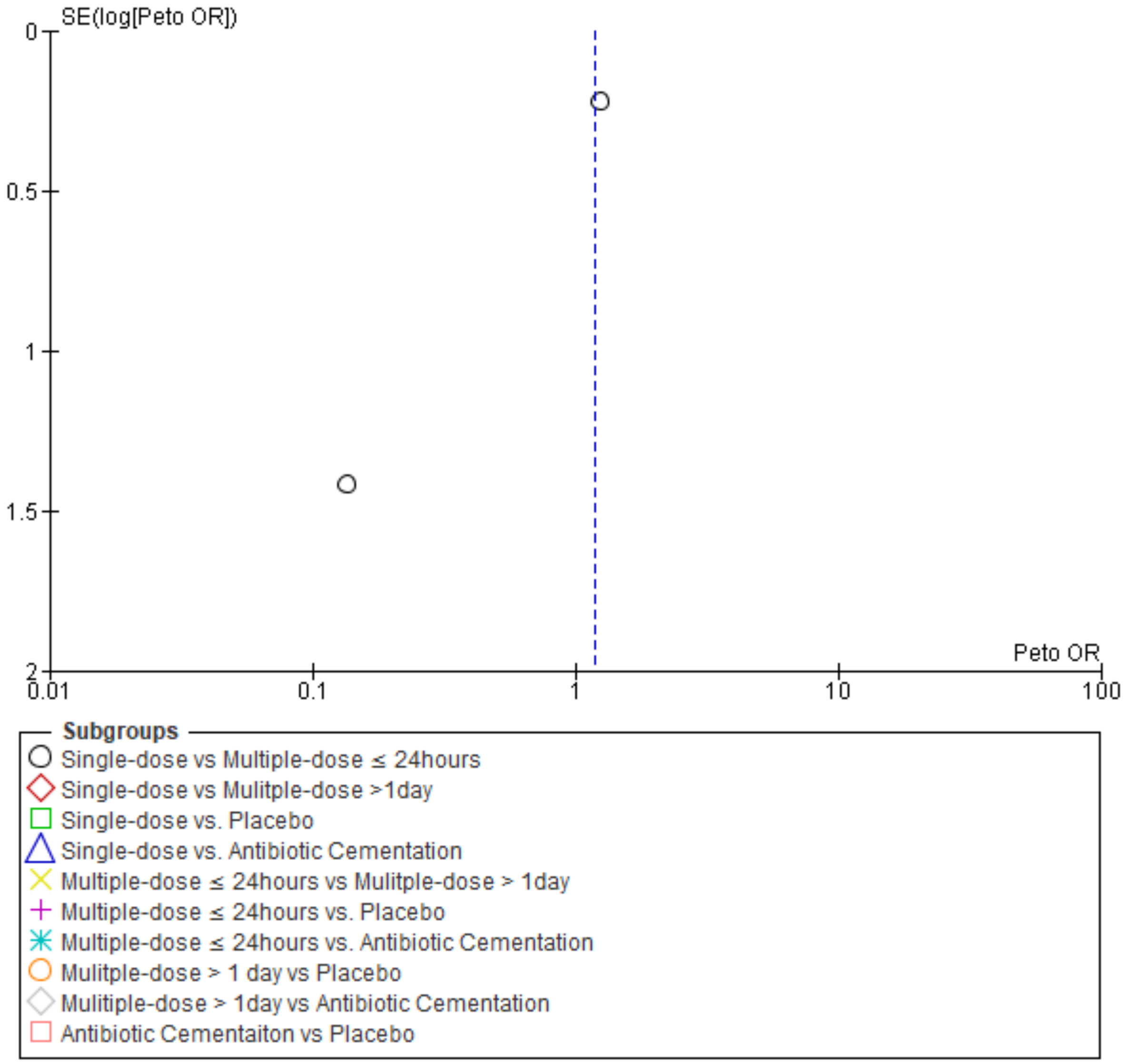

S2 Fig Funnel Plots outcomes within 90 days after surgery

Funnel plot of comparison: 1st Treatment duration vs. 2nd Treatment duration, outcome: serious adverse events

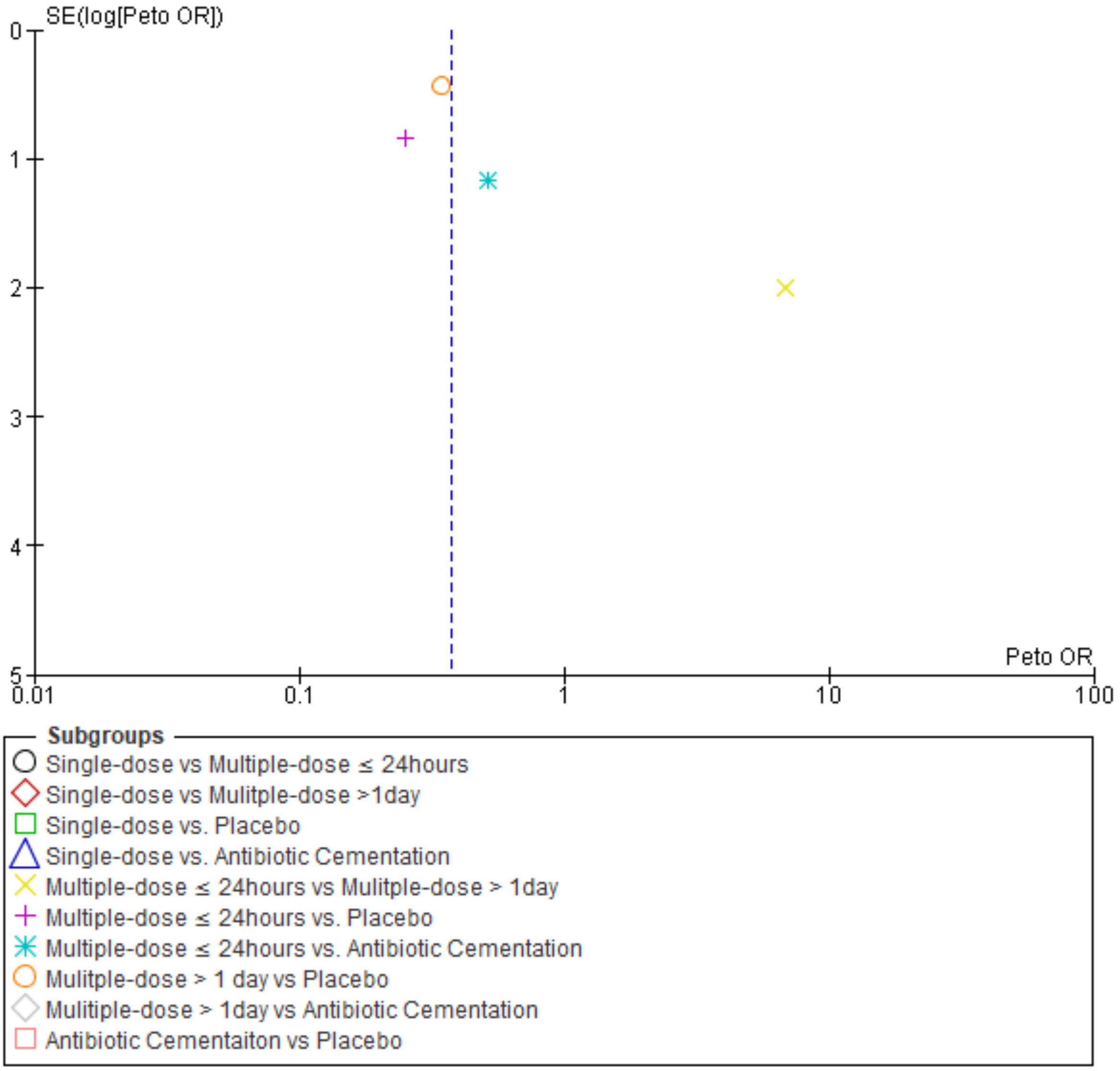

Funnel plot of comparison: 1st Treatment duration vs. 2nd Treatment duration, outcome: surgical site Infections

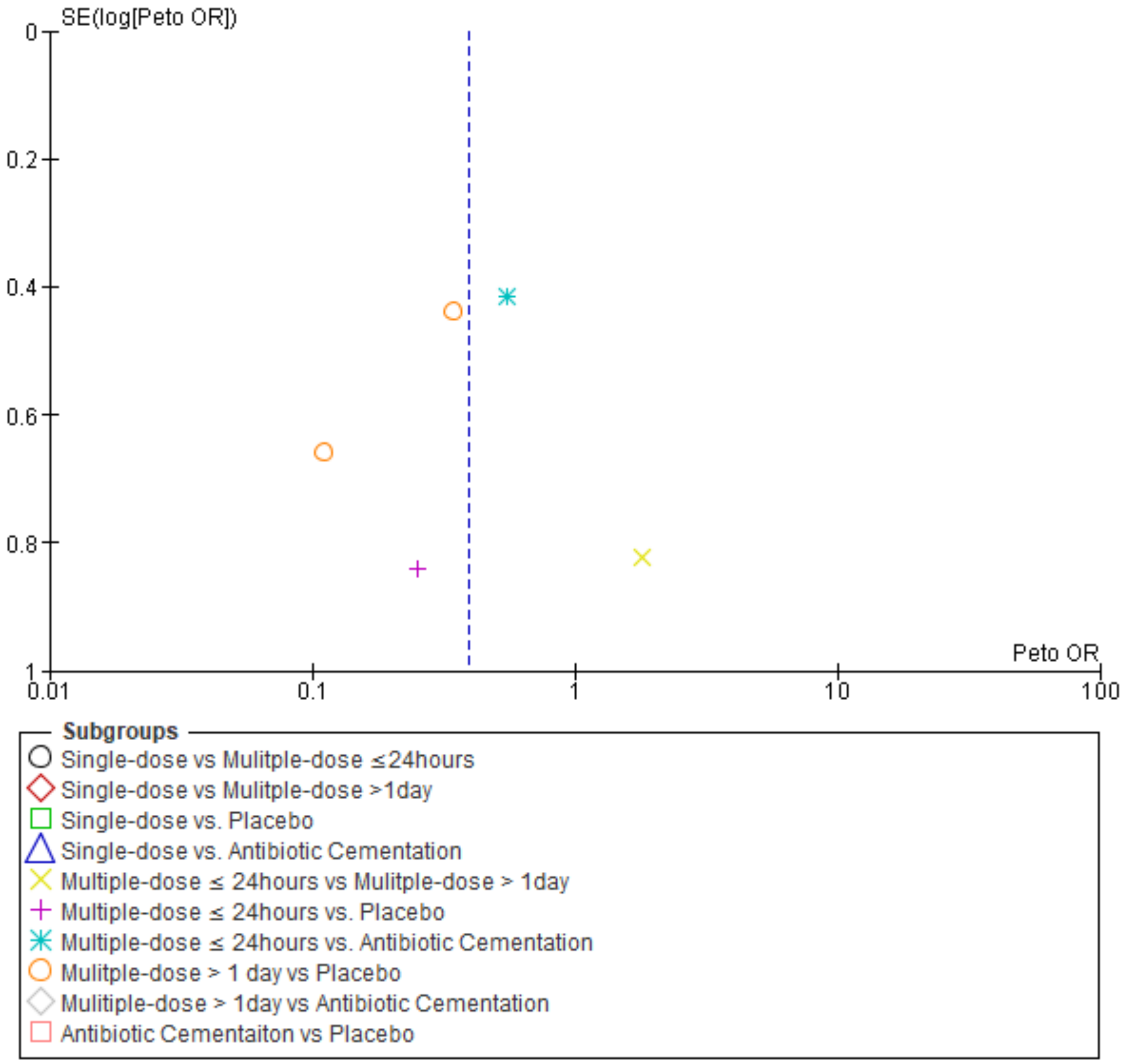

Funnel plot of comparison: 1st Treatment duration vs. 2nd Treatment duration, outcome: prosthetic joint infection

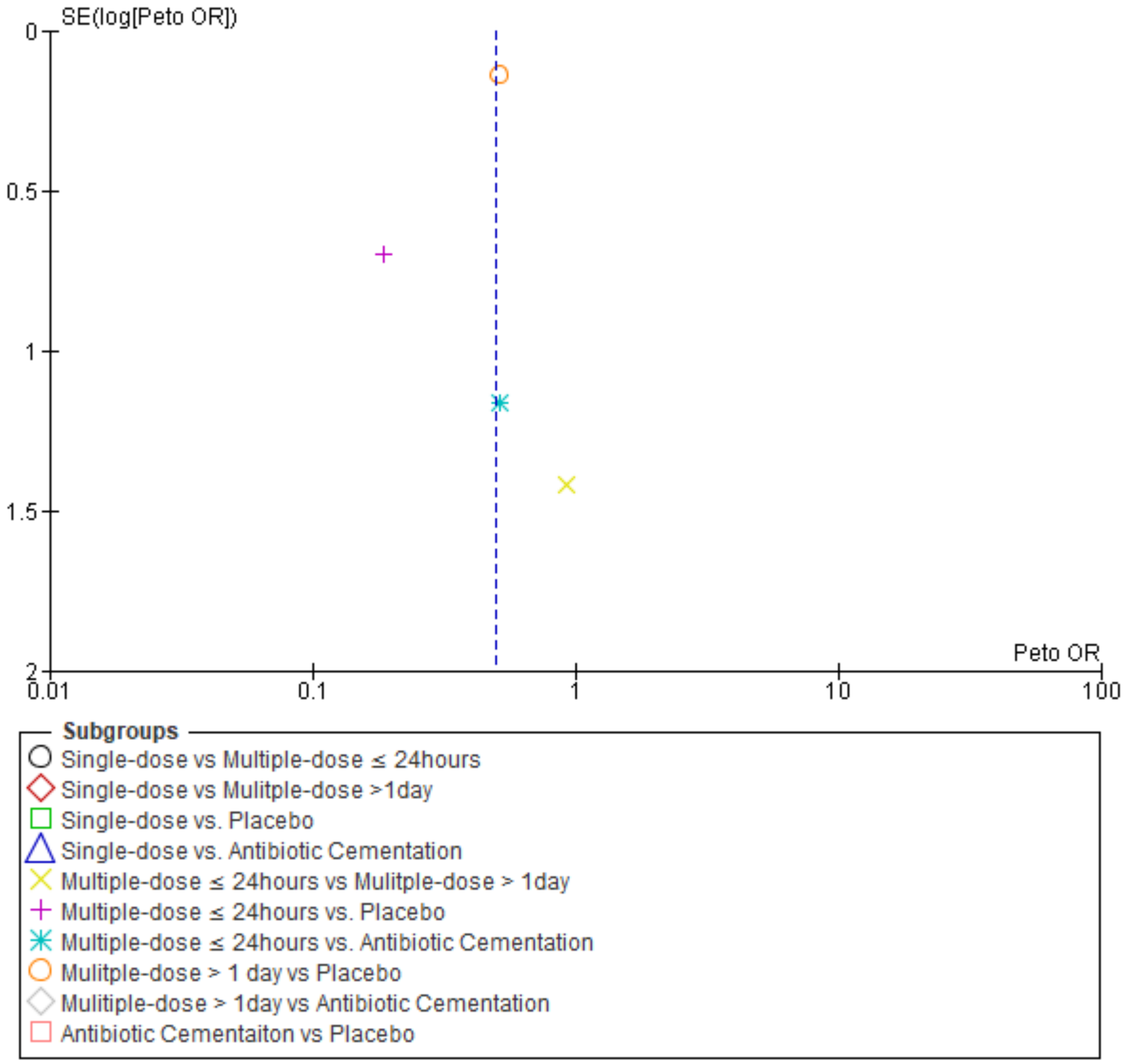

Funnel plot of comparison: 1st Treatment duration vs. 2nd treatment duration, outcome: serious infections

Due to insufficient data, it is not possible to generate a funnel plot for the comparison between the first treatment duration and the second treatment duration regarding the outcome of serious infections within 90 days post-surgery.

Funnel plot of comparison: 1st Treatment duration vs. 2nd Treatment duration, outcome: major cardiovascular events

Due to insufficient data, it is not possible to generate a funnel plot for the comparison between the first treatment duration and the second treatment duration regarding the outcome of major cardiovascular events within 90 days post-surgery.

Funnel plot of comparison: 1st Treatment duration vs. 2nd Treatment duration, outcome: venous thromboembolisms

Due to insufficient data, it is not possible to generate a funnel plot for the comparison between the first treatment duration and the second treatment duration regarding the outcome of venous thromboembolisms within 90 days post-surgery.

Funnel plot of comparison: 1st Treatment duration vs. 2nd Treatment duration, outcome: mortality

Due to insufficient data, it is not possible to generate a funnel plot for the comparison between the first treatment duration and the second treatment duration regarding the outcome of mortality within 90 days post-surgery.

S5 Table. Estimates of effects and quality ratings for comparison of treatment durations for outcomes 365 days and 90 days after primary THA

Estimates of effects and quality ratings for comparison of treatment durations to prevent serious adverse events 365 days after surgery

Table 2.1 Estimates of effects and quality ratings for comparison of antibiotic prophylaxis for prevention of SAEs 365 days after primary THA

	Direct Evidence	Network meta-analysis		
**Comparison**	**Odds Ratio (95% CI)**	**Odds Ratio (95% CI)**	**Certainty of Evidence**	**Prediction intervals**
Single-dose vs Multiple-dose ≤24hours	1.24 (0.86 to 1.81)	0.87 (0.20 to 3.73)	Very low*†	0.747 - 2.172
Single-dose vs Multiple-dose >1day	0.16 (0.00 to 8.41)	0.40 (0.07 to 2.42)	Very low *†	0.097 - 2.349
Single-dose vs. Placebo	–	0.11 (0.01 to 0.84)	Low*	0.047 - 1.204
Single-dose vs Antibiotic cement	–	1.50 (0.14 to 16.0)	Very low*†	0.189 - 14.751
Multiple dose ≤24hours vs Multiple-dose >1day	1.02 (0.20 to 5.09)	0.46 (0.11 to 1.90)	Very low*†	0.082 - 1.717
Multiple-dose ≤24hours vs Placebo	0.19 (0.05 to 0.73)	0.12 (0.02 to 0.69)	Low*	0.040 - 0.881
Multiple-dose ≤24hours vs Antibiotic Cement	0.51 (0.05 to 4.95)	1.73 (0.22 to 13.7)	Very low*†	0.157 - 10.952
Multiple-dose >1day vs Placebo	0.51 (0.39 to 0.67)	0.27 (0.06 to 1.16)	Low*	0.337 - 0.743
Multiple-dose >1day vs Antibiotic cement	3.91 (1.26 to 12.2)	3.77 (0.54 to 26.1)	Low*†‡	0.538 - 22.776
Antibiotic cement vs Placebo	–	0.07 (0.007 to 0.69)	Low*‡	0.037 - 0.550

*Within study bias
†Imprecision
‡Heterogeneity
CI = confidence interval

Comparison	Within-study bias*	Reporting bias	Indirectness	Imprecision†	Heterogeneity ‡	Incoherence	Confidence rating
Single-dose vs Multiple-dose ≤24hours	Major concerns	Some concerns	Some concerns	Major concerns	No concerns	No concerns	Very low*†
Single-dose vs Multiple-dose >1day	Major concerns	Some concerns	Some concerns	Major concerns	No concerns	No concerns	Very low*†
Single-dose vs. Placebo	Major concerns	Some concerns	Some concerns	No concerns	No concerns	No concerns	Low*
Single-dose vs Antibiotic cement	Major concerns	Some concerns	Some concerns	Major concerns	No concerns	No concerns	Very low*†
Multiple-dose ≤24hours vs Multiple-dose >1day	Major concerns	Some concerns	Some concerns	Major concerns	No concerns	No concerns	Very low*†
Multiple-dose ≤24hours vs Placebo	Major concerns	Some concerns	Some concerns	No concerns	No concerns	No concerns	Low*
Multiple-dose ≤24hours vs Antibiotic cement	Major concerns	Some concerns	Some concerns	Major concerns	No concerns	No concerns	Very low*†
Multiple-dose >1day vs Placebo	Major concerns	Some concerns	Some concerns	Some concerns	No concerns	No concerns	Low*
Multiple-dose >1day vs Antibiotic cement	Some concerns	Some concerns	Some concerns	Major concerns	Some concerns	No concerns	Low*†‡
Antibiotic cement vs Placebo	Major concerns	Some concerns	Some concerns	No concerns	Some concerns	No concerns	Low*‡

Estimates of effects and quality ratings for comparison of treatment durations to prevent surgical site infections within 365 days after surgery

Table 2.2 Estimates of effects and quality ratings for comparison of antibiotic prophylaxis for prevention of SSIs 365 days after primary THA

	Direct Evidence	Network meta-analysis	
**Comparison**	**Odds Ratio (95% CI)**	**Odds Ratio (95% CI)**	**Certainty of Evidence**
Single-dose vs Multiple-dose ≤24hours	0.90 (0.60 to 1.35)	0.90 (0.53 to 1.51)	Very low *†
Single-dose vs Multiple-dose >1day	1.24 (0.08 to 20.3)	0.68 (0.28 to 1.70)	Very low *†
Single-dose vs. Placebo	—	0.15 (0.04 to 0.50)	Low*
Single-dose vs Antibiotic cement	—	0.52 (0.21 to 1.30)	Very low*†
Multiple-dose ≤24hours vs Multiple-dose >1day	0.88 (0.34 to 2.31)	0.76 (0.35 to 1.66)	Very low *†
Multiple-dose ≤24hours vs Placebo	0.19 (0.05 to 0.73)	0.17 (0.05 to 0.51)	Low*
Multiple-dose ≤24hours vs Antibiotic cement	0.55 (0.24 to 1.24)	0.58 (0.27 to 1.27)	Very low*†
Multiple-dose >1day vs Placebo	0.25 (0.12 to 0.50)	0.22 (0.08 to 0.57)	Low*
Multiple-dose >1day vs Antibiotic cement	0.67 (0.46 to 0.97)	0.76 (0.49 to 1.20)	Low *†‡
Antibiotic cement vs Placebo	—	0.28 (0.10 to 0.81)	Low *‡

*Within study bias
†Imprecision
‡Heterogeneity
CI = confidence interval

Comparison	Within-study bias*	Reporting bias	Indirectness	Imprecision†	Heterogeneity ‡	Incoherence	Confidence rating
Single-dose vs Multiple-dose ≤24hours	Major concerns	Some concerns	Some concerns	Major concerns	No concerns	No concerns	Very low*†
Single-dose vs Multiple-dose >1day	Major concerns	Some concerns	Some concerns	Major concerns	No concerns	No concerns	Very low*†
Single-dose vs. Placebo	Major concerns	Some concerns	Some concerns	No concerns	No concerns	No concerns	Low*
Single-dose vs Antibiotic cement	Major concerns	Some concerns	Some concerns	Major concerns	No concerns	No concerns	Very low*†
Multiple-dose ≤24hours vs Multiple-dose >1day	Major concerns	Some concerns	Some concerns	Major concerns	No concerns	No concerns	Very low*†
Multiple-dose ≤24hours vs Placebo	Major concerns	Some concerns	Some concerns	No concerns	No concerns	No concerns	Low*
Multiple-dose ≤24hours vs Antibiotic cement	Major concerns	Some concerns	Some concerns	Major concerns	No concerns	No concerns	Very low*†
Multiple-dose >1day vs Placebo	Major concerns	Some concerns	Some concerns	No concerns	No concerns	No concerns	Low*
Multiple-dose >1day vs Antibiotic cement	Some concerns	Some concerns	Some concerns	Some concerns	Some concerns	No concerns	Low*†‡
Antibiotic cement vs Placebo	Major concerns	Some concerns	Some concerns	No concerns	Some concerns	No concerns	Low *‡

Estimates of effects and quality ratings for comparison of treatment durations to prevent prosthetic joint infections within 365 days after surgery

Table 2.3 Estimates of effects and quality ratings for comparison of antibiotic prophylaxis for prevention of SAEs 365 days after primary THA

	Direct Evidence	Network meta-analysis	
**Comparison**	**Odds Ratio (95% CI)**	**Odds Ratio (95% CI)**	**Certainty of Evidence**
Single-dose vs Multiple-dose ≤24hours	1.81 (0.7 to 4.68)	1.42 (0.45 to 4.51)	Very low*†
Single-dose vs Multiple-dose >1day	0.16 (0.00 to 8.41)	0.52 (0.11 to 2.46)	Very low*†
Single-dose vs. Placebo	–	0.09 (0.02 to 0.49)	Very low*‡
Single-dose vs Antibiotic cement	–	1.63 (0.22 to 11.9)	Very low*†
Multiple-dose ≤24hours vs Multiple-dose >1day	0.75 (0.17 to 3.33)	0.36 (0.093 to 1.42)	Very low*†
Multiple-dose ≤24hours vs Placebo	0.19 (0.05 to 0.73)	0.07 (0.02 to 0.29)	Low*
Multiple-dose ≤24hours vs Antibiotic cement	0.51 (0.05 to 4.95)	1.14 (0.18 to 7.15)	Low†
Multiple-dose >1day vs Placebo	0.25 (0.12 to 0.50)	0.18 (0.06 to 0.51)	Low*
Multiple-dose >1day vs Antibiotic cement	3.91 (1.26 to 12.2)	3.15 (0.70 to 14.2)	Low*†‡
Antibiotic cement vs Placebo	–	0.06 (0.01 to 0.33)	Low*

*Within study bias
†Imprecision
‡Heterogeneity
CI = confidence interval

Comparison	Within-study bias*	Reporting bias	Indirectness	Imprecision†	Heterogeneity‡	Incoherence	Confidence rating
Single-dose vs Multiple-dose ≤24hours	Major concerns	Some concerns	Some concerns	Major concerns	No concerns	No concerns	Very low*†
Single-dose vs Multiple-dose >1day	Major concerns	Some concerns	Some concerns	Major concerns	No concerns	No concerns	Very low*†
Single-dose vs. Placebo	Major concerns	Some concerns	Some concerns	No concerns	Major concerns	No concerns	Very low*‡
Single-dose vs Antibiotic cement	Major concerns	Some concerns	Some concerns	Major concerns	No concerns	No concerns	Very low*†
Multiple dose ≤24hours vs Multiple-dose >1day	Major concerns	Some concerns	Some concerns	Major concerns	No concerns	No concerns	Very low*†
Multiple dose ≤24hours vs Placebo	Major concerns	Some concerns	Some concerns	No concerns	No concerns	No concerns	Low*
Multiple dose ≤24hours vs Antibiotic cement	Some concerns	Some concerns	Some concerns	Major concerns	No concerns	No concerns	Low†
Multiple-dose >1day vs Placebo	Major concerns	Some concerns	Some concerns	No concerns	No concerns	No concerns	Low*
Multiple-dose >1day vs Antibiotic cement	Some concerns	Some concerns	Some concerns	Some concerns	Some concerns	No concerns	Low*†‡
Antibiotic Cement vs Placebo	Major concerns	Some concerns	Some concerns	No concerns	No concerns	No concerns	Low*

Estimates of effects and quality ratings for comparison of treatment durations to prevent serious infections 365 days after surgery

Table 2.4 Estimates of effects and quality ratings for comparison of antibiotic prophylaxis for prevention of Serious Infections 365 days after primary THA

	Direct Evidence	Network meta-analysis	
Comparison	Odds Ratio (95% CI)	Odds Ratio (95% CI)	Certainty of Evidence
Single-dose vs Multiple-dose ≤24hours	1.25 (0.34 to 4.82)	1.25 (not estimable)	—
Single-dose vs Multiple-dose >1day	—	0.04 (not estimable)	—
Single-dose vs. Placebo	—	0.02 (not estimable)	—
Single-dose vs Antibiotic cement	—	—	—
Multiple dose ≤24hours vs Multiple-dose >1day	—	0.03 (not estimable)	—
Multiple-dose ≤24hours vs placebo	—	0.02 (not estimable)	—
Multiple-dose ≤24hours vs Antibiotic cement	—	—	—
Multiple-dose >1day vs placebo	0.60 (0.45 to 0.79)	0.59 (not estimable)	—
Multiple-dose >1day vs Antibiotic cement	—	—	—
Antibiotic cement vs Placebo	—	—	—

Estimates of effects and quality ratings for comparison of treatment durations to prevent major cardiovascular events within 365 days after surgery

Due to insufficient data, it is not possible to provide estimates of effects and quality ratings for the comparison of treatment durations regarding the outcome of major cardiovascular events within 365 days after surgery.

Estimates of effects and quality ratings for comparison of treatment durations to prevent venous thromboembolisms within 365 days after surgery

Due to insufficient data, it is not possible to provide estimates of effects and quality ratings for the comparison of treatment durations regarding the outcome of venous thromboembolisms within 365 days after surgery.

Estimates of effects and quality ratings for comparison of treatment durations to prevent mortality within 365 days after surgery

Table 2.7 Estimates of effects and quality ratings for comparison of antibiotic prophylaxis and Mortality 365 days after primary THA

	Direct Evidence	Network meta-analysis	
**Comparison**	**Odds Ratio (95% CI)**	**Odds Ratio (95% CI)**	**Certainty of Evidence**
Single-dose vs Multiple-dose ≤24hours	1.18 (0.77 to 1.80)	0.72 (not estimable)	—
Single-dose vs Multiple-dose >1day	—	—	—
Single-dose vs. Placebo	—	—	—
Single-dose vs Antibiotic cement	—	—	—
Multiple-dose ≤24hours vs Multiple-dose >1day	—	—	—
Multiple-dose ≤24hours vs placebo	—	—	—
Multiple-dose ≤24hours vs Antibiotic cement	—	—	—
Multiple-dose >1day vs placebo	—	—	—
Multiple-dose >1day vs Antibiotic cement	—	—	—
Antibiotic cement vs Placebo	—	—	—

Estimates of effects and quality ratings for comparison of treatment durations to prevent serious adverse events 90 days after surgery

Table 2.8 Estimates of effects and quality ratings for comparison of antibiotic prophylaxis for prevention of SAE 90 days after primary THA

	Direct Evidence	Network meta-analysis	
**Comparison**	**Odds Ratio (95% CI)**	**Odds Ratio (95% CI)**	**Certainty of Evidence**
Single-dose vs Multiple-dose ≤24hours	–		
Single-dose vs Multiple-dose >1day	–		
Single-dose vs. Placebo	–		
Single-dose vs Antibiotic cement	–		
Multiple-dose ≤24hours vs Multiple-dose >1day	6.79 (0.13 to 344)	1.46 (Not estimable)	Not estimable
Multiple-dose ≤24hours vs placebo	0.25 (0.05 to 1.29)	0.24 (Not estimable)	Not estimable
Multiple-dose ≤24hours vs Antibiotic cement	0.51 (0.05 to 4.95)	0.58 (Not estimable)	Not estimable
Multiple-dose >1day vs placebo	0.35 (0.15 to 0.82)	0.16 (Not estimable)	Not estimable
Multiple-dose >1day vs Antibiotic cement	–	0.39 (Not estimable)	Not estimable
Antibiotic cement vs Placebo	–	0.41 (Not estimable)	Not estimable

Comparison	Within-study bias*	Reporting bias	Indirectness	Imprecision†	Heterogeneity‡	Incoherence	Confidence rating
Single-dose vs Multiple-dose ≤24hours							
Single-dose vs Multiple-dose >1day							
Single-dose vs. Placebo							
Single-dose vs Antibiotic cement							
Multiple-dose ≤24hours vs Multiple-dose >1day	Major concerns	Some concerns	Some concerns				Not estimable
Multiple-dose ≤24hours vs Placebo	Major concerns	Some concerns	Some concerns				Not estimable
Multiple-dose ≤24hours vs Antibiotic cement	Some concerns	Some concerns	Some concerns				Not estimable
Multiple-dose >1day vs Placebo	Major concerns	Some concerns	Some concerns				Not estimable
Multiple-dose >1day vs Antibiotic cement	Major concerns	Some concerns	Some concerns				Not estimable
Antibiotic cement vs Placebo	Major concerns	Some concerns	Some concerns				Not estimable

Estimates of effects and quality ratings for comparison of treatment durations to prevent surgical site infections within 90 days after surgery

Table 2.9 Estimates of effects and quality ratings for comparison of antibiotic prophylaxis for prevention of SSIs within 90 days after primary THA

	Direct Evidence	Network meta-analysis	
**Comparison**	**Odds Ratio (95% CI)**	**Odds Ratio (95% CI)**	**Certainty of Evidence**
Single-dose vs Multiple-dose ≤24hours	—	—	—
Single-dose vs Multiple-dose >1day	—	—	—
Single-dose vs. Placebo	—	—	—
Single-dose vs Antibiotic cement	—	—	—
Multiple-dose ≤24hours vs Multiple-dose >1day	1.80 (0.36 – 8.98)	1.30 (0.20 – 8.53)	Low†
Multiple-dose ≤24hours vs Placebo	0.25 (0.05 – 1.29)	0.24 (0.03 – 2.08)	Very low*†
Multiple-dose ≤24hours vs Antibiotic cement	0.55 (0.24 – 1.24)	0.60 (0.10 – 3.74)	Very low*‡
Multiple-dose >1day vs placebo	0.24 (0.12 – 0.50)	0.19 (0.03-1.07)	Very low*‡
Multiple-dose >1day vs Antibiotic cement	—	0.46 (0.09 – 2.37)	Low†
Antibiotic cement vs Placebo	—	0.41 (0.05 – 3.42)	Very low*‡

*Within study bias
†Imprecision
‡Heterogeneity
CI = confidence interval

**Comparison**	**Within-study bias***	**Reporting bias**	**Indirectness**	**Imprecision†**	**Heterogeneity‡**	**Incoherence**	**Confidence rating**
Multiple-dose ≤24hours vs Multiple-dose >1day	Some concerns	Some concerns	Some concerns	Major concerns	No concerns	No concerns	Low†
Multiple-dose ≤24hours vs Placebo	Major concerns	Some concerns	Some concerns	Major concerns	No concerns	No concerns	Very low*†
Multiple-dose ≤24hours vs Antibiotic cement	Major concerns	Some concerns	Some concerns	No concerns	Major concerns	No concerns	Very low*‡
Multiple-dose >1day vs placebo	Major concerns	Some concerns	Some concerns	No concerns	Major concerns	No concerns	Very low*‡
Multiple-dose >1day vs Antibiotic cement	Some concerns	Some concerns	Some concerns	Major concerns	No concerns	No concerns	Low†
Antibiotic cement vs Placebo	Major concerns	Some concerns	Some concerns	Major concerns	No concerns	No concerns	Very low*‡

Estimates of effects and quality ratings for comparison of treatment durations to prevent prosthetic joint infections within 90 days after surgery

Table 2.10 Estimates of effects and quality ratings for comparison of antibiotic prophylaxis for prevention of PJI within 90 days after primary THA

	Direct Evidence	Network meta-analysis	
**Comparison**	**Odds Ratio (95% CI)**	**Odds Ratio (95% CI)**	**Certainty of Evidence**
Single-dose vs Multiple-dose ≤24hours	—	—	—
Single-dose vs Multiple-dose >1day	—	—	—
Single-dose vs. Placebo	—	—	—
Single-dose vs Antibiotic cement	—	—	—
Multiple-dose ≤24hours vs Multiple-dose >1day	0.92 (0.06 – 14.7)	1.46 (Not estimable)	Not estimable
Multiple-dose ≤24hours vs placebo	0.19 (0.05 – 0.73)	0.24 (Not estimable)	Not estimable
Multiple-dose ≤24hours vs Antibiotic cement	0.51 (0.05 – 4.95)	0.58 (Not estimable)	Not estimable
Multiple-dose >1day vs placebo	0.51 (0.39 – 0.67)	0.16 (Not estimable)	Not estimable
Multiple-dose >1day vs Antibiotic cement	—	0.39 (Not estimable)	Not estimable
Antibiotic cement vs Placebo	—	0.41 (Not estimable)	Not estimable

Comparison	Within-study bias*	Reporting bias	Indirectness	Imprecision†	Heterogeneity‡	Incoherence	Confidence rating
Multiple-dose ≤24hours vs Multiple-dose >1day	Major concerns	Some concerns	Some concerns				Not estimable
Multiple-dose ≤24hours vs Placebo	Major concerns	Some concerns	Some concerns				Not estimable
Multiple-dose ≤24hours vs Antibiotic cement	Some concerns	Some concerns	Some concerns				Not estimable
Multiple-dose >1day vs Placebo	Major concerns	Some concerns	Some concerns				Not estimable
Multiple-dose >1day vs Antibiotic cement	Some concerns	Some concerns	Some concerns				Not estimable
Antibiotic cement vs Placebo	Major concerns	Some concerns	Some concerns				Not estimable

Estimates of effects and quality ratings for comparison of treatment durations to prevent serious infections 90 days after surgery

Due to insufficient data, it is not possible to provide estimates of effects and quality ratings for the comparison of treatment durations for the outcome of serious infections within 90 after surgery.

Estimates of effects and quality ratings for comparison of treatment durations to prevent major cardiovascular events within 90 days after surgery

Due to insufficient data, it is not possible to provide estimates of effects and quality ratings for the comparison of treatment durations for the outcome of major cardiovascular events within 90 days after surgery.

Estimates of effects and quality ratings for comparison of treatment durations to prevent venous thromboembolisms within 90 days after surgery

Due to insufficient data, it is not possible to provide estimates of effects and quality ratings for the comparison of treatment durations for the outcome of venous thromboembolisms within 90 after surgery.

Estimates of effects and quality ratings for comparison of treatment durations to prevent mortality within 90 days after surgery

Due to insufficient data, it is not possible to provide estimates of effects and quality ratings for the comparison of treatment durations for the outcome of mortality within 90 after surgery.

S6 Table. Cochrane Risk of Bias tool 2

+	Low risk
!	Some concerns
-	High risk

D1	Randomisation process
D2	Deviations from the intended interventions
D3	Missing outcome data
D4	Measurement of the outcome
D5	Selection of the reported result

**Study ID**	**Outcome**	**D1**	**D2**	**D3**	**D4**	**D5**	**Overall**
Ericson 1973	SSI	!	!	+	+	!	!
Gunst 1984	PJI	!	-	-	!	!	-
Hill 1981	PJI	+	!	+	-	+	-
Hill 1981	PJI	+	!	+	-	-	-
Josefsson 1981	PJI	!	!	+	+	!	!
Josefsson 1981	SSI	!	!	+	!	!	!
Mauerhan 1994	PJI	+	!	-	+	!	-
Mauerhan 1994	SSI	+	+	-	!	!	-
Centulio 1988	PJI	!	-	!	-	-	-
Centulio 1988	SSI	!	!	-	-	-	-
McQueen 1990	PJI	+	!	+	+	!	!
McQueen 1990	PJI	!	!	+	-	!	-
Wymenga 1992	PJI	-	-	-	-	!	-
Wymenga 1992	SSI	-	-	-	-	!	-
Wymenga 1992	Serious infections	-	-	-	!	!	-
Wymenga 1992	Mortality	-	-	-	+	!	-
Suter 1994	PJI	!	-	+	-	!	-
Suter 1994	Serious infections	!	-	+	-	!	-

Suter 1994	SSI	!	-	+	-	!	-
Suter 1994	Mortality	!	-	+	!	!	-
Pollard 1997	PJI	!	-	+	-	!	-
Pollard 1997	SSI	!	-	+	!	!	-

S3 Fig Forest Plots outcomes 365 days

Forest plot of odds ratio on serious adverse events comparing treatment durations

Study or Subgroup	1st Treatment Events	1st Treatment Total	2nd Treatment Events	2nd Treatment Total	Weight	Peto Odds Ratio Peto, Fixed, 95% CI	Year
**3.2.1 Single-dose vs Multiple-dose ≤ 24hours**							
Wymenga1992	63	1600	48	1599	97.3%	1.32 [0.91, 1.93]	1992
Suter1994	0	260	3	260	2.7%	0.13 [0.01, 1.30]	1994
**Subtotal (95% CI)**		**1860**		**1859**	**100.0%**	**1.24 [0.86, 1.81]**	
Total events	63		51				
Heterogeneity: Chi² = 3.80, df = 1 (P = 0.05); I² = 74%							
Test for overall effect: Z = 1.14 (P = 0.25)							
**3.2.2 Single-dose vs Mulitple-dose >1day**							
Centulio1988	0	56	1	69	100.0%	0.16 [0.00, 8.41]	1988
**Subtotal (95% CI)**		**56**		**69**	**100.0%**	**0.16 [0.00, 8.41]**	
Total events	0		1				
Heterogeneity: Not applicable							
Test for overall effect: Z = 0.90 (P = 0.37)							
**3.2.3 Single-dose vs. Placebo**							
**Subtotal (95% CI)**		**0**		**0**		**Not estimable**	
Total events	0		0				
Heterogeneity: Not applicable							
Test for overall effect: Not applicable							
**3.2.4 Single-dose vs. Antibiotic Cementation**							
**Subtotal (95% CI)**		**0**		**0**		**Not estimable**	
Total events	0		0				
Heterogeneity: Not applicable							
Test for overall effect: Not applicable							
**3.2.5 Multiple-dose ≤ 24hours vs Mulitple-dose > 1day**							
Pollard1979	2	146	2	157	66.5%	1.08 [0.15, 7.73]	1979
Mauerhan1994	1	285	1	261	33.5%	0.92 [0.06, 14.71]	1994
**Subtotal (95% CI)**		**431**		**418**	**100.0%**	**1.02 [0.20, 5.09]**	
Total events	3		3				
Heterogeneity: Chi² = 0.01, df = 1 (P = 0.93); I² = 0%							
Test for overall effect: Z = 0.02 (P = 0.98)							
**3.2.6 Multiple-dose ≤ 24hours vs. Placebo**							
Gunst1984	1	46	8	47	100.0%	0.19 [0.05, 0.73]	1984
**Subtotal (95% CI)**		**46**		**47**	**100.0%**	**0.19 [0.05, 0.73]**	
Total events	1		8				
Heterogeneity: Not applicable							
Test for overall effect: Z = 2.41 (P = 0.02)							
**3.2.7 Multiple-dose ≤ 24hours vs. Antibiotic Cementation**							
McQueen1990	1	190	2	190	100.0%	0.51 [0.05, 4.95]	1990
**Subtotal (95% CI)**		**190**		**190**	**100.0%**	**0.51 [0.05, 4.95]**	
Total events	1		2				
Heterogeneity: Not applicable							
Test for overall effect: Z = 0.58 (P = 0.56)							
**3.2.8 Mulitple-dose > 1 day vs Placebo**							
Hill1981	84	1070	154	1067	100.0%	0.51 [0.39, 0.67]	1981
**Subtotal (95% CI)**		**1070**		**1067**	**100.0%**	**0.51 [0.39, 0.67]**	
Total events	84		154				
Heterogeneity: Not applicable							
Test for overall effect: Z = 4.84 (P < 0.00001)							
**3.2.9 Mulitiple-dose > 1day vs Antibiotic Cementation**							
Josefsson1981	10	834	2	851	100.0%	3.91 [1.26, 12.16]	1981
**Subtotal (95% CI)**		**834**		**851**	**100.0%**	**3.91 [1.26, 12.16]**	
Total events	10		2				
Heterogeneity: Not applicable							
Test for overall effect: Z = 2.35 (P = 0.02)							
**3.2.10 Antibiotic Cementaiton vs Placebo**							
**Subtotal (95% CI)**		**0**		**0**		**Not estimable**	
Total events	0		0				
Heterogeneity: Not applicable							
Test for overall effect: Not applicable							

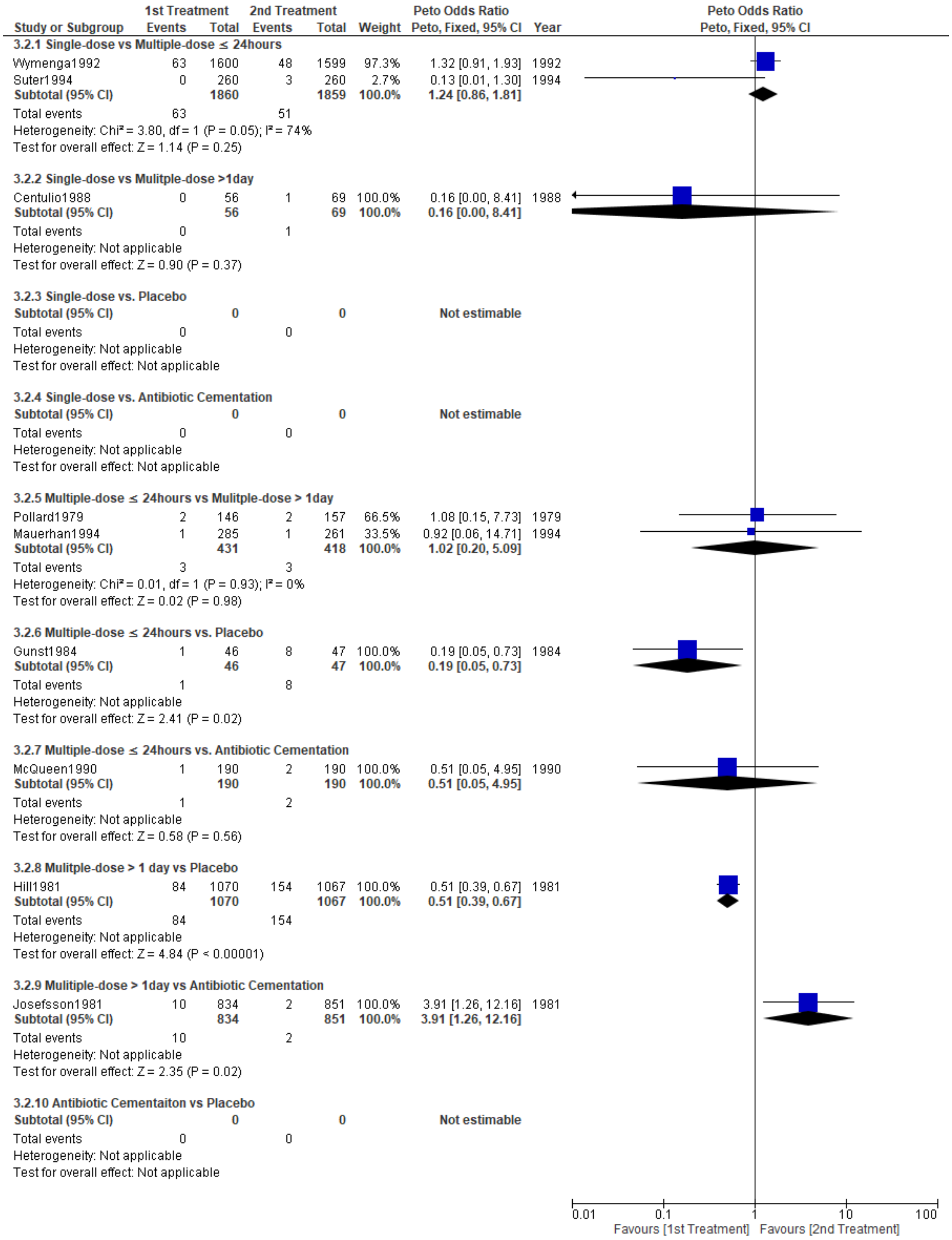

Forest plot of odds ratio on surgical site infections comparing treatment durations

Study or Subgroup	1st Treatment Events	1st Treatment Total	2nd Treatment Events	2nd Treatment Total	Weight	Peto Odds Ratio Peto, Fixed, 95% CI	Year
**3.1.1 Single-dose vs Mulitple-dose ≤24hours**							
Wymenga1992	46	1600	47	1599	95.8%	0.98 [0.65, 1.48]	1992
Suter1994	0	260	4	260	4.2%	0.13 [0.02, 0.96]	1994
**Subtotal (95% CI)**		**1860**		**1859**	**100.0%**	**0.90 [0.60, 1.35]**	
Total events	46		51				
Heterogeneity: Chi^2 = 3.77, df = 1 (P = 0.05); I^2 = 73%							
Test for overall effect: Z = 0.52 (P = 0.60)							
**3.1.2 Single-dose vs Mulitple-dose >1day**							
Centulio1988	1	56	1	69	100.0%	1.24 [0.08, 20.29]	1988
**Subtotal (95% CI)**		**56**		**69**	**100.0%**	**1.24 [0.08, 20.29]**	
Total events	1		1				
Heterogeneity: Not applicable							
Test for overall effect: Z = 0.15 (P = 0.88)							
**3.1.3 Single-dose vs. Placebo**							
**Subtotal (95% CI)**		**0**		**0**		**Not estimable**	
Total events	0		0				
Heterogeneity: Not applicable							
Test for overall effect: Not applicable							
**3.1.4 Single-dose vs. Antibiotic Cementation**							
**Subtotal (95% CI)**		**0**		**0**		**Not estimable**	
Total events	0		0				
Heterogeneity: Not applicable							
Test for overall effect: Not applicable							
**3.1.5 Multiple-dose ≤ 24hours vs Mulitple-dose > 1day**							
Pollard1979	4	146	5	157	52.6%	0.86 [0.23, 3.23]	1979
Mauerhan1994	4	285	4	261	47.4%	0.91 [0.23, 3.70]	1994
**Subtotal (95% CI)**		**431**		**418**	**100.0%**	**0.88 [0.34, 2.31]**	
Total events	8		9				
Heterogeneity: Chi^2 = 0.00, df = 1 (P = 0.95); I^2 = 0%							
Test for overall effect: Z = 0.25 (P = 0.80)							
**3.1.6 Multiple-dose ≤ 24hours vs. Placebo**							
Gunst1984	1	46	8	47	100.0%	0.19 [0.05, 0.73]	1984
**Subtotal (95% CI)**		**46**		**47**	**100.0%**	**0.19 [0.05, 0.73]**	
Total events	1		8				
Heterogeneity: Not applicable							
Test for overall effect: Z = 2.41 (P = 0.02)							
**3.1.7 Multiple-dose ≤ 24hours vs. Antibiotic Cementation**							
McQueen1990	9	190	16	190	100.0%	0.55 [0.24, 1.24]	1990
**Subtotal (95% CI)**		**190**		**190**	**100.0%**	**0.55 [0.24, 1.24]**	
Total events	9		16				
Heterogeneity: Not applicable							
Test for overall effect: Z = 1.45 (P = 0.15)							
**3.1.8 Mulitple-dose > 1 day vs Placebo**							
Hill1981	5	1070	27	1067	100.0%	0.25 [0.12, 0.50]	1981
**Subtotal (95% CI)**		**1070**		**1067**	**100.0%**	**0.25 [0.12, 0.50]**	
Total events	5		27				
Heterogeneity: Not applicable							
Test for overall effect: Z = 3.93 (P < 0.0001)							
**3.1.9 Mulitiple-dose > 1day vs Antibiotic Cementation**							
Josefsson1981	49	834	73	851	100.0%	0.67 [0.46, 0.97]	1981
**Subtotal (95% CI)**		**834**		**851**	**100.0%**	**0.67 [0.46, 0.97]**	
Total events	49		73				
Heterogeneity: Not applicable							
Test for overall effect: Z = 2.14 (P = 0.03)							
**3.1.10 Antibiotic Cementaiton vs Placebo**							
**Subtotal (95% CI)**		**0**		**0**		**Not estimable**	
Total events	0		0				
Heterogeneity: Not applicable							
Test for overall effect: Not applicable							

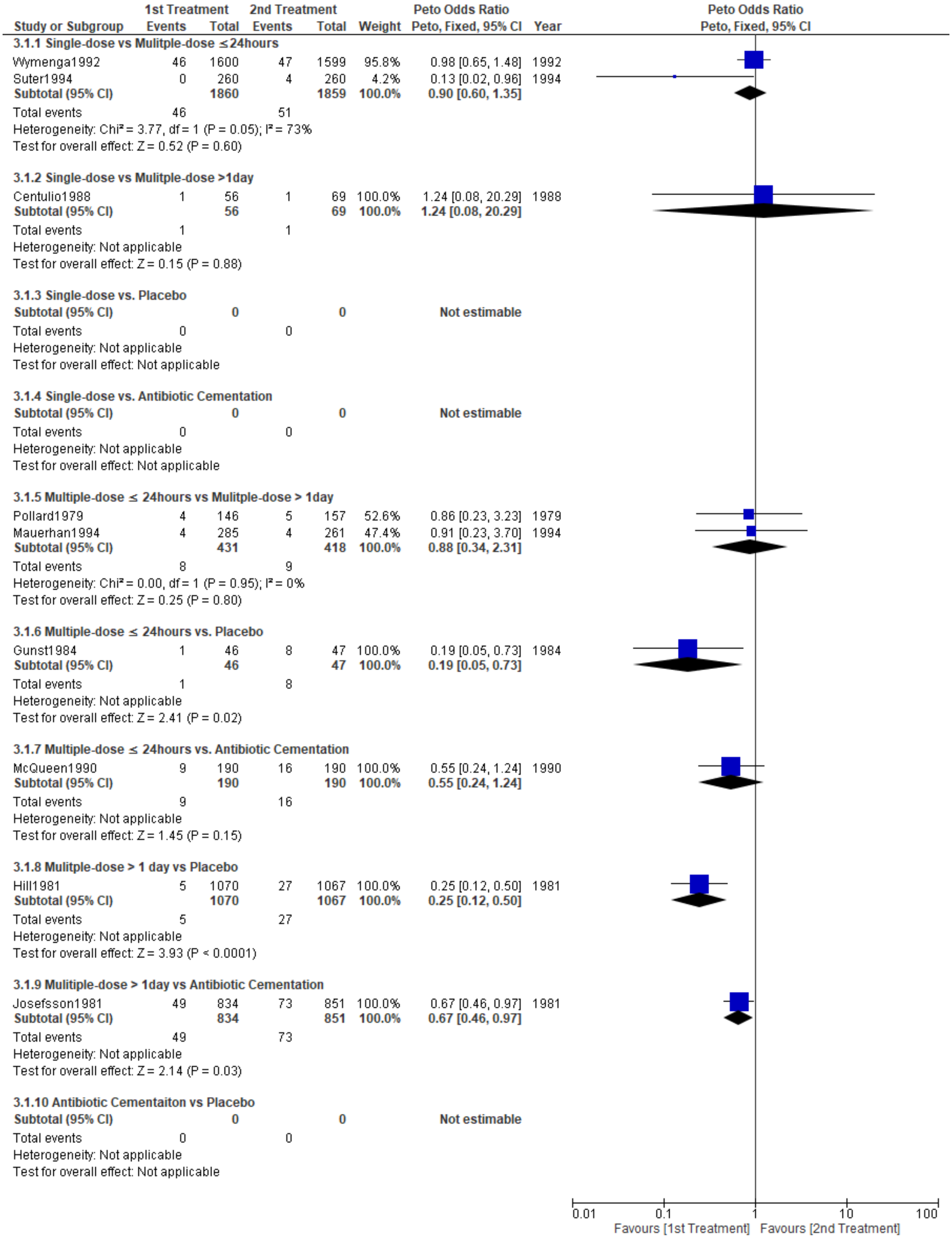

Forest plot of odds ratio on prosthetic joint infections comparing treatment durations

Study or Subgroup	1st Treatment Events	1st Treatment Total	2nd Treatment Events	2nd Treatment Total	Weight	Peto Odds Ratio Peto, Fixed, 95% CI	Year
**3.3.1 Single-dose vs Multiple-dose ≤ 24hours**							
Wymenga1992	11	1600	6	1599	100.0%	1.81 [0.70, 4.68]	1992
Suter1994	0	260	0	260		Not estimable	1994
**Subtotal (95% CI)**		**1860**		**1859**	**100.0%**	**1.81 [0.70, 4.68]**	
Total events	11		6				
Heterogeneity: Not applicable							
Test for overall effect: Z = 1.21 (P = 0.22)							
**3.3.2 Single-dose vs Mulitple-dose >1day**							
Centulio1988	0	56	1	69	100.0%	0.16 [0.00, 8.41]	1988
**Subtotal (95% CI)**		**56**		**69**	**100.0%**	**0.16 [0.00, 8.41]**	
Total events	0		1				
Heterogeneity: Not applicable							
Test for overall effect: Z = 0.90 (P = 0.37)							
**3.3.3 Single-dose vs. Placebo**							
**Subtotal (95% CI)**		**0**		**0**		**Not estimable**	
Total events	0		0				
Heterogeneity: Not applicable							
Test for overall effect: Not applicable							
**3.3.4 Single-dose vs. Antibiotic Cementation**							
**Subtotal (95% CI)**		**0**		**0**		**Not estimable**	
Total events	0		0				
Heterogeneity: Not applicable							
Test for overall effect: Not applicable							
**3.3.5 Multiple-dose ≤ 24hours vs Mulitple-dose > 1day**							
Pollard1979	2	146	2	157	57.0%	1.08 [0.15, 7.73]	1979
Mauerhan1994	1	285	2	261	43.0%	0.47 [0.05, 4.53]	1994
**Subtotal (95% CI)**		**431**		**418**	**100.0%**	**0.75 [0.17, 3.33]**	
Total events	3		4				
Heterogeneity: Chi² = 0.29, df = 1 (P = 0.59); I² = 0%							
Test for overall effect: Z = 0.37 (P = 0.71)							
**3.3.6 Multiple-dose ≤ 24hours vs. Placebo**							
Gunst1984	1	46	8	47	100.0%	0.19 [0.05, 0.73]	1984
**Subtotal (95% CI)**		**46**		**47**	**100.0%**	**0.19 [0.05, 0.73]**	
Total events	1		8				
Heterogeneity: Not applicable							
Test for overall effect: Z = 2.41 (P = 0.02)							
**3.3.7 Multiple-dose ≤ 24hours vs. Antibiotic Cementation**							
McQueen1990	1	190	2	190	100.0%	0.51 [0.05, 4.95]	1990
**Subtotal (95% CI)**		**190**		**190**	**100.0%**	**0.51 [0.05, 4.95]**	
Total events	1		2				
Heterogeneity: Not applicable							
Test for overall effect: Z = 0.58 (P = 0.56)							
**3.3.8 Mulitple-dose > 1 day vs Placebo**							
Hill1981	5	1070	27	1067	100.0%	0.25 [0.12, 0.50]	1981
**Subtotal (95% CI)**		**1070**		**1067**	**100.0%**	**0.25 [0.12, 0.50]**	
Total events	5		27				
Heterogeneity: Not applicable							
Test for overall effect: Z = 3.93 (P < 0.0001)							
**3.3.9 Mulitiple-dose > 1day vs Antibiotic Cementation**							
Josefsson1981	10	834	2	851	100.0%	3.91 [1.26, 12.16]	1981
**Subtotal (95% CI)**		**834**		**851**	**100.0%**	**3.91 [1.26, 12.16]**	
Total events	10		2				
Heterogeneity: Not applicable							
Test for overall effect: Z = 2.35 (P = 0.02)							
**3.3.10 Antibiotic Cementaiton vs Placebo**							
**Subtotal (95% CI)**		**0**		**0**		**Not estimable**	
Total events	0		0				
Heterogeneity: Not applicable							
Test for overall effect: Not applicable							

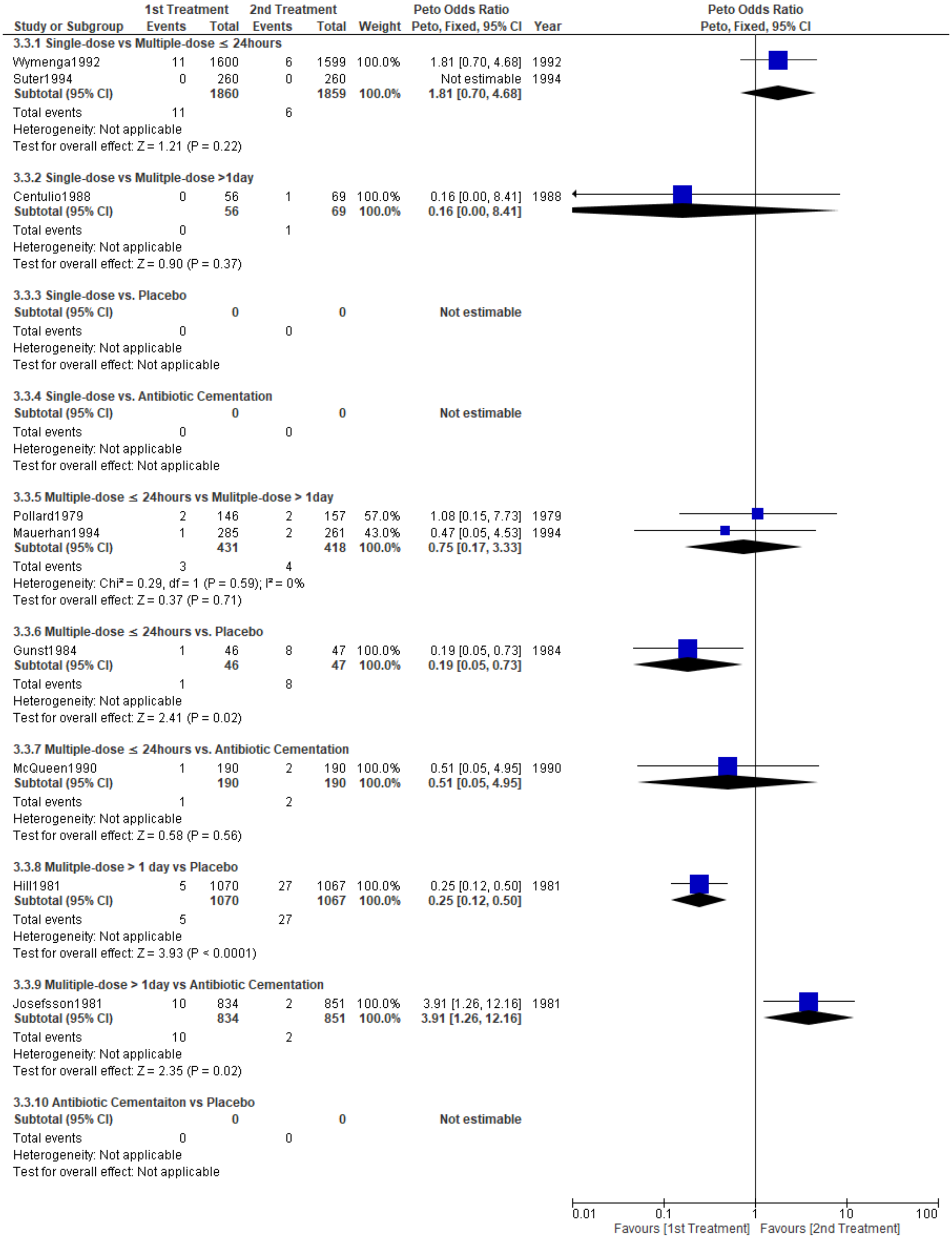

Forest plot of odds ratio on serious infections comparing treatment durations

Study or Subgroup	1st Treatment Events	1st Treatment Total	2nd Treatment Events	2nd Treatment Total	Weight	Peto Odds Ratio Peto, Fixed, 95% CI	Year
**3.4.1 Single-dose vs Multiple-dose ≤ 24hours**							
Wymenga1992	5	1600	4	1599	100.0%	1.25 [0.34, 4.62]	1992
**Subtotal (95% CI)**		**1600**		**1599**	**100.0%**	**1.25 [0.34, 4.62]**	
Total events	5		4				
Heterogeneity: Not applicable							
Test for overall effect: Z = 0.33 (P = 0.74)							
**3.4.2 Single-dose vs Mulitple-dose >1day**							
**Subtotal (95% CI)**		**0**		**0**		**Not estimable**	
Total events	0		0				
Heterogeneity: Not applicable							
Test for overall effect: Not applicable							
**3.4.3 Single-dose vs. Placebo**							
**Subtotal (95% CI)**		**0**		**0**		**Not estimable**	
Total events	0		0				
Heterogeneity: Not applicable							
Test for overall effect: Not applicable							
**3.4.4 Single-dose vs. Antibiotic Cementation**							
**Subtotal (95% CI)**		**0**		**0**		**Not estimable**	
Total events	0		0				
Heterogeneity: Not applicable							
Test for overall effect: Not applicable							
**3.4.5 Multiple-dose ≤ 24hours vs Mulitple-dose > 1day**							
**Subtotal (95% CI)**		**0**		**0**		**Not estimable**	
Total events	0		0				
Heterogeneity: Not applicable							
Test for overall effect: Not applicable							
**3.4.6 Multiple-dose ≤ 24hours vs. Placebo**							
**Subtotal (95% CI)**		**0**		**0**		**Not estimable**	
Total events	0		0				
Heterogeneity: Not applicable							
Test for overall effect: Not applicable							
**3.4.7 Multiple-dose ≤ 24hours vs. Antibiotic Cementation**							
**Subtotal (95% CI)**		**0**		**0**		**Not estimable**	
Total events	0		0				
Heterogeneity: Not applicable							
Test for overall effect: Not applicable							
**3.4.8 Mulitple-dose > 1 day vs Placebo**							
Hill1981	79	1070	127	1067	100.0%	0.60 [0.45, 0.79]	1981
**Subtotal (95% CI)**		**1070**		**1067**	**100.0%**	**0.60 [0.45, 0.79]**	
Total events	79		127				
Heterogeneity: Not applicable							
Test for overall effect: Z = 3.54 (P = 0.0004)							
**3.4.9 Mulitiple-dose > 1day vs Antibiotic Cementation**							
**Subtotal (95% CI)**		**0**		**0**		**Not estimable**	
Total events	0		0				
Heterogeneity: Not applicable							
Test for overall effect: Not applicable							
**3.4.10 Antibiotic Cementaiton vs Placebo**							
**Subtotal (95% CI)**		**0**		**0**		**Not estimable**	
Total events	0		0				
Heterogeneity: Not applicable							
Test for overall effect: Not applicable							

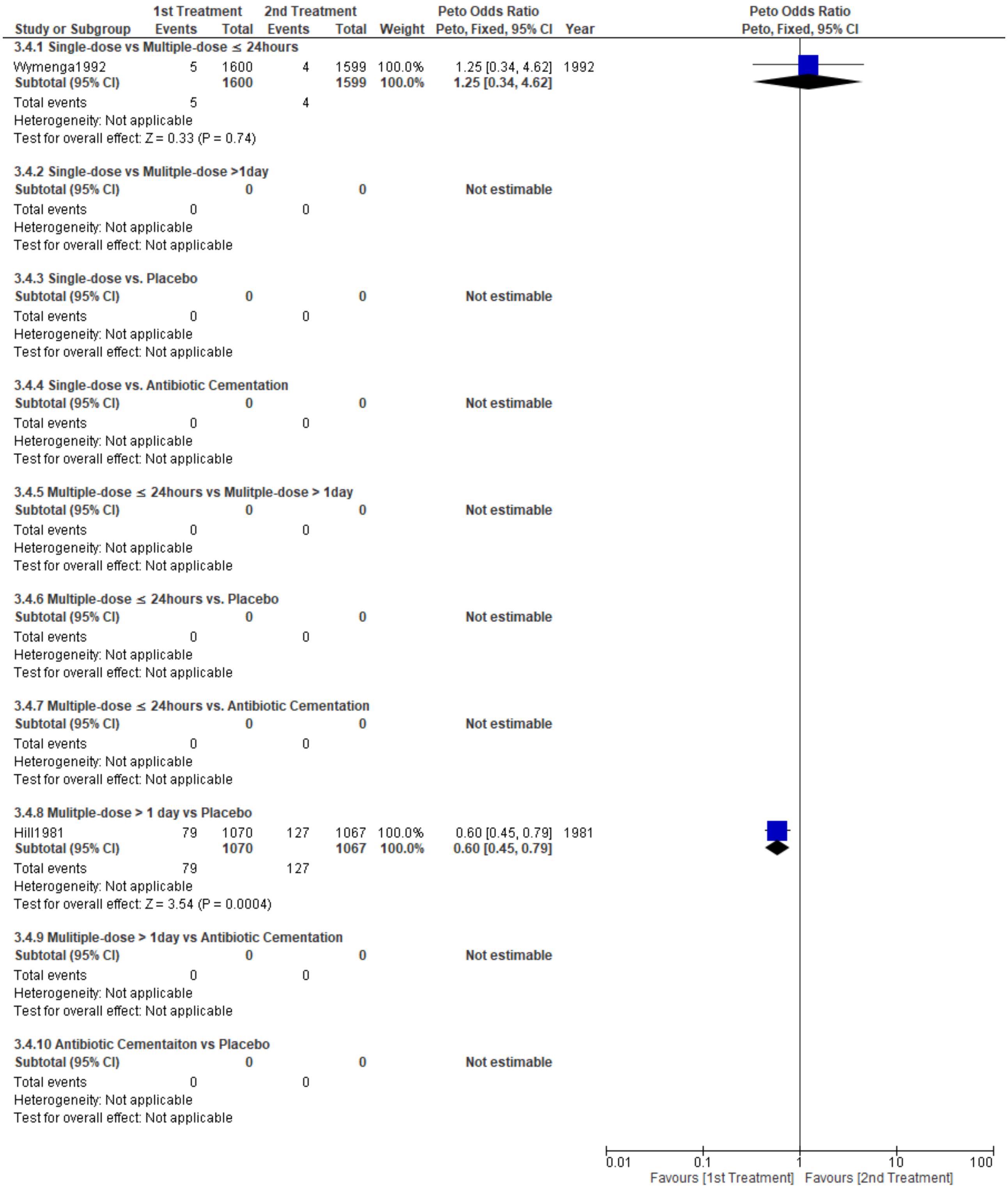

Forest plot of odds ratio on major cardiovascular events comparing treatment durations

Due to insufficient data, it is not possible to provide a forest plot for the comparison of treatment durations for the outcome of major cardiovascular events within 365 after surgery.

Forest plot of odds ratio on venous thromboembolisms comparing treatment durations

Study or Subgroup	1st Treatment Events	1st Treatment Total	2nd Treatment Events	2nd Treatment Total	Weight	Peto Odds Ratio Peto, Fixed, 95% CI	Year
**3.6.1 Single-dose vs Multiple-dose ≤ 24hours**							
Suter1994	0	260	2	260	100.0%	0.13 [0.01, 2.16]	1994
**Subtotal (95% CI)**		**260**		**260**	**100.0%**	**0.13 [0.01, 2.16]**	
Total events	0		2				
Heterogeneity: Not applicable							
Test for overall effect: Z = 1.42 (P = 0.16)							
**3.6.2 Single-dose vs Mulitple-dose >1day**							
**Subtotal (95% CI)**		**0**		**0**		**Not estimable**	
Total events	0		0				
Heterogeneity: Not applicable							
Test for overall effect: Not applicable							
**3.6.3 Single-dose vs. Placebo**							
**Subtotal (95% CI)**		**0**		**0**		**Not estimable**	
Total events	0		0				
Heterogeneity: Not applicable							
Test for overall effect: Not applicable							
**3.6.4 Single-dose vs. Antibiotic Cementation**							
**Subtotal (95% CI)**		**0**		**0**		**Not estimable**	
Total events	0		0				
Heterogeneity: Not applicable							
Test for overall effect: Not applicable							
**3.6.5 Multiple-dose ≤ 24hours vs Mulitple-dose > 1day**							
**Subtotal (95% CI)**		**0**		**0**		**Not estimable**	
Total events	0		0				
Heterogeneity: Not applicable							
Test for overall effect: Not applicable							
**3.6.6 Multiple-dose ≤ 24hours vs. Placebo**							
**Subtotal (95% CI)**		**0**		**0**		**Not estimable**	
Total events	0		0				
Heterogeneity: Not applicable							
Test for overall effect: Not applicable							
**3.6.7 Multiple-dose ≤ 24hours vs. Antibiotic Cementation**							
**Subtotal (95% CI)**		**0**		**0**		**Not estimable**	
Total events	0		0				
Heterogeneity: Not applicable							
Test for overall effect: Not applicable							
**3.6.8 Mulitple-dose > 1 day vs Placebo**							
**Subtotal (95% CI)**		**0**		**0**		**Not estimable**	
Total events	0		0				
Heterogeneity: Not applicable							
Test for overall effect: Not applicable							
**3.6.9 Mulitiple-dose > 1day vs Antibiotic Cementation**							
**Subtotal (95% CI)**		**0**		**0**		**Not estimable**	
Total events	0		0				
Heterogeneity: Not applicable							
Test for overall effect: Not applicable							
**3.6.10 Antibiotic Cementaiton vs Placebo**							
**Subtotal (95% CI)**		**0**		**0**		**Not estimable**	
Total events	0		0				
Heterogeneity: Not applicable							
Test for overall effect: Not applicable							

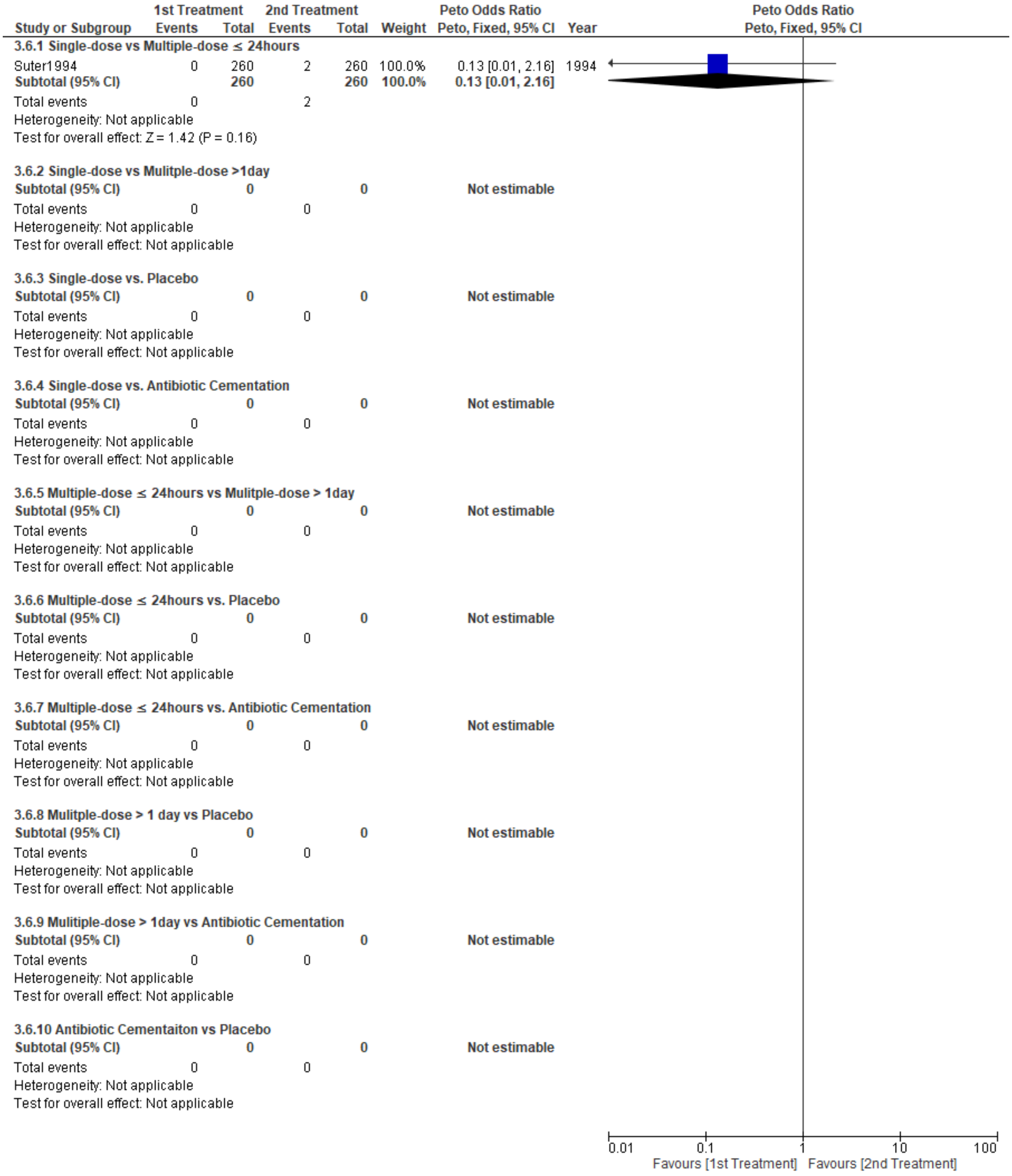

Forest plot of odds ratio on mortality comparing treatment durations

	1st Treatment		2nd Treatment			Peto Odds Ratio	
Study or Subgroup	Events	Total	Events	Total	Weight	Peto, Fixed, 95% CI	Year
**3.7.1 Single-dose vs Multiple-dose ≤ 24hours**							
Wymenga1992	47	1600	38	1599	97.6%	1.24 [0.81, 1.91]	1992
Suter1994	0	260	2	260	2.4%	0.13 [0.01, 2.16]	1994
**Subtotal (95% CI)**		**1860**		**1859**	**100.0%**	**1.18 [0.77, 1.80]**	
Total events	47		40				
Heterogeneity: Chi² = 2.40, df = 1 (P = 0.12); I² = 58%							
Test for overall effect: Z = 0.76 (P = 0.45)							
**3.7.2 Single-dose vs Mulitple-dose >1day**							
**Subtotal (95% CI)**		**0**		**0**		**Not estimable**	
Total events	0		0				
Heterogeneity: Not applicable							
Test for overall effect: Not applicable							
**3.7.3 Single-dose vs. Placebo**							
**Subtotal (95% CI)**		**0**		**0**		**Not estimable**	
Total events	0		0				
Heterogeneity: Not applicable							
Test for overall effect: Not applicable							
**3.7.4 Single-dose vs. Antibiotic Cementation**							
**Subtotal (95% CI)**		**0**		**0**		**Not estimable**	
Total events	0		0				
Heterogeneity: Not applicable							
Test for overall effect: Not applicable							
**3.7.5 Multiple-dose ≤ 24hours vs Mulitple-dose > 1day**							
**Subtotal (95% CI)**		**0**		**0**		**Not estimable**	
Total events	0		0				
Heterogeneity: Not applicable							
Test for overall effect: Not applicable							
**3.7.6 Multiple-dose ≤ 24hours vs. Placebo**							
**Subtotal (95% CI)**		**0**		**0**		**Not estimable**	
Total events	0		0				
Heterogeneity: Not applicable							
Test for overall effect: Not applicable							
**3.7.7 Multiple-dose ≤ 24hours vs. Antibiotic Cementation**							
**Subtotal (95% CI)**		**0**		**0**		**Not estimable**	
Total events	0		0				
Heterogeneity: Not applicable							
Test for overall effect: Not applicable							
**3.7.8 Mulitple-dose > 1 day vs Placebo**							
**Subtotal (95% CI)**		**0**		**0**		**Not estimable**	
Total events	0		0				
Heterogeneity: Not applicable							
Test for overall effect: Not applicable							
**3.7.9 Mulitiple-dose > 1day vs Antibiotic Cementation**							
**Subtotal (95% CI)**		**0**		**0**		**Not estimable**	
Total events	0		0				
Heterogeneity: Not applicable							
Test for overall effect: Not applicable							
**3.7.10 Antibiotic Cementaiton vs Placebo**							
**Subtotal (95% CI)**		**0**		**0**		**Not estimable**	
Total events	0		0				
Heterogeneity: Not applicable							
Test for overall effect: Not applicable							

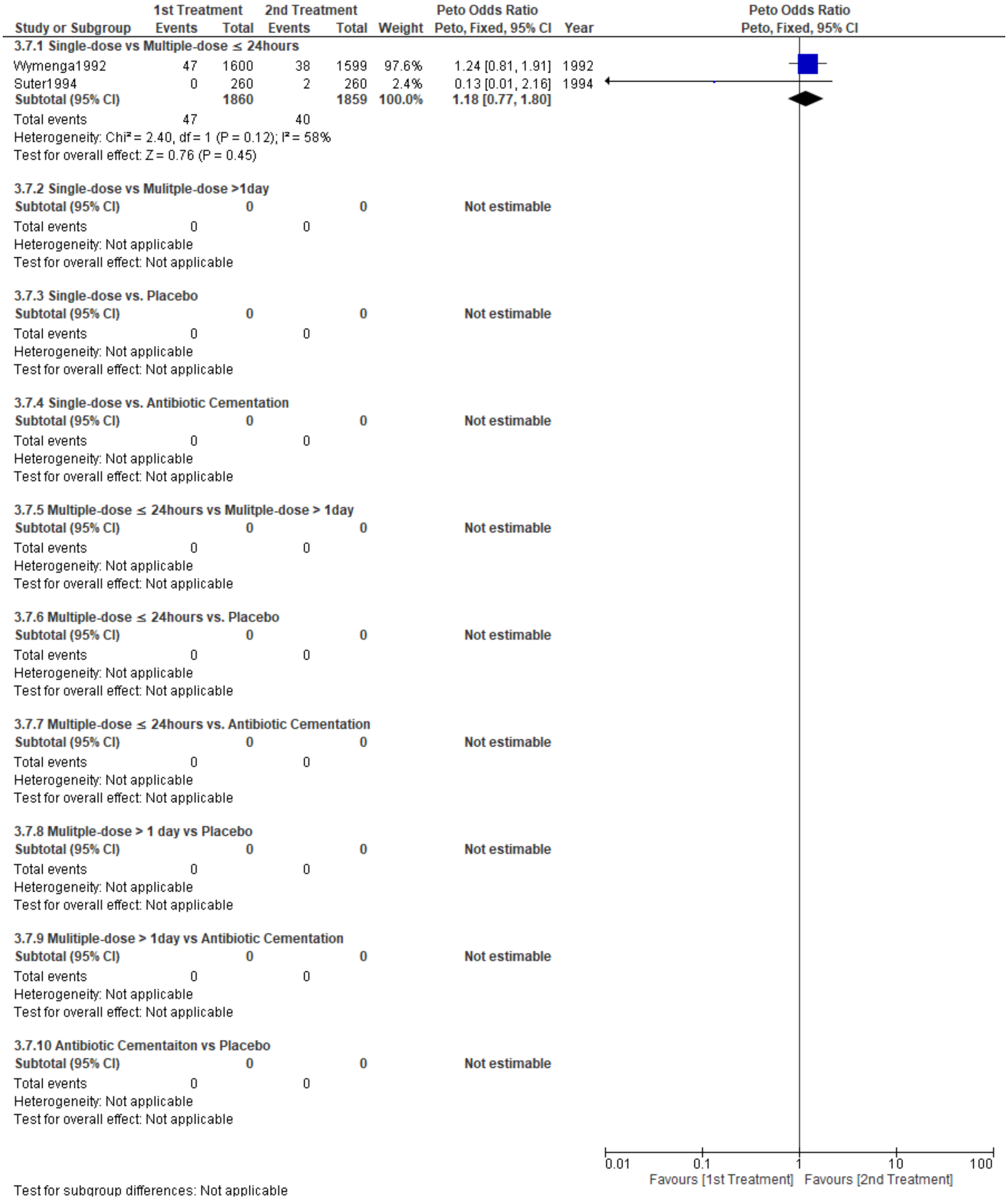

Test for subgroup differences: Not applicable

S4 Fig Forest Plots for outcomes at 90 days

Forest plot of odds ratio on serious adverse events comparing treatment durations

Study or Subgroup	1st Treatment Events	1st Treatment Total	2nd Treatment Events	2nd Treatment Total	Weight	Peto Odds Ratio Peto, Fixed, 95% CI	Year
**1.2.1 Single-dose vs Multiple-dose ≤ 24hours**							
**Subtotal (95% CI)**		**0**		**0**		**Not estimable**	
Total events	0		0				
Heterogeneity: Not applicable							
Test for overall effect: Not applicable							
**1.2.2 Single-dose vs Mulitple-dose >1day**							
**Subtotal (95% CI)**		**0**		**0**		**Not estimable**	
Total events	0		0				
Heterogeneity: Not applicable							
Test for overall effect: Not applicable							
**1.2.3 Single-dose vs. Placebo**							
**Subtotal (95% CI)**		**0**		**0**		**Not estimable**	
Total events	0		0				
Heterogeneity: Not applicable							
Test for overall effect: Not applicable							
**1.2.4 Single-dose vs. Antibiotic Cementation**							
**Subtotal (95% CI)**		**0**		**0**		**Not estimable**	
Total events	0		0				
Heterogeneity: Not applicable							
Test for overall effect: Not applicable							
**1.2.5 Multiple-dose ≤ 24hours vs Mulitple-dose > 1day**							
Mauerhan1994	1	285	0	261	100.0%	6.79 [0.13, 343.61]	1994
**Subtotal (95% CI)**		**285**		**261**	**100.0%**	**6.79 [0.13, 343.61]**	
Total events	1		0				
Heterogeneity: Not applicable							
Test for overall effect: Z = 0.96 (P = 0.34)							
**1.2.6 Multiple-dose ≤ 24hours vs. Placebo**							
Gunst1984	1	46	5	47	100.0%	0.25 [0.05, 1.29]	1984
**Subtotal (95% CI)**		**46**		**47**	**100.0%**	**0.25 [0.05, 1.29]**	
Total events	1		5				
Heterogeneity: Not applicable							
Test for overall effect: Z = 1.65 (P = 0.10)							
**1.2.7 Multiple-dose ≤ 24hours vs. Antibiotic Cementation**							
McQueen1990	1	190	2	190	100.0%	0.51 [0.05, 4.95]	1990
**Subtotal (95% CI)**		**190**		**190**	**100.0%**	**0.51 [0.05, 4.95]**	
Total events	1		2				
Heterogeneity: Not applicable							
Test for overall effect: Z = 0.58 (P = 0.56)							
**1.2.8 Mulitple-dose > 1 day vs Placebo**							
Hill1981	5	1070	16	1067	100.0%	0.35 [0.15, 0.82]	1981
**Subtotal (95% CI)**		**1070**		**1067**	**100.0%**	**0.35 [0.15, 0.82]**	
Total events	5		16				
Heterogeneity: Not applicable							
Test for overall effect: Z = 2.42 (P = 0.02)							
**1.2.9 Mulitiple-dose > 1day vs Antibiotic Cementation**							
**Subtotal (95% CI)**		**0**		**0**		**Not estimable**	
Total events	0		0				
Heterogeneity: Not applicable							
Test for overall effect: Not applicable							
**1.2.10 Antibiotic Cementaiton vs Placebo**							
**Subtotal (95% CI)**		**0**		**0**		**Not estimable**	
Total events	0		0				
Heterogeneity: Not applicable							
Test for overall effect: Not applicable							

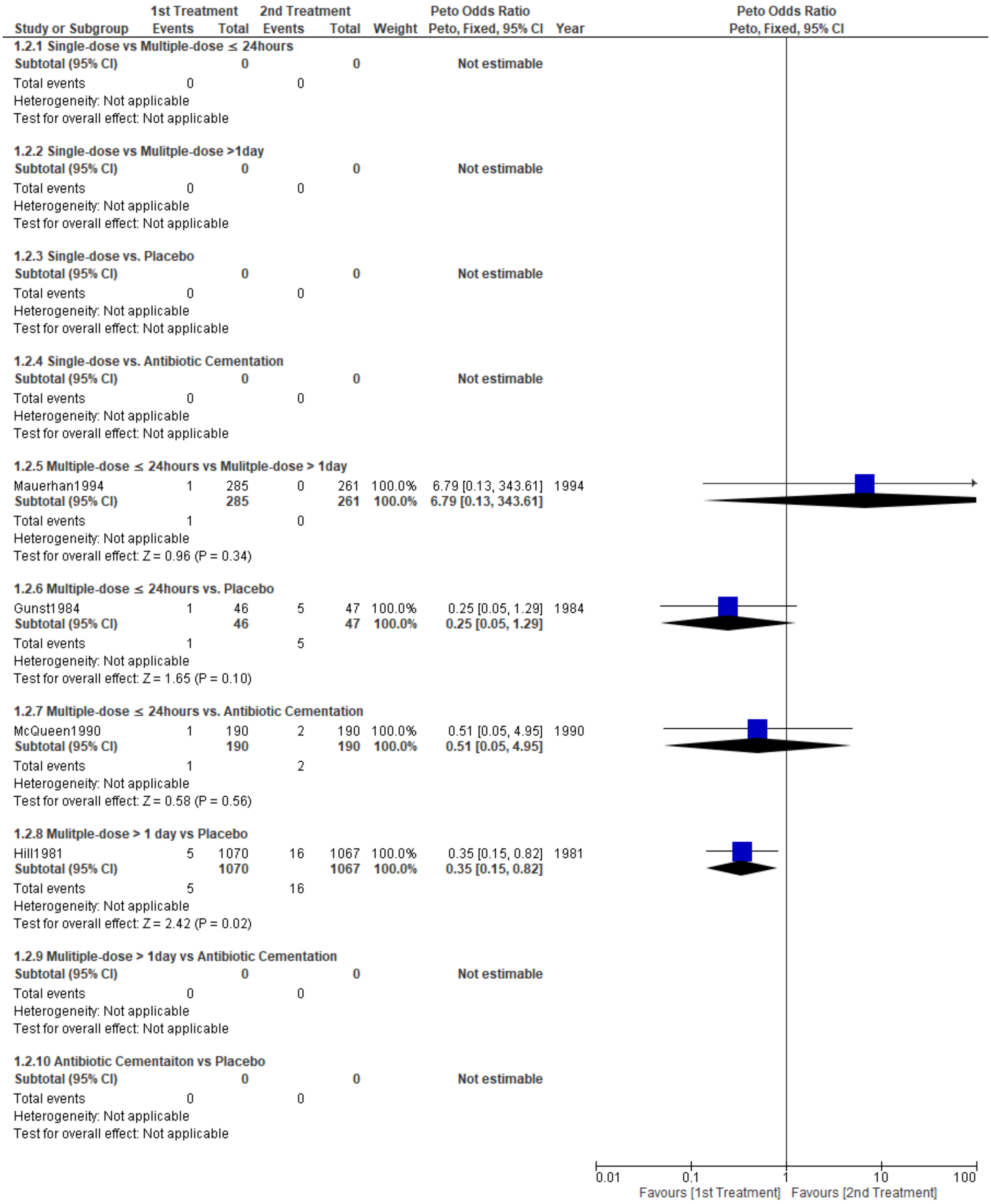

Forest plot of odds ratio on surgical site infections comparing treatment durations

Study or Subgroup	1st Treatment Events	1st Treatment Total	2nd Treatment Events	2nd Treatment Total	Weight	Peto Odds Ratio Peto, Fixed, 95% CI	Year
**1.1.1 Single-dose vs Mulitple-dose ≤24hours**							
**Subtotal (95% CI)**		**0**		**0**		**Not estimable**	
Total events	0		0				
Heterogeneity: Not applicable							
Test for overall effect: Not applicable							
**1.1.2 Single-dose vs Mulitple-dose >1day**							
**Subtotal (95% CI)**		**0**		**0**		**Not estimable**	
Total events	0		0				
Heterogeneity: Not applicable							
Test for overall effect: Not applicable							
**1.1.3 Single-dose vs. Placebo**							
**Subtotal (95% CI)**		**0**		**0**		**Not estimable**	
Total events	0		0				
Heterogeneity: Not applicable							
Test for overall effect: Not applicable							
**1.1.4 Single-dose vs. Antibiotic Cementation**							
**Subtotal (95% CI)**		**0**		**0**		**Not estimable**	
Total events	0		0				
Heterogeneity: Not applicable							
Test for overall effect: Not applicable							
**1.1.5 Multiple-dose ≤ 24hours vs Mulitple-dose > 1day**							
Mauerhan1994	4	285	2	261	100.0%	1.80 [0.36, 8.98]	1994
**Subtotal (95% CI)**		**285**		**261**	**100.0%**	**1.80 [0.36, 8.98]**	
Total events	4		2				
Heterogeneity: Not applicable							
Test for overall effect: Z = 0.71 (P = 0.48)							
**1.1.6 Multiple-dose ≤ 24hours vs. Placebo**							
Gunst1984	1	46	5	47	100.0%	0.25 [0.05, 1.29]	1984
**Subtotal (95% CI)**		**46**		**47**	**100.0%**	**0.25 [0.05, 1.29]**	
Total events	1		5				
Heterogeneity: Not applicable							
Test for overall effect: Z = 1.65 (P = 0.10)							
**1.1.7 Multiple-dose ≤ 24hours vs. Antibiotic Cementation**							
McQueen1990	9	190	16	190	100.0%	0.55 [0.24, 1.24]	1990
**Subtotal (95% CI)**		**190**		**190**	**100.0%**	**0.55 [0.24, 1.24]**	
Total events	9		16				
Heterogeneity: Not applicable							
Test for overall effect: Z = 1.45 (P = 0.15)							
**1.1.8 Mulitple-dose > 1 day vs Placebo**							
Ericsson1973	0	60	10	58	30.7%	0.11 [0.03, 0.40]	1973
Hill1981	5	1070	16	1067	69.3%	0.35 [0.15, 0.82]	1981
**Subtotal (95% CI)**		**1130**		**1125**	**100.0%**	**0.24 [0.12, 0.50]**	
Total events	5		26				
Heterogeneity: Chi² = 2.09, df = 1 (P = 0.15); I² = 52%							
Test for overall effect: Z = 3.87 (P = 0.0001)							
**1.1.9 Mulitiple-dose > 1day vs Antibiotic Cementation**							
Josefsson1981	49	834	71	851		Not estimable	1981
**Subtotal (95% CI)**		**0**		**0**		**Not estimable**	
Total events	0		0				
Heterogeneity: Not applicable							
Test for overall effect: Not applicable							
**1.1.10 Antibiotic Cementaiton vs Placebo**							
**Subtotal (95% CI)**		**0**		**0**		**Not estimable**	
Total events	0		0				
Heterogeneity: Not applicable							
Test for overall effect: Not applicable							

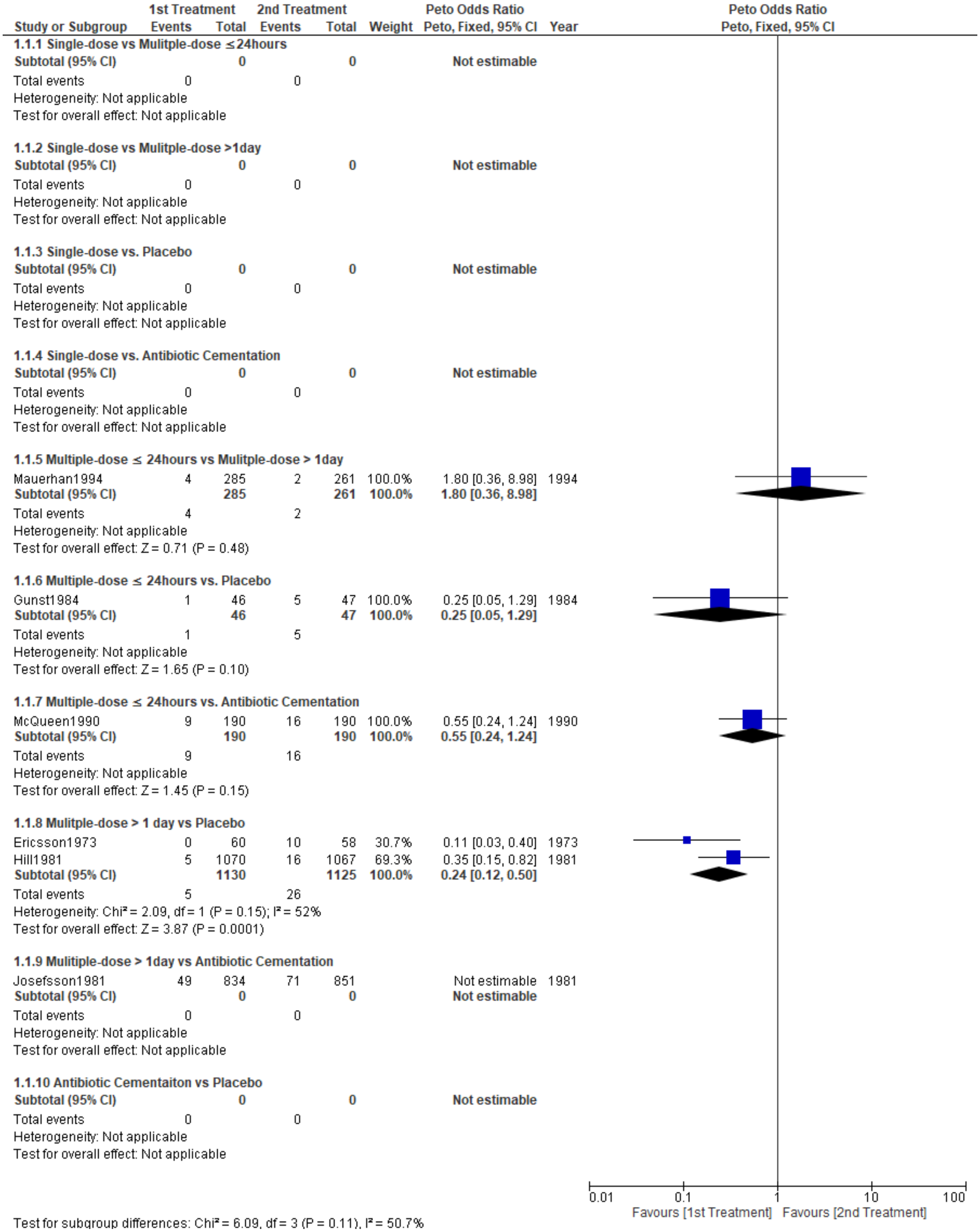

Test for subgroup differences: Chi² = 6.09, df = 3 (P = 0.11), I² = 50.7%

Forest plot of odds ratio on prosthetic joint infections comparing treatment durations

Study or Subgroup	1st Treatment Events	1st Treatment Total	2nd Treatment Events	2nd Treatment Total	Weight	Peto Odds Ratio Peto, Fixed, 95% CI	Year
**1.3.1 Single-dose vs Multiple-dose ≤ 24hours**							
**Subtotal (95% CI)**		**0**		**0**		**Not estimable**	
Total events	0		0				
Heterogeneity: Not applicable							
Test for overall effect: Not applicable							
**1.3.2 Single-dose vs Mulitple-dose >1day**							
**Subtotal (95% CI)**		**0**		**0**		**Not estimable**	
Total events	0		0				
Heterogeneity: Not applicable							
Test for overall effect: Not applicable							
**1.3.3 Single-dose vs. Placebo**							
**Subtotal (95% CI)**		**0**		**0**		**Not estimable**	
Total events	0		0				
Heterogeneity: Not applicable							
Test for overall effect: Not applicable							
**1.3.4 Single-dose vs. Antibiotic Cementation**							
**Subtotal (95% CI)**		**0**		**0**		**Not estimable**	
Total events	0		0				
Heterogeneity: Not applicable							
Test for overall effect: Not applicable							
**1.3.5 Multiple-dose ≤ 24hours vs Mulitple-dose > 1day**							
Mauerhan1994	1	285	1	261	100.0%	0.92 [0.06, 14.71]	1994
**Subtotal (95% CI)**		**285**		**261**	**100.0%**	**0.92 [0.06, 14.71]**	
Total events	1		1				
Heterogeneity: Not applicable							
Test for overall effect: Z = 0.06 (P = 0.95)							
**1.3.6 Multiple-dose ≤ 24hours vs. Placebo**							
Gunst1984	1	46	8	47	100.0%	0.19 [0.05, 0.73]	1984
**Subtotal (95% CI)**		**46**		**47**	**100.0%**	**0.19 [0.05, 0.73]**	
Total events	1		8				
Heterogeneity: Not applicable							
Test for overall effect: Z = 2.41 (P = 0.02)							
**1.3.7 Multiple-dose ≤ 24hours vs. Antibiotic Cementation**							
McQueen1990	1	190	2	190	100.0%	0.51 [0.05, 4.95]	1990
**Subtotal (95% CI)**		**190**		**190**	**100.0%**	**0.51 [0.05, 4.95]**	
Total events	1		2				
Heterogeneity: Not applicable							
Test for overall effect: Z = 0.58 (P = 0.56)							
**1.3.8 Mulitple-dose > 1 day vs Placebo**							
Hill1981	84	1070	154	1067	100.0%	0.51 [0.39, 0.67]	1981
**Subtotal (95% CI)**		**1070**		**1067**	**100.0%**	**0.51 [0.39, 0.67]**	
Total events	84		154				
Heterogeneity: Not applicable							
Test for overall effect: Z = 4.84 (P < 0.00001)							
**1.3.9 Mulitiple-dose > 1day vs Antibiotic Cementation**							
**Subtotal (95% CI)**		**0**		**0**		**Not estimable**	
Total events	0		0				
Heterogeneity: Not applicable							
Test for overall effect: Not applicable							
**1.3.10 Antibiotic Cementaiton vs Placebo**							
**Subtotal (95% CI)**		**0**		**0**		**Not estimable**	
Total events	0		0				
Heterogeneity: Not applicable							
Test for overall effect: Not applicable							

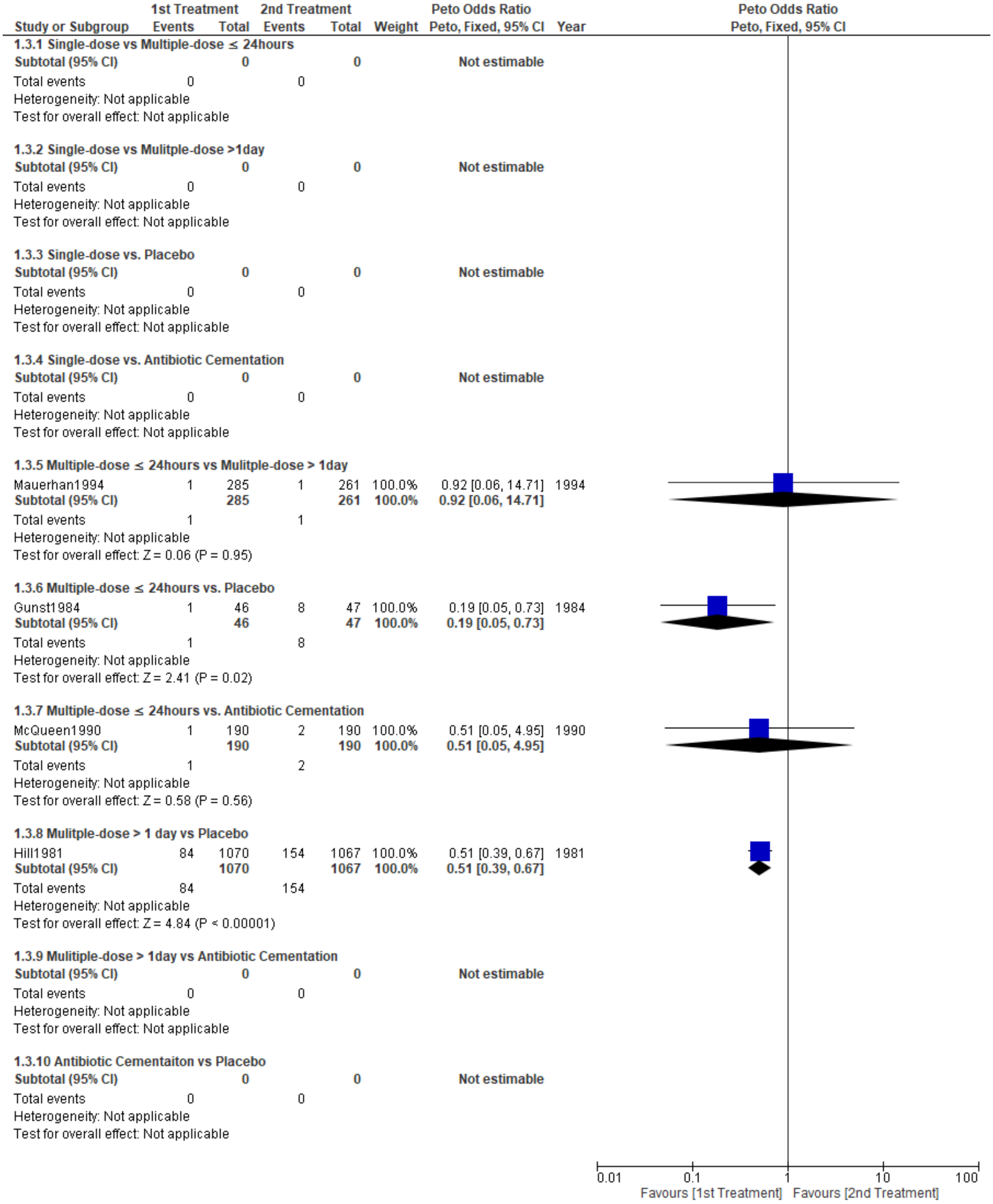

Forest plot of odds ratio on serious infections comparing treatment durations

Due to insufficient data, it is not possible to provide estimates of effects and quality ratings for the comparison of treatment durations for the outcome of serious infections within 90 after surgery.

Forest plot of odds ratio on major cardiovascular events comparing treatment durations

Due to insufficient data, it is not possible to provide estimates of effects and quality ratings for the comparison of treatment durations for the outcome of major cardiovascular events within 90 after surgery.

Forest plot of odds ratio on venous thromboembolisms comparing treatment durations

Due to insufficient data, it is not possible to provide estimates of effects and quality ratings for the comparison of treatment durations for the outcome of venous thromboembolisms within 90 after surgery.

Forest plot of odds ratio on mortality comparing treatment durations

Due to insufficient data, it is not possible to provide estimates of effects and quality ratings for the comparison of treatment durations for the outcome of mortality within 90 after surgery.

S5 Figure. Point estimates and 95% confidence intervals

Each treatment is compared against placebo, chosen as the constant comparator.

This visual representation facilitates the following appropriate inferences: (i) Point estimates suggest that all treatments are superior to placebo; (ii) any true differences between antibiotic cementation, single-dose, multiple-doses within 24 hours and multiple-doses more than 1 day are likely to be small; (iii) the confidence intervals for one treatment only slightly overlaps (multiple-doses more than 1 day), thus having only minimal impact on our certainty about inference (i) and (ii).

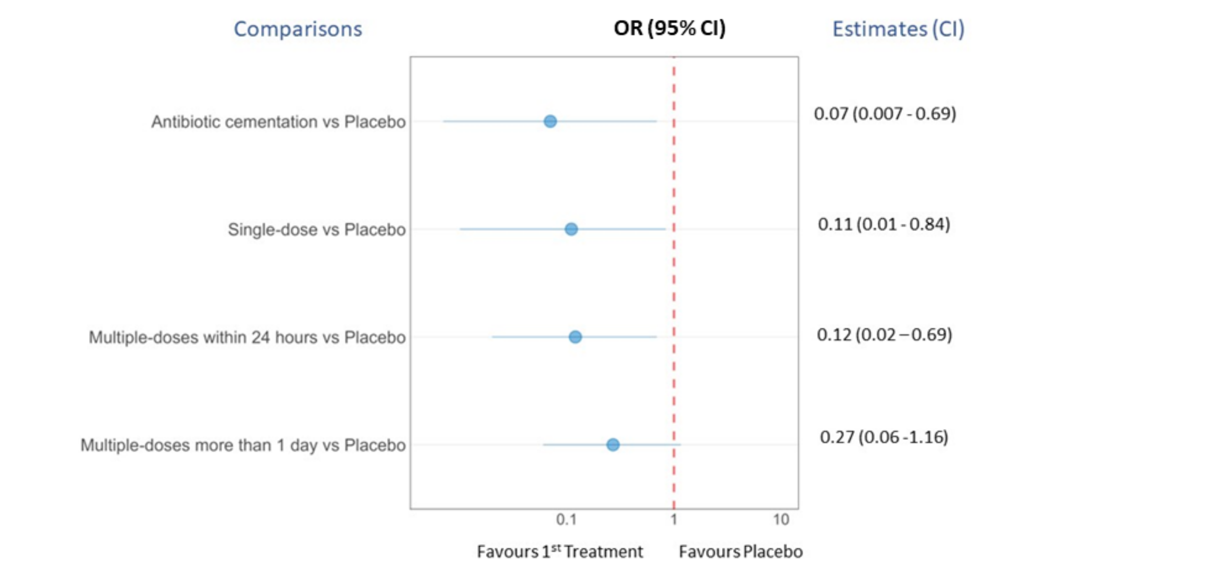

Point estimates and confidence intervals of comparisons between the 4 dosage practices and placebo

Table S7. Outcomes and Outcome Definitions Reported in Eligible Trials

Study	Outcomes	Outcome definition
Hill et al 1981[1]<br>5 days of cefazolin vs placebo	PJI<br>Serious Infections	PJI: Patients were recalled six months, twelve months and two years after operation. The condition of the hip was evaluated clinically, radiologically and biologically (erythrocyte sedimentation rate). Hip infection was defined as clinical infection in the hip (abscess, septicaemia, or lethal infection).<br><br>Serious infections: defined as septic complications including urinary, pulmonary and digestive infections.
Gunst et al 1984[2]<br>1 day of Cefamandole vs placebo	PJI	Clinical signs and positive culture<br><br>Patients evaluated clinically and radiologically systematically at 6 weeks, 3 months and 12 months postoperatively.<br><br>Serious complications defined as infections at the level of the prosthesis as a new surgical approach to the joint.<br><br>Early infections defined by the rapid appearance of infectious phenomena at the level of the prosthesis and reoperation within 1 month or delayed/late onset (longer than 1 month) of signs of deep infection, pain corresponding to the prosthesis and radiological signs of loosening.
Ericson et al 1973[3]	PJI	Clinical signs and positive culture<br><br>PJI: If the patient showed any clinical signs and if a culture on one test gave growth of either potentially pathogenic bacteria or doubtful cases on more than one culture
Josefsson et al 1981[4]<br><br>7-14 days of systemic antibiotic vs gentamicin cement	SSI<br>PJI	Superficial: abnormal redness of wound, presence of secretion and firm diagnosis. Deep: pain, elevated ESR,*a* progressive radiographic resorption of bone stock<br><br>Superficial surgical site infection: abnormal redness of the wound, presence of secretion, and the fact that the diagnosis had been so firm that antibiotic treatment had been instituted.<br><br>PJI: A diagnosis of deep infection was based on the following three criteria: pain, elevated ESR (more than 35 mm per hour) and progressive radiographic resorption of bone stock. Four of the participating clinics had no facilities for the advanced bacteriologic investigations (e.g., anaerobic culture) necessary for a reliable bacteriologic diagnosis in loosened THA. A positive bacterial finding in the revised cases, therefore, could not be set as an absolute criterion for deep infection.
Mauerhan et al 1994 [5]<br>1 day of cefuroxime vs 3 days of cefazolin	SSI<br>PJI	Wound infections were classified as superficial or deep, depending on whether they had developed above or below the fascia.<br><br>PJI: positive culture of purulent drainage from inflamed wound.
Centulio et al 1988 [6]	SSI<br>PJI	Infections described as either superficial or deep, no further elaboration apart from description of pathogen in case of culture.

Single-dose ceftriaxone vs 3 days ceftriaxone		All patients included in the study were controlled with new hospitalizations or with periodic outpatient visits every 3 months in order to be able to exclude late infections.
McQueen et al 1987 [7]	SSI<br>PJI	Superficial infection: Infection superficial to the deep fascia with positive or negative bacteriological cultures and no delay in wound healing<br><br>Deep infection Infection: extending deep to the deep fascia, with persistent wound discharge or joint pain, positive or negative cultures from deep tissues and delay in wound healing.<br><br>Diagnosed by the presence of two or more of the following: Pain in or around joint, at rest or on movement ESR > 30 mm/hr above preoperative level<br>Pathogenic organisms from joint aspirates<br>Radiological evidence of infection, such as pefiosteal reaction or bone resorption<br>A persistent sinus in communication with the joint<br><br>Furthermore, classified as early (within 3 months) or late (3 months to 2 years).
Pollard et al 1979[8]	SSI<br>PJI	Superficial: Purulent discharge,with or without pyrexia.<br><br>Deep: pain, fever, redness with discharge containing pathogenic organism, elevated ESR, progressive radiographic resorption of bone stock.<br><br>Superficial infections were considered to be minor when there was a purulent discharge without pyrexia, moderate when there was a discharge accompanied by pyrexia, and severe when there was a major wound dehiscence.<br><br>Deep infection was considered to be either early or late-early when it occurred before six months and late thereafter.<br>Early infection was diagnosed by the presence of pain, fever, redness of the wound, and a discharge containing pathogenic organisms or many polymorphonuclear leucocytes.<br><br>Late infection was diagnosed by the presence of two or more of the following criteria: pain in the hip; a discharging sinus; isolation of pathogenic organisms from a sinus or by direct aspiration; isolation of material from which no organisms could be cultured and which contained many polymorphonuclear leucocytes; an erythrocyte sedimentation rate (ESR) that was raised above the preoperative level by 30 mm or more in the first hour; or radiological evidence of infection such as periosteal reaction, bone reabsorption, or irregular reabsorption of the calcar.
Suter et al 1994 [9]	SSI<br>PJI<br>VTE<br>Mortality	The primary parameter of efficacy was the occurrence of deep infection or infection of the prosthetic device, characterized by pain, local tenderness, abnormal erythrocyte sedimentation rate, radiographic signs of infection or positive bacterial cultures of the periprosthetic space.<br><br>Secondary parameters of efficacy were wound complications, defined as erythema, serous exudate with negative culture, superficial haematoma with negative cultures, purulent or culture-positive serous exudate and superficial haematoma with positive

		cultures. The last two lesions were considered infective complications of the wound.<br><br>VTE: one case with massive pulmonary embolism<br><br>Infections of other body sites were also recorded: respiratory tract infections (clinical signs of infection or production of mucous or purulent sputum and radiological signs of infection) and urinary tract infections (clinical signs and symptoms confirmed by at least one positive (> 105 cfu/ml) culture of clean-catch midstream urine). Finally, febrile morbidity, expressed as axillary body temperature of > 37.5 °C for two or more days, excluding the day of surgery, was monitored. – febrile morbidity unspecified whether serious infection
Wymenga et al 1992 [10]	SSI<br>PJI<br>Serious infections<br>Mortality	PJI: Positive culture, evidence of sepsis, erythema.<br><br>The clinical end-point of the study was joint sepsis, reoperation or death.<br><br>Confirmed joint sepsis was defined as a positive bacteriologic culture at reoperation or a draining sinus. Strong evidence of sepsis was defined as four or more possible signs of infection. These two groups of conditions were analyzed together (Category I). In patients who only showed two or three possible signs of sepsis (Category II), a definite diagnosis could not be made. Patients with one or no signs of infections (Category III) were not suspected of having joint sepsis. The conditions that were defined as being possible infections at the follow-up examination were pain during weight bearing and/or at rest, tenderness of the wound, fever, an abnormal radiograph, ESR more than 35mm, positive culture from joint fluid aspirate, positive arthrogram, bone scan showing typical signs of infection, or increased C-reactive protein.<br>Wound infection in the postoperative period was defined as erythema more than 1 cm from the incision.<br><br>Superficial surgical site infection: Minor postoperative would-healing problems were defined as erythema more than 1 cm from the incision, pus suture, small wound dehiscence, necrosis of the would edge, and blisters.<br><br>Distant infections included pulmonary, urinary, skin and septicaemia.<br><br>Serious infections: of the reported distant infections; only septicemia was considered for this outcome.

SSI: Surgical site infection, PJI: prosthetic joint infection, VTE: venous thromboembolism.

1. Hill C, Flamant R, Mazas F, Evrard J. Prophylactic cefazolin versus placebo in total hip replacement. Report of a multicentre double-blind randomised trial. Lancet. 1981;1(8224):795-6.
2. Gunst JP, Deletang S, Rogez JM. Prophylactic antibiotic therapy by cefamandole in total hip replacement with use of a clean air system. A randomized study. [French]. Pathologie Biologie. 1984;32(5 BIS):567-9.

3. Ericson C, Lidgren L, Lindberg L. Cloxacillin in the prophylaxis of postoperative infections of the hip. J Bone Joint Surg Am. 1973;55(4):808-13, 43.
4. Josefsson G, Lindberg L, Wiklander B. Systemic antibiotics and gentamicin-containing bone cement in the prophylaxis of postoperative infections in total hip arthroplasty. Clin Orthop Relat Res. 1981(159):194-200.
5. Mauerhan DR, Nelson CL, Smith DL, Fitzgerald RH, Jr., Slama TG, Petty RW, et al. Prophylaxis against infection in total joint arthroplasty. One day of cefuroxime compared with three days of cefazolin. J Bone Joint Surg Am. 1994;76(1):39-45.
6. Centulio F, Conticello A. Antimicrobial chemoprophylaxis with ceftriaxone in surgical implantation of a non-cemented hip prosthesis: comparison of 2 dosage schemes. [Italian]. La Chirurgia degli organi di movimento. 1988;73(4):357-61.
7. McQueen M, Littlejohn A, Hughes SP. A comparison of systemic cefuroxime and cefuroxime loaded bone cement in the prevention of early infection after total joint replacement. Int Orthop. 1987;11(3):241-3.
8. Pollard JP, Hughes SP, Scott JE, Evans MJ, Benson MK. Antibiotic prophylaxis in total hip replacement. Br Med J. 1979;1(6165):707-9.
9. Suter F, Avai A, Fusco U, Gerundini M, Caprioli S, Maggiolo F. Teicoplanin versus cefamandole in the prevention of infection in total hip replacement. Eur J Clin Microbiol Infect Dis. 1994;13(10):793-6.
10. Wymenga A, van Horn J, Theeuwes A, Muytjens H, Slooff T. Cefuroxime for prevention of postoperative coxitis. One versus three doses tested in a randomized multicenter study of 2,651 arthroplasties. Acta Orthop Scand. 1992;63(1):19-24.

S8 Table Sensitivity Analysis, excluding the node: antibiotic-loaded bone cement

Table 2.11 Sensitvity Analysis: Estimates of effects for comparison of antibiotic prophylaxis for prevention of SAEs 365 days after primary THA

Comparison	Network meta-analysis<br>Odds Ratio (95% CI)
Single-dose vs Multiple-dose ≤24hours	1.19 (0.37 to 3.73)
Single-dose vs Multiple-dose >1day	0.76 (0.20 to 2.84)
Single-dose vs. Placebo	0.16 (0.03 to 0.74)
Multiple dose ≤24hours vs Multiple-dose >1day	0.64 (0.21 to 1.92)
Multiple-dose ≤24hours vs Placebo	0.12 (0.03 to 0.51)
Multiple-dose >1day vs Placebo	0.21 (0.06 to 0.69)

Table 2.12 Sensitvity Analysis: Estimates of effects for comparison of antibiotic prophylaxis for prevention of SSIs 365 days after primary THA

Comparison	Network meta-analysis<br>Odds Ratio (95% CI)
Single-dose vs Multiple-dose ≤24hours	0.93 (0.63 to 1.38)
Single-dose vs Multiple-dose >1day	0.95 (0.43 to 2.01)
Single-dose vs. Placebo	0.19 (0.07 to 0.50)
Multiple dose ≤24hours vs Multiple-dose >1day	1.02 (0.49 to 2.10)
Multiple-dose ≤24hours vs Placebo	0.21 (0.08 to 0.51)
Multiple-dose >1day vs Placebo	0.20 (0.10 to 0.43)

Table 2.13 Sensitvity Analysis: Estimates of effects for comparison of antibiotic prophylaxis for prevention of SAEs 90 days after primary THA	
	Network meta-analysis
Comparison	Odds Ratio (95% CI)
Multiple dose ≤24hours vs Multiple-dose >1day	1.46 (0.17 to 12.2)
Multiple-dose ≤24hours vs Placebo	0.24 (0.03 to 1.60)
Multiple-dose >1day vs Placebo	0.16 (0.03 to 0.84)

Table 2.13 Sensitvity Analysis: Estimates of effects for comparison of antibiotic prophylaxis for prevention of SSIs 90 days after primary THA	
	Network meta-analysis
Comparison	Odds Ratio (95% CI)
Multiple dose ≤24hours vs Multiple-dose >1day	1.71 (0.36 to 7.95)
Multiple-dose ≤24hours vs Placebo	0.28 (0.06 to 1.37)
Multiple-dose >1day vs Placebo	0.17 (0.04 to 0.61)

Comparative Effectiveness on the Use of Antibiotic Prophylaxis and Serious Adverse Events Following Total Hip Arthroplasty: Protocol for a Systematic Review and Network Meta-Analysis of Randomized Trials

Registration: The study was submitted for registration in PROSPERO database of systematic reviews on **20.12.22**

Collaborators/Authors: Armita Armina Abedi,[1,2,3] Håkan Jacob Moflag Svensson,[1,2] Alma Becic Pedersen[4,5], Claus Varnum,[6,7] Sabrina M. Nielsen,[3,8] Robin Christensen,[3,8] Søren Overgaard[1,2]

Affiliations

1: Department of Orthopedic Surgery and Traumatology, Copenhagen University Hospital, Bispebjerg, Denmark.

2: Department of Clinical Medicine, Faculty of Health and Medical Sciences, Unive
rsity of Copenhagen, Copenhagen, Denmark.

3: Section for Biostatistics and Evidence-Based Research, the Parker Institute, Bispebjerg and Frederiksberg Hospital, Copenhagen, Denmark.

4: Department of Clinical Epidemiology, Aarhus University Hospital, Aarhus, Denmark.

5: Department of Clinical Medicine, Aarhus University, Aarhus, Denmark.

6: Department of Orthopedics, Lillebaelt Hospital, Vejle, Denmark.

7: Department of Regional Health Research, University of Southern Denmark, Odense, Denmark.

8: Research Unit of Rheumatology, Department of Clinical Research, University of Southern Denmark, Odense University Hospital, Denmark.

CONTRIBUTIONS

All authors contributed to the making of the protocol.
AAA and HJMS will perform the study selection and data extraction.

AAA will be responsible for writing and submitting the manuscript. All authors will review the manuscript before submission.

Contact details for further information

Armita Armina Abedi: armita.armina.abedi@regionh.dk

Support

There is no financial or other support explicitly donated for this review. Section for Biostatistics and Evidence-Based Research, The Parker Institute, Bispebjerg and Frederiksberg Hospital is supported by a core grant from the Oak Foundation (OCAY-18-774-OFIL).

SUMMARY

Introduction: A feared complication after total hip arthroplasty (THA) is surgical site infection (SSI). SSI involving the joint prosthesis and adjacent tissue is known as periprosthetic joint infection (PJI). PJI is associated with high morbidity and increased mortality. Optimal use of antibiotics is one of the main modifiable factors for the prevention of SSI's. However, there is no consensus on the recommended dosages of antibiotic prophylaxis and current guidelines are based on low evidence level. From a review of the literature, three meta-analyses have been published evaluating antibiotic prophylaxis practices in THA arriving at no definite overall conclusion on the efficacy of the different practices in the prevention of SSI and other important serious adverse events (SAEs).

The primary objective of this study will be to use network meta-analysis to compare the relative effectiveness of the different antibiotic prophylaxis approaches in the prevention of SAEs in patients after THA. Secondly, the objective will be to compare occurrence of SSI and PJI and the following sub-components of the SAEs: any serious infections (except SSI and PJI), major cardiovascular events, venous thromboembolisms and mortality across the different antibiotic prophylaxis approaches.

Methods and analyses: This systematic review and network meta-analysis will include randomized trials comparing single-dose administration of antibiotic prophylaxis versus continued coverage with multiple postoperative doses of prophylactic antibiotics within and beyond 24 hours versus placebo or no prophylactic antibiotic use. Adult patients age ≥18 years enrolled in trials, and receiving primary THA for any reason will be considered eligible. The major outcome will be SAEs, additional outcomes include surgical site infections (SSI and PJI), any other serious infections, major cardiovascular events (MACE), venous thromboembolic complications (VTE) and mortality. The outcomes will be prioritized as those evaluated within 90 days and secondarily up to 1 year after the primary THA, where applicable. Network meta-analyses will be conducted to generate Odds Ratio (OR) estimates with 95% confidence intervals of comparative effectiveness of each intervention class and rankings of their effectiveness, in terms of reduction of SAEs in general and SSIs and PJIs, any other serious infections, MACE, VTEs and mortality. The certainty of evidence in the network meta-analysis will be evaluated using the Confidence in Network Meta-Analysis (CINeMA) tool.

Interpretation: To our knowledge, this will be the first network meta-analysis with a focus on comparative effectiveness on whether there exists a superior – and thus, likely preferable - approach based on all the

antibiotic prophylaxis practices in THA exclusively. We anticipate that the findings from this study will provide valuable knowledge that will potentially change future clinical practice with regards to prophylactic antibiotics for THA.

INTRODUCTION

Total Hip Arthroplasty (THA) is one of the most common orthopedic procedures worldwide with over 600,000 performed in Europe and the United States annually (4-6). Postoperative surgical-site infection (SSI) is a serious complication of THA and SSI involving the joint prosthesis and adjacent tissue is known as periprosthetic joint infection (PJI). SSI is associated with high morbidity and increased mortality (7-11). Perioperative antimicrobial prophylaxis is a well-established and documented part of standard care to reduce the risk of SSI after THA (3, 12-15). However, there is no consensus regarding perioperative dosage and current recommendations on antibiotic dosing are based on low-level evidence (16-18).

Recent guidelines from the U.S. Centers for Disease Control and Prevention (CDC) (2017) and the World Health Organization (WHO) advocate for the use of one single pre-operative dose of prophylactic antibiotic (19, 20). However, several joint guidelines recommend up to 24 hours of antimicrobial prophylaxis (21-23).

Three meta-analyses have previously evaluated antibiotic prophylaxis practices in THA with focus on the prevention of SSI and PJI and found no superiority of postoperative antibiotic prophylaxis practices (16-18). Furthermore, one study concluded (18) that with regards to prophylactic antibiotics, neither timing, route of administration, nor concentration affects the rate of revision, adverse events, or costs following THA. A recent observational register-based study suggests that a single-dose may be non-inferior to multiple-doses of prophylactic antibiotics in preventing PJI following THA (24). There may be several potential benefits in reducing the use of antibiotics including reduced risk of opportunistic infections such as pseudomonas colitis and the potential for systemic toxicity (25-28). However, based on the aforementioned studies, it still remains unknown, whether prophylactic antibiotic practices may differ with regards to prevention of several important potential SAEs.

One important and potential SAE following THA is serious infection including healthcare-associated infections such as postoperative urinary tract infection (UTI) and respiratory tract infection. These infections may develop into serious infections and septic shock, prolonging hospital stays and increasing mortality (29-31). Theoretically, a longer duration of antibiotic prophylaxis may be effective in both prevention and development of healthcare-associated infections however, this association is not well-established.

Other potential SAEs following THA are Major cardiovascular events (MACE) including myocardial infarction (MI), stroke, and cardiac arrest (32-35). There exists an association between infections including respiratory infections, urinary infections, skin infections, and blood infections and the risk of MI and stroke (36-38). This may be explained by the inflammatory response triggered by infection, which boosts atherosclerosis and prompts plaque rupture (39). In addition, immobilization following infection may also contribute to an increased cardiovascular risk. Venous thromboembolism (VTE) is another potential serious adverse event following THA (33). As a response to severe infection, an interaction between coagulation and inflammation may lead to hemostatic abnormalities and risk of a thromboembolic event (40, 41).

Taken together, antibiotic prophylaxis practices may be effective in the prevention of several SAEs following THA and there may be a difference in outcome, depending on the choice of antibiotic prophylaxis practice. Generally, shorter antibiotic prophylaxis practices should be encouraged given the potential for resistance, systemic toxicity (25-28), and costs of prolonged use of antibiotics if the effectiveness is comparable to antibiotic practices of longer duration.

This will be the first study, based on evidence synthesis and with a focus on comparative effectiveness in SAE prevention, to examine whether there exists a difference in approach based on all the antibiotic prophylaxis practices in THA. The findings of our study coupled with reports on the rise in antibiotic resistance may add valuable knowledge to change future clinical practice of prophylactic antibiotic practice for THA.

Objectives

The primary objective of this study will be to use network meta-analysis in order to use both direct and indirect evidence to compare the relative effectiveness of the different antibiotic prophylaxis approaches in the prevention of SAEs in patients after THA. Secondly, the objective will be to compare occurrence of SSI and PJI and the following sub-components of the SAEs: any serious infections, MACE, VTEs and mortality across the different antibiotic prophylaxis practices.

METHODS

This protocol was developed in accordance with the preferred reporting items for systematic reviews and meta-analyses protocols (PRISMA-P) statement (42). It will be registered in the International Prospective Register of Systematic Reviews (PROSPERO) to pre-specify the objectives and methods of the systematic review; the protocol was submitted to PROSPERO on 2022-12-20. The study report will follow the PRISMA extension for network meta-analyses (43).

Eligibility criteria

This systematic review will include Randomized Controlled Trials (RCTs) with no restriction to language (if translatable) and no restriction with regards to publication year. Trials will be eligible for inclusion if they include any antibiotic prophylaxis in adult patients of age ≥ 18 years, receiving primary THA. We will not include trials with a follow-up period of less than 90 days (i.e., ≥3 months).

Participants

Adult patients of age ≥ 18 years, receiving primary THA for all indications will be considered potentially eligible.

Interventions and Comparators

The anticipated intervention groups to be included in the network are single-dose administration of antibiotic prophylaxis, and continued coverage with multiple postoperative doses of prophylactic antibiotics within and beyond 24 hours. Other relevant antibiotic prophylaxis treatments may be evaluated in the eligible RCTs as well. The groups will be split or merged where reasonable, considering antibiotic classes and doses including frequency, duration, and route of administration.

The anticipated comparisons to be included in the network are any of the described treatment interventions compared with each other, placebo or no prophylactic antibiotic use.

The nodes to be included in the network meta-analysis will be specified prior to conducting any analyses.

Outcomes

The main outcome will be the number of patients experiencing an SAE after their THA with subsequent focus on the additional outcomes SSI and PJI, any serious infection, MACE, VTE and Mortality. The follow-up period for all outcomes is set to ≥90 days after primary THA, outcomes will be evaluated within 90 days or 1 year, where applicable.

SAEs will be assessed according to the International Conference on Harmonization of Technical Requirements for Registration of Pharmaceuticals for Human Use, document E6R (44). SAE refers to an event involving a significant risk of death or disability of the patient (or their offspring), including, but not limited to, an event that: results in death, is life-threatening – in the investigator's opinion the patient was in immediate risk of death from the adverse event when it appeared, requires hospitalization, or prolongs existing hospitalization, results in permanent or significant disability or is a congenital anomaly.

SSIs are defined according to the widely accepted Center for Disease control and Prevention (CDC) criteria as either superficial (restricted to the skin or subcutaneous tissue), deep (involving the muscle or fascia layers), or organ-space (involving the internal anatomic region where the operation was performed) (45). SSI involving the joint prosthesis and adjacent tissue is defined as periprosthetic joint infection (PJI). The current CDC definition of SSI was first published in 1992 and therefore, alternative, or previous definitions of SSI, may have been applied to a larger extent throughout the 1980s and 1990s. There is evidence that either systems to define SSI may provide important information comparable to those attained by the CDC criteria (46). We wish to consider all available evidence in the evidence syntheses and therefore SSI will be the outcome of interest as defined by each study.

Serious infections are defined as any infections associated with admission to hospital or hospital-acquired infection or hospital-treated infection or death due to infection.

MACE is defined as a composite end point of myocardial infarction, cerebrovascular accident, or cardiovascular death (47-49).

VTE is defined as a composite end point of deep venous thrombosis (DVT) or pulmonary embolism.

Mortality is defined as any death occurring after primary THA.

Data sources

The following databases will be searched systematically for eligible studies

1. Medline via Pubmed from 1946
2. EMBASE via Ovid from 1974
3. Cochrane Central Register of Controlled Trials (CENTRAL, The Cochrane Library)
4. Web of Science via Web of Knowledge from 1900
5. ClinicalTrials.gov

6. World Health Organization International Clinical Trial Registry Platform portal (ICTRP)

Furthermore, to identify any additional studies not retrieved by the search strategies, a manual search of the reference lists of the included articles and any relevant reviews identified with guidance from a research librarian, will also be performed.

Search String (applicable for PubMed)

The search string will include terms relating to or describing the population and intervention and the database specific filters suggested by Cochrane for identifying randomized trials.

1. Population

THA[tw] OR THR[tw] OR Arthroplasty, Replacement, Hip[MeSH] **OR ((**Hip[tw] OR Joint[tw]) **AND** (Replace*[tw] OR prosthes*[tw] OR Implant*[tw] OR Arthroplast*[tw] OR Surger*[tw]))

2. Intervention

(antibiotic*[tw] OR antibacterial*[tw] OR ("anti-bacterial"[tw] AND "agents"[tw]) OR "anti-bacterial agents"[tw] OR ("anti"[tw] AND "bacterial"[tw] AND "agents"[tw]) OR "anti bacterial agents"[tw]) OR aminoglycosid*[tw] OR cephalosporin*[tw] OR cefazolin[tw] OR cefepime[tw] OR cefuroxime[tw] OR ciprofloxacin[tw] OR vancomycin[tw] OR aztreonam*[tw] OR levaquin[tw] OR trimethoprim[tw] OR linezolid[tw] OR oxazolidinone*[tw] OR ofloxacin*[tw] "Anti-Bacterial Agents"[MeSH Terms] OR "Anti-Bacterial Agents"[Pharmacological Action] OR "Antibiotic Prophylaxis"[MeSH Terms] OR "Aminoglycosides"[Mesh] OR "Vancomycin"[MeSH Terms] OR "Cephalosporins"[MeSH Terms] OR "Ciprofloxacin"[MeSH Terms] OR "Ofloxacin"[MeSH Terms] OR "Aztreonam"[MeSH Terms] OR "trimethoprim, sulfamethoxazole drug combination"[MeSH Terms] OR "Oxazolidinones"[MeSH Terms]

3. *RCT filter from Cochrane: Sensitivity-maximizing version (2008 revision)*; PubMed format (1)

(randomized controlled trial[pt] OR controlled clinical trial[pt] OR randomized[tiab] OR placebo[tiab] OR drug therapy[sh] OR randomly[tiab] OR trial[tiab] OR groups[tiab] NOT (animals [mh] NOT humans [mh]))

3. #1 AND #2 AND # 3

Study records

Data management: All identified citations will be imported to Covidence, an online software for study screening developed by the Cochrane group.

Selection Process: To maintain accuracy, two review authors (AAA and HJMS) will independently screen all titles and abstracts yielded by the search against the inclusion criteria. The selection process will be presented in a flow diagram. Full reports will be obtained for all citations that appear to meet the inclusion criteria or where this cannot be excluded. AAA and HJMS will then screen full texts of the remaining reports and decide on their eligibility. Disagreement will be resolved by discussion, and if any diagreements cannot be resolved by consensus, a third reviews will arbitrate the final decision. Reasons for excluding full texts will be recorded.

Data extraction: Data from the included studies will be extracted into standardized pre-determined tables:

(1) Publication traits (study ID, year, setting/country, and funding source)

(2) Study characteristics (e.g., trial duration, intervention type, comparator type, treatment regimen for each arm, number of participants randomized [total and for each arm], inclusion/exclusion criteria, follow-up period). Descriptions of interventions and comparators include: of antibiotic, drug name, dose, administration route, timing, frequency, and duration, any antimicrobial resistance to antibiotics, any bone cementation with or without antibiotics or cementless fixation] [if applicable]).

(3) Baseline participant characteristics (e.g., age, sex, weight, BMI, comorbidities, indication for THA [e.g. osteoarthritis, acute or sequalae from proximal femoral or pelvic fractures, late sequalae of pediatric hip disorders, cancer or metastasis] [if applicable])

(4) Outcome data (e.g., number of participants experiencing at least one SAE, SSI and PJI, any serious infections, MACE, VTE and mortality occurring within 90 days and 1 year after index surgery in each intervention group. Total number of patients the outcome was measured in (when only the number of events instead of the number of subjects experiencing an event is reported, an assumption of one event per subject will be made), as well as the number of subjects experiencing each of the SAE subcomponents, and the total number of subjects for which the outcomes were measured.

Critical Appraisal, Risk of bias in individual studies (internal validity)

Two independent reviewers (AAA and HJMS) will assess the risk of bias within each included RCT using the revised Cochrane '*Risk of bias*' tool for randomized trials (RoB 2.0) (50). Included trials will be rated as having a low risk, high risk, or unclear risk of bias (where a lack of information is present); for each trial, the overall risk of bias is classified as low (that is, low risk of bias for all domains), high (that is, high risk of bias for one or more domains), or unclear (that is, unclear risk of bias for one or more domains in the absence of high risk of bias). A risk of bias table will be completed for each eligible study. Any disagreements among independent reviewing authors will be discussed to achieve a consensus. If necessary, disagreements may be resolved by a third author.

Measurements of treatment effect

Most trials will have only a few SAEs, so the odds ratios and 95% confidence intervals (95%CIs) will be calculated with the use of the Mantel-Haenszel Odds Ratios (with 95%CI) (51, 52). Forest plots will be displayed showing the individual study estimates and a summary estimate.

Dealing with missing data

For missing data for binary outcome variables, the original investigators will be contacted, and missing data will be requested. If not possible, an assumption will be made that the data is missing at random or not missing at random and the impact of the missing data will be calculated (53).

Assessment of heterogeneity

Statistical heterogeneity will be assessed through the forest plots by evaluation of the extent of overlap of confidence intervals. Heterogeneity between trials (i.e., proportion of variation in the combined estimates) will be assessed based on the Cochrane chi-square test, and interpreted based on the inconsistency index (I^2): For the interpretation of I^2, thresholds of 0% to 40% will be considered *not important*, >30% to 60% may represent *moderate heterogeneity*, >50% to 90%; *substantial heterogeneity*, and >75% to 100%; *considerable heterogeneity* (54). In cases of considerable heterogeneity (defined as $I^2 \geq 75\%$) data will need further exploration to explain heterogeneity.

Data synthesis

We will perform network meta-analyses (based on ORs) in order to assess the comparative effectiveness (55, 56). Traditional pair-wise meta-analyses (comparing Experimental antibiotic *vs* Control comparator) will be performed as well. We will use the Mantel-Haenszel method (52). and, where necessary, an continuity correction factor centered around 0·5 to handle zero cells. The analyses will be performed in the R software.

Potential sources of heterogeneity will be explored by application of the following prespecified stratified (subgroup) analyses or meta-regression analyses for the main outcome if sufficient studies are available:

1. type of implant (antibiotic-loaded bone cement versus bone cement with no antibiotic-load or cementless fixation)
2. type of antibiotic (beta-lactam antibiotics versus other and cephalosporins versus other)
3. route of antibiotic administration (intravenously versus perorally)
4. follow-up duration (90 days versus 90-360 days)
5. age (≥65 versus <65 years)
6. sex (male versus female)
7. different participants by classifying them in reason for THA in categories (Osteoarthritis and fracture and cancer or metastasis)
8. different participants by classifying them in DM and non-DM groups
9. different participants by classifying them in body mass index categories (≥30 versus <30 kg/m^2)

Small-study effects will be addressed using a comparison-adjusted funnel plot of treatment estimates (57). For the network meta-analysis, the different models of sensitivity analysis suggested by Warren et al. (58), will be explored. In cases of sparse networks, direct estimates will be applied as the best estimates of the treatment effects (59, 60).

Network Meta-analysis Extensions

Network Geometry and Further Considerations for Bias of the SAEs: Networks may take on different shapes. The term network geometry will be used to refer to the architecture of the treatment comparisons that have been made for the condition under study. This includes visuals illustrating what treatments are involved in the comparisons in a network, in what abundance they are present, the respective numbers of patients randomly assigned to each treatment, and whether particular treatments and comparisons may have been preferred or avoided.

Probabilities and Rankings in the Network Meta-analysis of the SAEs: We will use the network meta-analysis of SAEs to propose a hierarchy of the competing interventions in terms of treatment rankings. The ranking probabilities will refer to the probabilities estimated for each treatment in the network of achieving a particular placement in an ordering of treatment effects from best to worst.

Several techniques are possible when summarizing the relative rankings, including graphical tools. Robust reporting of rankings includes specifying median ranks with uncertainty intervals, cumulative probability curves and p-scores, Rankings will be reported along with corresponding estimates of pairwise comparisons between interventions.

The Assumption of Transitivity for Network Meta-analysis of SAEs: For network meta-analysis to produce valid results, it is important that the distribution of effect modifiers is similar, for example, across trials providing direct evidence. This balance increases the plausibility of reliable findings from an indirect comparison. When this balance is present, the assumption of transitivity can be judged to hold. We will assess the comparability of patient and study characteristics across the studies that compare pairs of treatments. These characteristics (possible effect modifiers) will include such traits as average patient age, gender distribution, average patient weight and BMI, comorbidities, reason for THA in categories (Osteoarthritis and fracture and cancer or metastasis) and type of implant (antibiotic-loaded bone cement versus bone cement with no antibiotic-load or cementless fixation), type of treatment (oral vs intravenous, type of antibiotic (beta-lactam antibiotics versus other and cephalosporins versus other) and follow-up period.

Network Meta-analysis and Assessment of Consistency of Findings for Network Meta-analysis of SAEs: Direct and indirect evidence for a comparison of interventions should be combined only when their findings are similar in magnitude and interpretation. The assumption of comparability of direct and indirect evidence will be referred to as consistency of treatment effects. When a treatment network contains a closed loop of interventions, it is possible to examine statistically whether there is agreement between the direct and indirect estimates of intervention effect. Different methods are available to evaluate potential differences in relative treatment effects estimated by direct and indirect comparisons are grouped as local approaches, such as node-splitting, and global approaches, such as global I^2 statistics (61)..

Confidence in cumulative evidence

The credibility of the network meta-analysis results will be examined using the Confidence in Network Meta-Analysis (CINeMA) tool. We will specifically assess six domains: (1) within-study bias, (2) reporting bias, (3) indirectness, (4) imprecision, (5) heterogeneity, and (6) incoherence (62, 63).

ETHICS AND DISSEMINATION

This is a protocol for a systematic review and meta-analysis of published data; therefore, ethics review and approval are not required. The findings of the study will be published in a peer-reviewed journal and disseminated at scientific conferences.

Registration: This study will be registered in PROSPERO prior to starting.

REFERENCES

1. Lefebvre C ME, Glanville J. Box 6.4.a: Cochrane Highly Sensitive Search Strategy for identifying randomized trials in MEDLINE: sensitivity-maximizing version (2008 revision); PubMed format. In: Higgins J GS, editor. Cochrane Handbook for Systematic Reviews of Interventions Version 510 (updated March 2011) The Cochrane Collaboration, 2011.
2. Glanville J, Foxlee R, Wisniewski S, Noel-Storr A, Edwards M, Dooley G. Translating the Cochrane EMBASE RCT filter from the Ovid interface to Embase.com: a case study. (1471-1842 (Electronic)).
3. Carlsson AK, Lidgren L, Lindberg L. Prophylactic antibiotics against early and late deep infections after total hip replacements. Acta Orthop Scand. 1977;48(4):405-10.
4. Kurtz S, Mowat F, Ong K, Chan N, Lau E, Halpern M. Prevalence of primary and revision total hip and knee arthroplasty in the United States from 1990 through 2002. J Bone Joint Surg Am. 2005;87(7):1487-97.
5. Birrell F, Johnell O, Silman A. Projecting the need for hip replacement over the next three decades: influence of changing demography and threshold for surgery. Ann Rheum Dis. 1999;58(9):569-72.
6. Singh JA. Epidemiology of knee and hip arthroplasty: a systematic review. Open Orthop J. 2011;5:80-5.
7. Zimmerli W, Trampuz A, Ochsner PE. Prosthetic-joint infections. N Engl J Med. 2004;351(16):1645-54.
8. Zmistowski B, Karam JA, Durinka JB, Casper DS, Parvizi J. Periprosthetic joint infection increases the risk of one-year mortality. J Bone Joint Surg Am. 2013;95(24):2177-84.
9. Bozic KJ, Ries MD. The impact of infection after total hip arthroplasty on hospital and surgeon resource utilization. J Bone Joint Surg Am. 2005;87(8):1746-51.
10. Osmon DR, Berbari EF, Berendt AR, Lew D, Zimmerli W, Steckelberg JM, et al. Executive summary: diagnosis and management of prosthetic joint infection: clinical practice guidelines by the Infectious Diseases Society of America. Clin Infect Dis. 2013;56(1):1-10.
11. Gundtoft PH, Pedersen AB, Varnum C, Overgaard S. Increased Mortality After Prosthetic Joint Infection in Primary THA. Clin Orthop Relat Res. 2017;475(11):2623-31.
12. Doyon F, Evrard J, Mazas F, Hill C. Long-term results of prophylactic cefazolin versus placebo in total hip replacement. Lancet. 1987;1(8537):860.
13. Hill C, Flamant R, Mazas F, Evrard J. Prophylactic cefazolin versus placebo in total hip replacement. Report of a multicentre double-blind randomised trial. Lancet. 1981;1(8224):795-6.
14. van Kasteren ME, Mannien J, Ott A, Kullberg BJ, de Boer AS, Gyssens IC. Antibiotic prophylaxis and the risk of surgical site infections following total hip arthroplasty: timely administration is the most important factor. Clin Infect Dis. 2007;44(7):921-7.
15. AlBuhairan B, Hind D, Hutchinson A. Antibiotic prophylaxis for wound infections in total joint arthroplasty: a systematic review. J Bone Joint Surg Br. 2008;90(7):915-9.

16. Thornley P, Evaniew N, Riediger M, Winemaker M, Bhandari M, Ghert M. Postoperative antibiotic prophylaxis in total hip and knee arthroplasty: a systematic review and meta-analysis of randomized controlled trials. CMAJ Open. 2015;3(3):E338-43.
17. Siddiqi A, Forte SA, Docter S, Bryant D, Sheth NP, Chen AF. Perioperative Antibiotic Prophylaxis in Total Joint Arthroplasty: A Systematic Review and Meta-Analysis. J Bone Joint Surg Am. 2019;101(9):828-42.
18. Voigt J, Mosier M, Darouiche R. Systematic review and meta-analysis of randomized controlled trials of antibiotics and antiseptics for preventing infection in people receiving primary total hip and knee prostheses. Antimicrob Agents Chemother. 2015;59(11):6696-707.
19. Berrios-Torres SI, Umscheid CA, Bratzler DW, Leas B, Stone EC, Kelz RR, et al. Centers for Disease Control and Prevention Guideline for the Prevention of Surgical Site Infection, 2017. JAMA Surg. 2017;152(8):784-91.
20. World Health O. Global guidelines for the prevention of surgical site infection. 2nd ed. ed. Geneva: World Health Organization; 2018 2018.
21. Bratzler DW, Dellinger EP, Olsen KM, Perl TM, Auwaerter PG, Bolon MK, et al. Clinical practice guidelines for antimicrobial prophylaxis in surgery. Am J Health Syst Pharm. 2013;70(3):195-283.
22. Parvizi J, Gehrke T, Mont MA, Callaghan JJ. Introduction: Proceedings of International Consensus on Orthopedic Infections. The Journal of Arthroplasty. 2019;34(2):S1-S2.
23. Hansen E, Belden K, Silibovsky R, Vogt M, Arnold W, Bicanic G, et al. Perioperative antibiotics. J Orthop Res. 2014;32 Suppl 1:S31-59.
24. Veltman ES, Lenguerrand E, Moojen DJF, Whitehouse MR, Nelissen R, Blom AW, et al. Similar risk of complete revision for infection with single-dose versus multiple-dose antibiotic prophylaxis in primary arthroplasty of the hip and knee: results of an observational cohort study in the Dutch Arthroplasty Register in 242,179 patients. Acta Orthop. 2020;91(6):794-800.
25. Branch-Elliman W, O'Brien W, Strymish J, Itani K, Wyatt C, Gupta K. Association of Duration and Type of Surgical Prophylaxis With Antimicrobial-Associated Adverse Events. JAMA Surg. 2019;154(7):590-8.
26. Tokarski AT, Karam JA, Zmistowski B, Deirmengian CA, Deirmengian GK. Clostridium difficile is common in patients with postoperative diarrhea after hip and knee arthroplasty. J Arthroplasty. 2014;29(6):1110-3.
27. Jacoby GA, Munoz-Price LS. The new beta-lactamases. N Engl J Med. 2005;352(4):380-91.
28. Laxminarayan R, Duse A, Wattal C, Zaidi AK, Wertheim HF, Sumpradit N, et al. Antibiotic resistance-the need for global solutions. Lancet Infect Dis. 2013;13(12):1057-98.
29. Rasouli MR, Maltenfort Mg Fau - Purtill JJ, Purtill Jj Fau - Hozack WJ, Hozack Wj Fau - Parvizi J, Parvizi J. Has the rate of in-hospital infections after total joint arthroplasty decreased? (1528-1132 (Electronic)).
30. Glassou EN, Hansen TB, Pedersen AB. Risk of pneumonia and urinary tract infection within the first week after total hip arthroplasty and the impact on survival. (1179-1349 (Print)).
31. Belmont PJ, Jr., Goodman GP, Hamilton W, Waterman BR, Bader JO, Schoenfeld AJ. Morbidity and mortality in the thirty-day period following total hip arthroplasty: risk factors and incidence. (1532-8406 (Electronic)).
32. Pulido L, Parvizi J, Macgibeny M, Sharkey PF, Purtill JJ, Rothman RH, et al. In hospital complications after total joint arthroplasty. J Arthroplasty. 2008;23(6 Suppl 1):139-45.
33. Parvizi J, Mui A Fau - Purtill JJ, Purtill Jj Fau - Sharkey PF, Sharkey Pf Fau - Hozack WJ, Hozack Wj Fau - Rothman RH, Rothman RH. Total joint arthroplasty: When do fatal or near-fatal complications occur? (0021-9355 (Print)).
34. Singh JA, Jensen MR, Harmsen WS, Gabriel SE, Lewallen DG. Cardiac and thromboembolic complications and mortality in patients undergoing total hip and total knee arthroplasty. Ann Rheum Dis. 2011;70(12):2082-8.
35. Lalmohamed A, Vestergaard P, Klop C, Grove EL, de Boer A, Leufkens HG, et al. Timing of acute myocardial infarction in patients undergoing total hip or knee replacement: a nationwide cohort study. Arch Intern Med. 2012;172(16):1229-35.
36. Cowan LT, Lutsey PL, Pankow JS, Matsushita K, Ishigami J, Lakshminarayan K. Inpatient and Outpatient Infection as a Trigger of Cardiovascular Disease: The ARIC Study. J Am Heart Assoc. 2018;7(22):e009683.
37. Meier CR, Jick SS, Derby LE, Vasilakis C, Jick H. Acute respiratory-tract infections and risk of first-time acute myocardial infarction. Lancet. 1998;351(9114):1467-71.
38. Corrales-Medina VF, Alvarez KN, Weissfeld LA, Angus DC, Chirinos JA, Chang CC, et al. Association between hospitalization for pneumonia and subsequent risk of cardiovascular disease. (1538-3598 (Electronic)).
39. Naghavi M, Wyde P, Litovsky S, Madjid M, Akhtar A, Naguib S, et al. Influenza infection exerts prominent inflammatory and thrombotic effects on the atherosclerotic plaques of apolipoprotein E-deficient mice. Circulation. 2003;107(5):762-8.
40. Levi M, van der Poll T Fau - Schultz M, Schultz M. Infection and inflammation as risk factors for thrombosis and atherosclerosis. (1098-9064 (Electronic)).
41. Levi M, Keller Tt Fau - van Gorp E, van Gorp E Fau - ten Cate H, ten Cate H. Infection and inflammation and the coagulation system. (0008-6363 (Print)).

42. Moher D, Shamseer L, Clarke M, Ghersi D, Liberati A, Petticrew M, et al. Preferred reporting items for systematic review and meta-analysis protocols (PRISMA-P) 2015 statement. Syst Rev. 2015;4:1.
43. Hutton B Fau - Salanti G, Salanti G Fau - Caldwell DM, Caldwell Dm Fau - Chaimani A, Chaimani A Fau - Schmid CH, Schmid Ch Fau - Cameron C, Cameron C Fau - Ioannidis JPA, et al. The PRISMA extension statement for reporting of systematic reviews incorporating network meta-analyses of health care interventions: checklist and explanations. (1539-3704 (Electronic)).
44. ICH HARMONISED GUIDELINE. INTEGRATED ADDENDUM TO ICH E6(R1): GUIDELINE FOR GOOD CLINICAL PRACTICE E6(R2) [Internet]. 2016 [cited 12 July 2022]. Available from: https://database.ich.org/sites/default/files/E6_R2_Addendum.pdf.
45. (NHSN) NHSN. NHSN Surgical Site Infection Event (SSI) January 2022.pdf. 2022.
46. Bruce J, Russell EM, Mollison J, Krukowski ZH. The measurement and monitoring of surgical adverse events. Health Technol Assess. 2001;5(22):1-194.
47. Schnell O, Rydén L, Standl E, Ceriello A. Current perspectives on cardiovascular outcome trials in diabetes. (1475-2840 (Electronic)).
48. Patel T, Tesfaldet B, Chowdhury I, Kettermann A, Smith JP, Pucino F, et al. Endpoints in diabetes cardiovascular outcome trials. (1474-547X (Electronic)).
49. Hicks KA, Mahaffey KW, Mehran R, Nissen SE, Wiviott SD, Dunn B, et al. 2017 Cardiovascular and Stroke Endpoint Definitions for Clinical Trials. (1524-4539 (Electronic)).
50. Higgins JPT SJ, Page MJ, Elbers RG, Sterne JAC. Chapter 8: Assessing risk of bias in a randomized trial. In: Higgins JPT, Thomas J, Chandler J, Cumpston M, Li T, Page MJ, Welch VA (editors). : Cochrane 2022; 2022.
51. Bradburn M, Deeks J, Berlin J, Localio A. Much ado about nothing: A comparison of the performance of meta-analytical methods with rare events. Statistics in medicine. 2007;26:53-77.
52. Efthimiou OA-O, Rücker GA-O, Schwarzer GA-O, Higgins JPT, Egger M, Salanti GA-O. Network meta-analysis of rare events using the Mantel-Haenszel method. (1097-0258 (Electronic)).
53. Yuan Y, Little RJ. Meta-analysis of studies with missing data. Biometrics. 2009;65(2):487-96.
54. Higgins JP, Thompson SG, Deeks JJ, Altman DG. Measuring inconsistency in meta-analyses. BMJ. 2003;327(7414):557-60.
55. Platt RW, Leroux Bg Fau - Breslow N, Breslow N. Generalized linear mixed models for meta-analysis. (0277-6715 (Print)).
56. Salanti G, Higgins Jp Fau - Ades AE, Ades Ae Fau - Ioannidis JPA, Ioannidis JP. Evaluation of networks of randomized trials. (0962-2802 (Print)).
57. Chaimani A, Salanti G. Using network meta-analysis to evaluate the existence of small-study effects in a network of interventions. (1759-2879 (Print)).
58. Warren FC, Abrams Kr Fau - Sutton AJ, Sutton AJ. Hierarchical network meta-analysis models to address sparsity of events and differing treatment classifications with regard to adverse outcomes. (1097-0258 (Electronic)).
59. Brignardello-Petersen R, Mustafa RA, Siemieniuk RAC, Murad MH, Agoritsas T, Izcovich A, et al. GRADE approach to rate the certainty from a network meta-analysis: addressing incoherence. (1878-5921 (Electronic)).
60. Brignardello-Petersen R, Murad MH, Walter SD, McLeod S, Carrasco-Labra A, Rochwerg B, et al. GRADE approach to rate the certainty from a network meta-analysis: avoiding spurious judgments of imprecision in sparse networks. (1878-5921 (Electronic)).
61. van Valkenhoef G, Dias S, Ades AE, Welton NJ. Automated generation of node-splitting models for assessment of inconsistency in network meta-analysis. Res Synth Methods. 2016;7(1):80-93.
62. Nikolakopoulou A, Higgins JA-O, Papakonstantinou TA-O, Chaimani AA-O, Del Giovane C, Egger MA-O, et al. CINeMA: An approach for assessing confidence in the results of a network meta-analysis. (1549-1676 (Electronic)).
63. Papakonstantinou T, Nikolakopoulou A, Higgins J, Egger M, Salanti G. CINeMA: Software for semiautomated assessment of the confidence in the results of network meta-analysis. Campbell Systematic Reviews. 2020;16.
